# Circulating miR-206 and Wnt-signaling are associated with cardiovascular complications and a history of preeclampsia in women

**DOI:** 10.1042/CS20190920

**Published:** 2020-01-21

**Authors:** Kenny Schlosser, Amanpreet Kaur, Natalie Dayan, Duncan J. Stewart, Louise Pilote, Christian Delles

**Affiliations:** 1 Sinclair Centre for Regenerative Medicine, Ottawa Hospital Research Institute, Ottawa, Ontario, Canada; 2 Research Institute, McGill University Health Centre, Montreal, Quebec, Canada; 3 Department of Medicine, Division of General Internal Medicine, McGill University Health Centre, Montreal, Quebec, Canada; 4 Department of Cellular and Molecular Medicine, University of Ottawa, Ottawa, Ontario Canada; 5 Institute of Cardiovascular and Medical Sciences, University of Glasgow, Scotland, U.K.

**Keywords:** acute coronary syndrome, microRNA, preeclampsia, RNA sequencing

## Abstract

Women with a history of preeclampsia (PE) have increased risk of cardiovascular disease (CVD) later in life. However, the molecular determinants underlying this risk remain unclear. We sought to understand how circulating miRNA levels are affected by prior PE, and related to biological pathways underpinning cardiovascular disease. RNA sequencing was used to profile plasma levels of 2578 miRNAs in a retrospective study of women with a history of PE or normotensive pregnancy, in two independent cohorts with either acute coronary syndrome (ACS) (*n* = 17–18/group) or no ACS (*n* = 20/group). Differential miRNA alterations were assessed in relation to a history of PE (within each cohort) or ACS (across cohorts), and compared with miRNAs previously reported to be altered during PE. A history of PE was associated with altered levels of 30 and 20 miRNAs in the ACS and non-ACS cohorts, respectively, whereas ACS exposure was associated with alterations in 259 miRNAs. MiR-206 was identified at the intersection of all comparisons relating to past/current PE and ACS exposure, and has previously been implicated in atherogenic activities related to hepatocytes, vascular smooth muscle cells and macrophages. Integration of all differentially altered miRNAs with their predicted and experimentally validated targets *in silico* revealed a number of highly targeted genes with potential atherogenic functions (including NFAT5, CCND2 and SMAD2), and one significantly enriched KEGG biological pathway (Wnt signaling) that was shared between all exposure groups. The present study provides novel insights into miRNAs, target genes and biological pathways that may underlie the long-term cardiovascular sequelae of PE.

## Introduction

Preeclampsia (PE) is estimated to affect up to 8% of all pregnancies worldwide, and thus represents a substantial burden on the cardiovascular health of millions of women [1]. While the high blood pressure and proteinuria associated with PE usually resolve after placental delivery, women are left with a higher risk of premature cardiovascular complications and mortality [1]. Elevated rates of metabolic syndrome and hypertension have been reported after preeclamptic pregnancies [2]. In addition, vascular abnormalities in arterial stiffness revealed by pulse wave velocity and other metrics have been reported in women with a history of PE [3]. Notwithstanding these broader pathophysiological insights, the underlying molecular determinants that lead to elevated risk of cardiovascular disease (CVD) following PE remain unclear.

MicroRNAs (miRNAs) are small non-coding RNAs that bind to the messenger RNAs of protein-coding genes in a sequence-dependent fashion to direct their repression. To date, over 2500 mature human miRNAs are known to exist, and each may have the capacity to regulate hundreds of different genes to support diverse biological roles [4,5]. A number of miRNAs have been implicated causally in cardiovascular health and disease [6,7], and altered levels of systemic circulating miRNAs (as identified in blood plasma or serum) have also been investigated as potential biomarkers and/or messengers of disease activity and signaling [8,9]. While a large number of studies have reported acute changes in the levels of circulating and/or placental miRNAs during PE [10], relatively little is known about the long-term impact of PE on cardiovascular health. Thus, we have previously sought to understand how dysregulation of miRNA expression, manifested through alterations in circulating levels, may contribute to CVD activity even after PE is resolved [11]. In a limited screen of 372 predefined miRNAs commonly found in circulation, we identified alterations in several potential atherogenic miRNAs in a cohort of women with premature acute coronary syndrome (ACS) and a history of PE or normotensive (NT) pregnancy [11]. These miRNAs have previously been implicated in endothelial dysfunction, angiogenesis and inflammation, consistent with their potential contribution to cardiovascular abnormalities.

In the present study, we sought to identify miRNAs and biological pathways that may contribute to the long-term cardiovascular sequelae of PE, using a far more comprehensive platform to investigate alterations in circulating miRNAs. Next-generation RNA sequencing was used to probe global changes in plasma levels of all 2578 mature human miRNAs (annotated in miRBase V.20), in two independent cohorts of women with and without a history of PE versus NT control pregnancy. The first cohort consisted of women with premature ACS and many traditional atherosclerotic risk factors. A second cohort of similarly aged women with no acute ischemic event and significantly lower burden of CVD risk factors was used to probe the generalizability of candidate miRNAs associated with prior PE, independent of overt CVD and atherosclerotic risk factors. Differentially altered circulating miRNAs were identified in relation to prior PE and current ACS exposure, combined with their predicted and experimentally validated gene targets and used in a pathway enrichment analysis to identify shared mechanisms linking PE to CVD.

## Methods

### Cohort 1: ACS subjects with a history of PE versus NT pregnancy

Women with premature acute coronary syndrome (ACS) and a history of PE or NT pregnancy were identified from the GENESIS-PRAXY multicentre study of adults hospitalized with ACS. Detailed methods have been previously described [12]. Participants were previously recruited between 2009 and 2013 from 24 centers across Canada, one in the US and one in Switzerland. All participating sites received ethics approval from their respective ethics review boards, and participants provided written informed consent. Eligible participants were aged 18–55 years diagnosed with ACS, able to provide informed consent and had sufficient plasma specimens drawn at study entry (*n* = 30 with prior preeclampsia and *n* = 146 with prior normotensive pregnancy). A detailed self-reported questionnaire was used to collect pregnancy data from all female participants at study entry, and the classification of prior PE was made if they reported either PE or high blood pressure in addition to proteinuria. Subjects were excluded if they were unsure about the presence or absence of a pregnancy complication or if completion of these questions was incomplete. The time since last pregnancy was estimated using the age of the youngest biological child, to serve as a proxy for the interval between pregnancy and incident ACS. Venipuncture was performed on all consenting participants within 24 h of hospital admission for ACS. Whole blood collected in citrate Vacutainers was spun at 4°C at 3000 rpm for 10 min and the plasma supernatant removed and frozen at −80°C. A total of 40 subjects were selected for miRNA sequencing (*n* = 20 subjects/exposure group) after matching for CVD risk factors including hypertension, diabetes, smoking and age. The final sample size was reduced to *n* = 17–18 subjects/exposure group after several plasma specimens failed RNA- or library- quality control tests prior to sequencing (details provided in the online data supplement).

### Cohort 2: Non-ACS subjects with a history of PE versus NT pregnancy

Women without ACS and a history of either PE or NT pregnancy were identified from The Cardiovascular Consequences of Pre-eclampsia (COPS) study at the British Heart Foundation Glasgow Cardiovascular Research Centre (BHF GCRC), which previously recruited 86 women with a history of PE and 80 NT pregnancy controls. Women were recruited from multiple sources including the previous Generation Scotland: Scottish Family Health Study [13], the Proteomics in Pre-eclampsia study [14], patients who attended blood pressure clinics and friends and colleagues of participants who contacted us with interest in participating. The study was approved by the West of Scotland Research Ethics Committee 3 (Reference 12/WS/0306), and participants provided written informed consent. The index pregnancy was defined as the first pregnancy in NT women and the first pre-eclamptic pregnancy in those with PE. Women were excluded if they were >60 years old, already had established cardiovascular disease or if they were unable to give informed consent. Participants completed a questionnaire asking for obstetric history, past medical history, drug history, smoking history and family history. Blood samples were taken from the antecubital fossa using a standard tourniquet and Vacutainer system, and centrifuged at 4°C at 2500 rpm for 15 min and plasma supernatant removed and frozen at −80°C. A total of 40 subjects (*n* = 20 subjects/exposure group) matched on hypertension, diabetes, and age were selected and used for miRNA sequencing after passing all quality control tests.

### Cohort 3: ACS versus non-ACS subjects

The comparison of all subjects in cohort 1 (*n* = 35 total ACS subjects) versus cohort 2 (*n* = 40 total non-ACS subjects) was used to assess potential associations between ACS (including related risk factors)and circulating miRNA levels.

### Cohort 4: Women with PE versus NT pregnancy

Information for this cohort was derived from six prior independent studies of preeclamptic women (with no ACS) reported in the systematic review by Sheikh et al. [10]. A total of 104 circulating miRNAs were identified via high-throughput screening methods as differentially altered in plasma, serum or whole blood collected from women during pregnancies complicated by PE versus NT control pregnancies.

### RNA isolation and quality control, library construction/quality control and sequencing, sequence trimming, UMI-consolidation and Data Mapping, miRNA-target integration and pathway enrichment analysis, pre- and post-study sample size and power estimations

Details are provided in the online data supplement including quality control assessment of extracted RNA (Supplementary Figure S1) and sequencing libraries (Supplementary Figure S2).

### Statistical analysis

All statistical tests comparing cohort characteristics were performed in Graphpad Prism 8.0. Data normality was assessed using the D’Agostino Pearson test. Differences between exposure groups for continuous data were assessed using a Mann–Whitney or unpaired *t*-test, depending on data normality as appropriate. Differences in categorical variables were assessed via Fisher’s exact test. Data are presented as mean ± standard deviation (SD) unless otherwise specified. Differential expression analysis was conducted on the subset of samples related to the specific groups being compared, using UMI-corrected miRNA counts as input into the EdgeR statistical software package (Bioconductor, http://www.bioconductor.org/). Data were preprocessed to exclude poorly detectable miRNAs such that the sum of the counts per million mapped reads (CPM) for each miRNA in all samples pertaining to the comparison subset was >10. The filtered data were normalized using the trimmed mean of *M*-values (TMM) normalization method in EdgeR to compensate for sample specific effects related to variations in sequencing depth and/or RNA composition. MiRNA levels in some figures are presented simply as counts per million mapped reads (CPM), which only correct for sequencing depth. *P*-values and Benjamini–Hochberg false discovery rate (FDR)-correct *P* values for differentially altered miRNAs were calculated with an exact test assuming a negative binomial distribution in EdgeR. Differentially altered miRNAs were defined by *P* < 0.05 (for cohorts 1 and 2 with prior exposure events) and FDR < 0.05 (for cohort 3 with a current exposure event). Principal component analysis and unsupervised hierarchical clustering and heatmap construction were performed with default parameters in Partek Genomics Suite 7.2 using log2 transformed TMM-normalized miRNA counts (with offset 1 to account for 0 values). Study-specific post hoc estimations of miRNA dispersion (a measure of variability in miRNA levels), sample size and statistical power for the completed sequencing experiment were calculated with the RNASeqSampleSize software package in R language and online interface at http://cqs.mc.vanderbilt.edu/shiny/RnaSeqSampleSize/. [15]

## Results

### Two independent cohorts of women with or without premature ACS, and a history of preeclampsia or normotensive pregnancy

The impact of prior PE on circulating miRNA levels was assessed in two cohorts of women with (cohort 1, C1) or without (cohort 2, C2) premature ACS. Within each cohort, subjects were closely matched and showed no significant differences in baseline characteristics between the prior PE versus NT pregnancy groups including age, body mass index (BMI), menopausal status and several cardiovascular disease (CVD) risk factors (Table 1). At the time of plasma sampling, an average of 19.7 ± 10.7 years had passed since the index pregnancy event, as measured across all subjects in both cohorts (*n* = 75). Between cohorts, ACS and non-ACS subjects were similarly aged, and showed no significant differences in the time from index pregnancy, or proportion of primiparous and menopausal women. Both cohorts were comprised primarily of Caucasian women, though the proportion of Caucasians in cohort 2 was significantly higher than cohort 1. In addition, the ACS cohort contained a significantly higher proportion of subjects with traditional atherosclerotic risk factors including elevated BMI, current smokers, hypertension, diabetes, dyslipidemia and history of coronary artery disease (Table 1).

**Table 1:** Cohort characteristics

Characteristics	Cohort 1 (ACS)			Cohort 2 (non-ACS)			ACS versus non-ACS
	PE (*n* = 18)	NT (*n* = 17)	*P*-val	PE (*n* = 20)	NT (*n* = 20)	*P*-val	*P*-val
Age (years)^†^	49 ± 6	48 ± 6	0.70	47 ± 11	49 ± 11	0.55	0.81
Sex, % female	100	100	1	100	100	1	1
Caucasian, *n* (%)	15 (83%)	12 (71%)	0.44	20 (100%)	20 (100%)	>0.99	0.001
Time since index pregnancy (yr)^†^	16 ± 9 (*n* = 14)	22 ± 8 (*n* = 16)	0.10	19 ± 11	22 ± 13	0.34	0.35
Primiparous, *n* (%)	5 (28%)	2 (12%)	0.40	4 (20%)	5 (25%)	>0.99	>0.99
Menopausal, *n* (%)	11 (61%)	7 (41%)	0.32	7 (35%)	11 (55%)	0.34	0.65
**CV risk factors**							
BMI (kg/m^2^)^†^	33 ± 12.4	35 ± 13.4	0.53	27.5 ± 4.0	26.6 ± 5.0	0.54	0.005
Current smoker, *n* (%)	6 (33%)	5 (29%)	>0.99	1 (5%)	2 (10%)	>0.99	0.02
Ex smoker, *n* (%)	9 (50%)	8 (47%)	>0.99	9 (45%)	5 (25%)	0.32	0.25
Hypertension, *n* (%)	14 (78%)	13 (76%)	>0.99	4 (20%)	1 (5%)	0.34	<0.0001
Diabetes, *n* (%)	2 (11%)	5 (29%)	0.23	0 (0)	0 (0)	>0.99	0.003
Dyslipidemia, *n* (%)	14 (78%)	8 (47%)	0.09	0 (0)	2 (10%)	0.49	<0.0001
History of CAD, *n* (%)	6 (33%)	8 (47%)	0.50	0 (0)	0 (0)	>0.99	<0.0001
Systolic BP^†^ (mmHg)	134.1 ± 25.9	134.1 ± 15.2 (*n* = 16)	0.99	129.8 ± 14.7	124.6 ± 11.1	0.22	0.09
Diastolic BP^†^ (mmHg)	82.2 ± 14.2	81.6 ± 13.6 (*n* = 16)	0.89	80.8 ± 9.3	77.3 ± 6.3	0.14	0.26
**Biomarkers**							
Standardized troponin (µg/l)^†^	8.5 ± 22.5 (*n* = 13)	30.1 ± 67.8	0.18	nd	nd		n/a
C-reactive protein (mg/l)^†^	38.4 ± 64.7 (*n* = 17)	32.1 ± 45.5 (*n* = 16)	0.97	nd	nd		n/a
**ACS type**							
STEMI	12 (66%)	5 (29%)	0.04	n/a	n/a		n/a
non-STEMI	6 (33%)	9(53%)	0.31	n/a	n/a		n/a
Unstable angina	0 (0)	3 (18%)	0.10	n/a	n/a		n/a

ACS: acute coronary syndrome

PE: prior preeclampsia

NT: prior normotensive pregnancy

† Mean ± SD

n/a: not applicable

nd: not determined

Additional sample sizes are noted where data were not available for all subjects.

### Alterations in circulating miRNA levels associated with a history of PE or current ACS exposure

**Figure 1:**
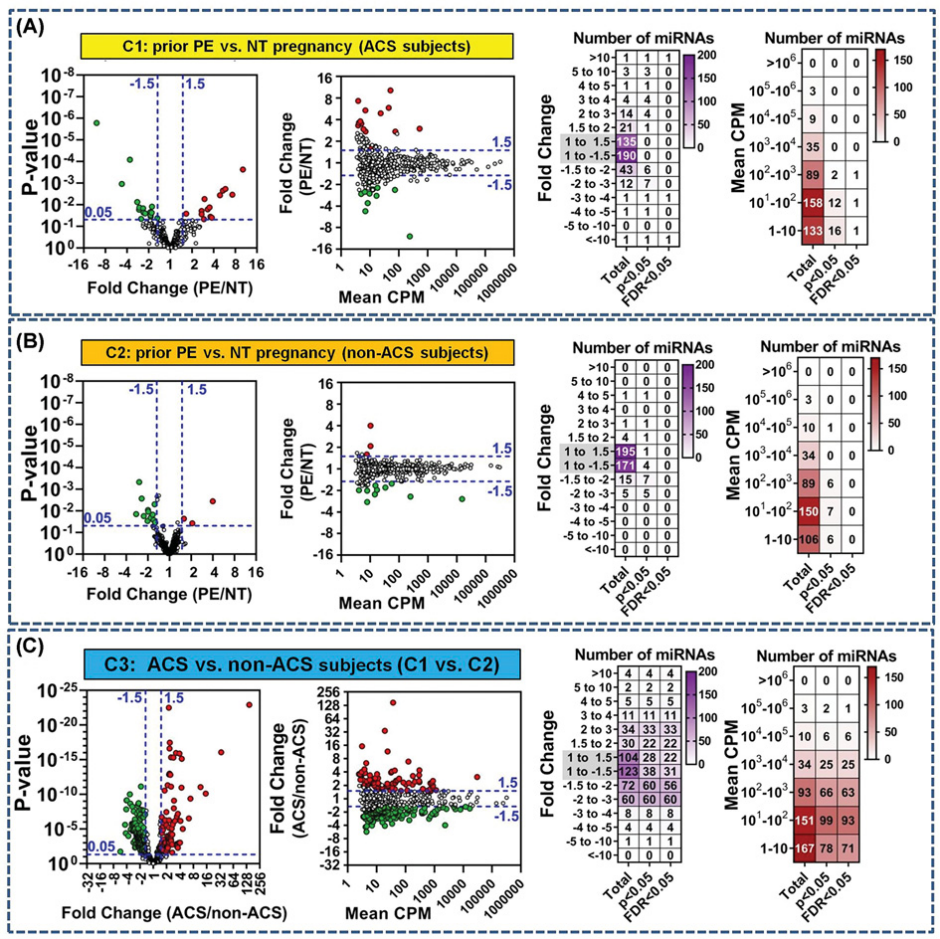
Differentially altered plasma miRNAs in women with and without a history of preeclampsia (PE), and in relation to acute coronary syndrome (ACS) (**A**) Plasma miRNA levels were assessed by next-generation RNA sequencing and differential analyses performed in (A) women with ACS (cohort 1) and a history of preeclampsia or normotensive (NT) pregnancy (*n* = 17–18/group), (**B**) similarly aged women without ACS (cohort 2) and a history of PE versus NT pregnancy (*n* = 20/group), and (**C**) women with versus without ACS (cohort 1 vs. cohort 2; *n* = 35–40/group). Graphs in panels (A–C) show the distribution of miRNAs (dots) according to unadjusted *P*-values, magnitude of fold change between exposure groups, and plasma level (expressed as the mean counts per million mapped reads, CPM). MiRNAs with *P* < 0.05 are color-coded green or red to denote decreased or increased plasma level, respectively. Specific *P* values for each miRNA are provided in Supplementary Tables S1–S3. Heatmaps in panels (A–C) show details on the number of miRNAs according to specific intervals of fold change or mean CPM for all detected miRNAs (total), and subsets of differentially altered miRNAs based on different statistical thresholds of *P* < 0.05 or FDR < 0.05.

On average, 455 miRNAs (range 271–586) were detected per sample across cohorts 1 and 2 (Supplementary Figure S3). In ACS cohort 1, 30 miRNAs were differentially altered between women with prior PE and NT pregnancy (*P* = 1.6 × 10^−6^ to 0.05), including three miRNAs that passed a false discovery rate threshold < 0.05 (Figure 1A and Supplementary Table S1). The absolute magnitude of miRNA changes ranged from 1.5- to 10-fold in both directions (median magnitude of change of 2.8-fold) (Figure 1A and Supplementary Table S1). In non-ACS cohort 2, a total of 20 miRNAs were differentially altered in relation to prior PE exposure (*P* = 0.0005–0.05), though none remained significant with FDR < 0.05. The magnitude of observed changes in miRNA levels ranged from 1.2- to 4-fold in cohort 2 (median magnitude of change of 1.8-fold) (Figure 1B and Supplementary Table S2). In both cohorts 1 and 2, the majority of differentially altered miRNAs were detected at less than 100 counts per million mapped reads (Figure 1A,B). To assess the association with ACS, differential alterations in plasma miRNA levels were also evaluated across cohorts 1 and 2 (this comparison is defined as cohort 3, C3, for clarity and labelling purposes). There were marked differences in the number of altered miRNAs, statistical significance and effect sizes related to ACS exposure as compared with prior PE exposure (Figure 1C and Supplementary Table S3). A total of 272 miRNAs were differentially altered (*P* < 0.05) in ACS compared with non-ACS subjects (cohort 1 vs. cohort 2), including 259 miRs with FDR < 0.05. Nominal *P* values for differentially altered miRNAs in cohort 3 ranged as low as 1.2 × 10^−23^, and effect sizes ranged from 1.2- to 148-fold in magnitude (median change of 1.9-fold). The majority of differentially altered miRNAs associated with ACS exposure were detected at less than 100 CPM.

**Figure 2:**
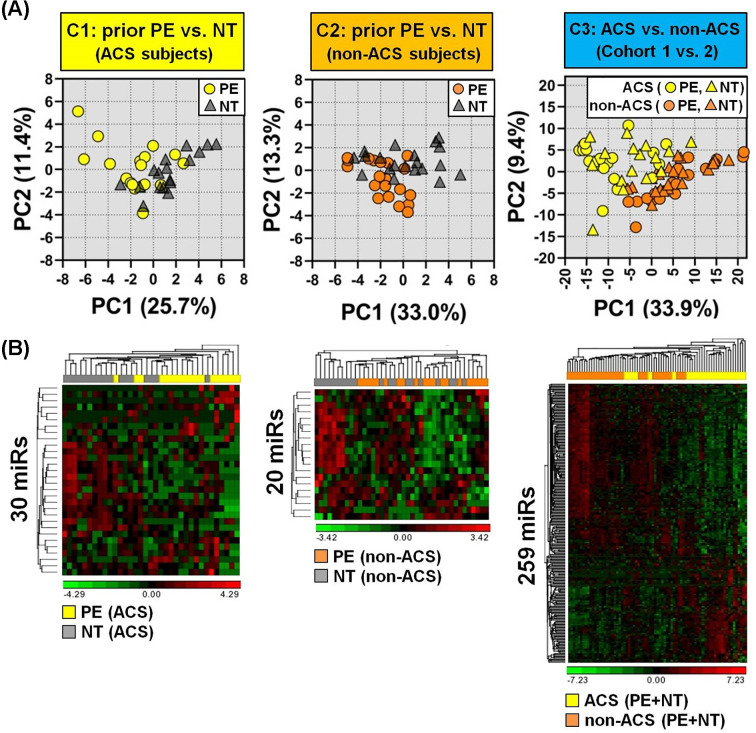
Principal component analysis (PCA) and hierarchical clustering of miRNAs associated with a history of PE or current ACS exposure (**A**) PCA of 30 differentially altered miRs (*P* < 0.05) in ACS cohort 1 (*n* = 17–18 subjects/group), 20 altered miRs (*P* < 0.05) in non-ACS cohort 2 (*n* = 20 subjects/group), and 259 altered miRs (FDR < 0.05) in cohort 3 comparing ACS versus non-ACS subjects (*n* = 35–40 /group). (**B**) Unsupervised hierarchical clustering and heatmap of plasma levels of differentially altered miRNAs for each exposure. Rows denote miRNAs and columns denote subjects. High and low relative plasma levels are denoted by red and green, respectively. Log2 transformed TMM-normalized miRNA counts (offset 1) were used for PCA, and further shifted to mean 0 and scaled to a standard deviation of 1 for clustering and heatmap construction.

For exploratory purposes and to gain further insight into how the biological features of prior PE (cohorts 1 and 2) and current ACS exposure (cohort 3) relate to the variability in miRNA profiles between subjects, a principal component analysis (PCA) was conducted with the differentially altered miRNAs identified in each cohort. This would allow visual assessment of the relative similarities or differences between subjects on the basis of their plasma miRNA signatures. While cohorts 1 and 2 showed some general clustering according to prior PE or NT pregnancy, there was marked overlap between the exposure groups (Figure 2). By comparison, subjects in cohort 3 appeared more broadly dispersed within clusters according to ACS or non-ACS exposure, but the separation between exposure groups was more prominent (Figure 2A). Unsupervised hierarchical clustering provided similar insights into the heterogeneity of patient miRNA profiles within and between exposure groups (Figure 2B).

We next sought to evaluate the relationship between statistical power, effect size and sample size for the completed sequencing experiment to better inform study outcomes. Since statistical power depends largely on the variability in miRNA levels, which might differ from our original estimates that were based on previously published data [11], we quantified the dispersion in miRNA levels in the present study. Supplementary Figure S4A shows the distribution of dispersion levels as determined by the RNASeqSampleSize program [15]. Although ACS cohort 1 showed a wider range of dispersion levels (from 0.1 to 11.4) as compared with non-ACS cohort 2 (from 0.1 to 4.1), the largest proportion of miRNAs in both cohorts occurred at a dispersion bin center of 0.5 in the frequency histogram (with 39% and 75% of all miRNAs exhibiting a dispersion ≤ 0.5 in cohort 1 and 2, respectively). Therefore, we investigated the relationship between statistical power, sample size and effect size assuming a common dispersion level of 0.5, and corrected for multiple testing of 400 miRNAs with FDR < 0.05. The corresponding power curve (Supplementary Figure S4B) indicates the current study had 85% power (with *n* = 20 subjects per group) to detect a 2.5-fold or greater change in plasma level for the majority of miRNAs, but was relatively underpowered to detect smaller effect sizes that might be associated with a history of PE. However, the power curve suggests a sample size of ∼100 subjects/group would be required to detect a 1.5-fold change in miRNA level (with FDR < 0.05 and 80% power), which exceeds the number of patient samples available in each of our archived cohorts.

### miR-206 levels are altered in multiple cohorts related to preeclampsia and acute coronary syndrome

**Figure 3:**
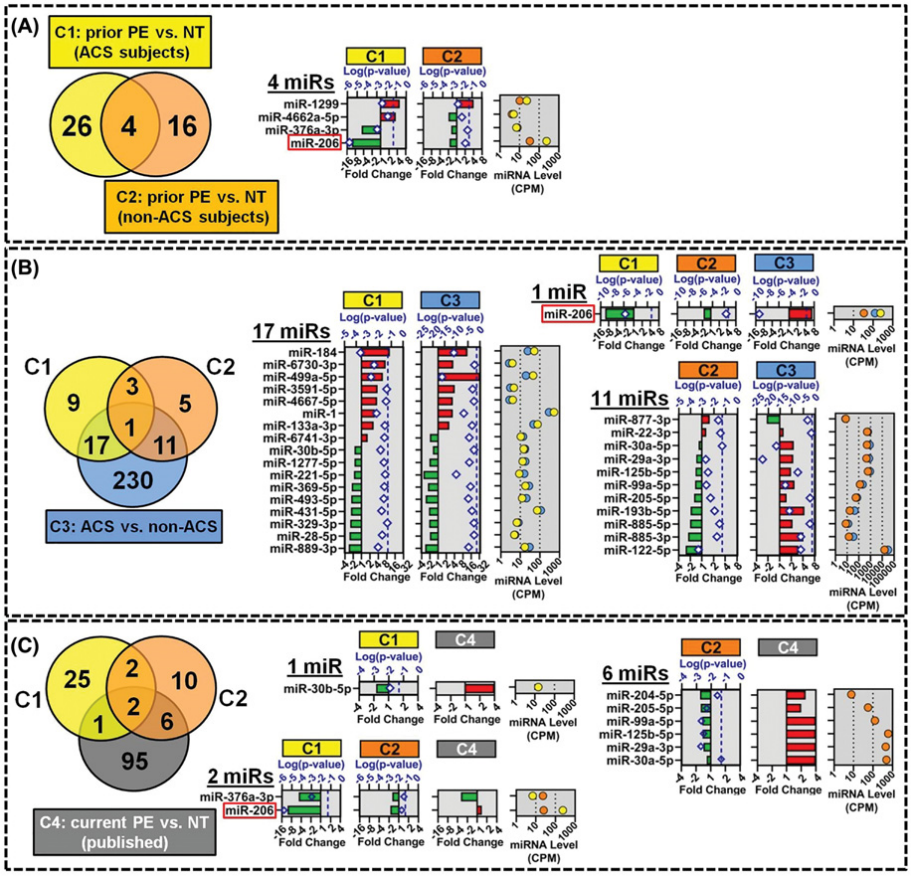
Overlap and identity of circulating miRNAs associated with prior PE or current ACS exposure (**A**) Venn diagram shows the overlap between 30 and 20 miRNAs that were differentially altered (*P* < 0.05) between women with history of PE versus NT pregnancy in cohorts 1 (*n* = 17–18/group) and 2 (*n* = 20/group), respectively. Right panel shows the identity of the 4 overlapping miRNAs with their direction and magnitude of change (bottom axis scale) and log10 transformed *P*-values (diamonds, top axis scale) separated by exposure cohort (ACS cohort 1, C1; non-ACS cohort 2, C2). Dashed vertical line denotes *P* = 0.05 threshold. Plasma miRNA levels are shown as mean counts per million mapped reads (CPM). Identities of all miRNAs and FDR-adjusted p-values are shown in Supplementary Table S4. (**B**) Venn diagram shows overlap among differentially altered miRNAs associated with prior PE in ACS subjects (C1: 30 miRs *P* < 0.05), prior PE in non-ACS subjects (C2; 20 miRs *P* < 0.05), and current ACS exposure (C3; 259 miRs FDR < 0.05, *n* = 35–40/group). Right panels show the identity, fold-change, *P*-values and CPM plasma level for miRNAs that intersect multiple exposure comparisons. Identities of all miRNAs and FDR-adjusted *P*-values are shown in Supplementary Table S5. (**C**) Venn diagram shows overlap among differentially altered miRNA candidates associated with prior PE in ACS (C1) or non-ACS (C2) subjects, and 104 miRNAs (C4) previously reported to be altered in circulation during PE [10]. Right panels show details on overlapping miRNAs. *P*-values and CPM levels for previously published data are not shown. Identities of all miRNAs and FDR-adjusted *P*-values are shown in Supplementary Table S7.

The overlap between differentially altered miRNAs identified in each cohort was investigated to prioritize miRNAs with higher biological relevance. Four miRNAs were altered in relation to a history of PE in both the ACS and non-ACS cohorts (cohorts 1 and 2, respectively), including three miRNAs that showed the same direction of change (miR-206 and miR-376a-3p decreased, and miR-1299 increased; Figure 3A and Supplementary Table S4). To help discern potential molecular mechanisms linking prior PE to CVD, we also examined the overlap with miRNAs that were differentially altered at the time of ACS (cohort 3; Figure 3B and Supplementary Table S5). Eighteen of thirty miRNAs that were differentially altered in ACS subjects with a history of PE (versus prior NT pregnancy; cohort 1) were also altered between ACS and non-ACS subjects (cohort 3 comparison), and the majority of these miRNAs (16 of 18) showed concordant directions of change. Of note, several miRNAs that have previously been associated with acute myocardial infarction including miR-1, miR-133a-3p and miR-499a-5p were also identified in the present study and showed concordant increases in plasma levels in both cohorts 1 and 3. In non-ACS subjects (cohort 2), 12 of 20 miRNAs that were associated with prior PE exposure were also differentially altered in cohort 3 in relation to ACS exposure (Figure 3B and Supplementary Table S5); however, the direction of change differed between cohorts for the majority of these miRNAs (11 of 12). Only one miRNA, miR-206, was differentially altered in all three cohorts, with decreased levels observed in relation to prior PE exposure and increased levels with ACS exposure.

We next sought to investigate whether the differential miRNA levels were induced at the time of the original PE event, or alternatively reflect progressive changes that occurred after resolution of PE. To address these different possibilities we cross-referenced miRNAs identified in the present study (i.e. assessed ∼20 years after the index pregnancy event on average) with miRNAs previously reported to be altered in circulation at or near the time of PE or NT pregnancy [10]. A total of 104 unique circulating miRNAs were identified and curated from six prior screening studies of preeclamptic women [10] (herein defined as cohort 4, C4, for clarity and labeling purposes; Supplementary Table S6). Three of these miRNAs were shared with ACS cohort 1, 8 miRNAs were shared with non-ACS cohort 2, and only 2 miRNAs (miR-206 and miR-376a-3p) were common between all three cohorts, though the direction of regulation differed between prior and current PE exposures (Figure 3C and Supplementary Table S7).

### miRNA–gene target integration and pathway enrichment analysis reveal Wnt signaling as a common pathway associated with PE and ACS

**Figure 4:**
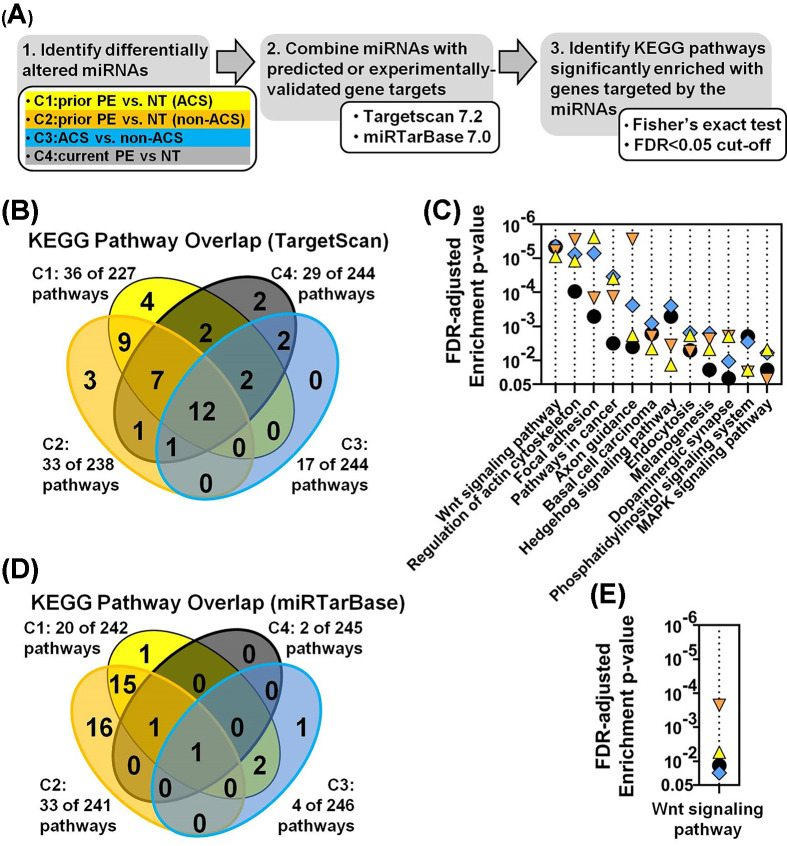
MiRNA target gene integration and pathway enrichment analysis (**A**) Workflow for identifying KEGG biological pathways significantly enriched with genes targeted by the differentially altered miRNAs. Target integration was conducted with 30 miRs (*P* < 0.05), 20 miRs (*P* < 0.05) and 259 miRs (FDR < 0.05) for cohort 1 (C1: prior PE vs. NT pregnancy in ACS subjects), cohort 2 (C2: prior PE vs. NT pregnancy in non-ACS subjects) and cohort 3 (C3: ACS vs. non-ACS subjects), respectively. A set of 104 miRNAs curated from several previous independent screening studies of miRNA alterations observed in plasma/serum during PE were also assessed (C4: current PE vs. NT pregnancy). Predicted and experimentally validated gene targets were identified using Targetscan7.2 and miRTarBase7.0 databases, respectively. (**B**) Venn diagram showing the number of KEGG pathways significantly enriched (FDR < 0.05) with the predicted gene targets of the altered miRNAs, and the extent of overlap between different exposures. (**C**) Identities of the 12 KEGG pathways at the intersection of all 4 cohorts from panel (B), and associated false discovery rate (FDR)-adjusted enrichment *P*-values for each cohort (C1-triangle; C2-inverted triangle; C3-diamond; C4-circle). (**D**) Venn diagram showing number of pathways significantly enriched (FDR < 0.05) with experimentally-validated gene targets of altered miRNAs. (**E**) Identity of the single pathway at the intersection of all 4 cohorts from panel (D). The identities of all significantly enriched pathways are presented in Supplementary Figures S5 and S6.

Since small changes in multiple miRNAs can potentially produce synergistic biological effects via cooperative and/or redundant mechanisms of gene expression control (i.e. by targeting the same or different genes within the same pathway) [6], we speculated that the largely distinct sets of altered miRNAs associated with prior PE (cohort 1 and 2), current ACS (cohort 3) and current PE (cohort 4) exposure might nevertheless be involved in the regulation of common underlying biological pathways. To address this possibility, differentially altered miRNAs were combined with their predicted gene targets (via TargetScan 7.2 database), and pathway enrichment analysis was performed to identify pathways in the Kyoto Encyclopedia of Genes and Genomes (KEGG) database that were significantly enriched with these gene targets (Figure 4A). From a total of 244 unique pathways, 36 and 33 of these pathways were found to be significantly enriched (FDR < 0.05) with genes targeted by miRNAs associated with prior PE exposure in the ACS and non-ACS cohorts, respectively (Figure 4B). Seventeen pathways were significantly enriched (FDR < 0.05) in relation to current ACS exposure (i.e. cohort 3), and 29 pathways were significantly overrepresented in relation to current PE exposure (cohort 4) (Figure 4B). The majority of pathways associated with a history of PE (i.e. 28 pathways or 78–85% of pathways associated with cohort 1 and 2, respectively) were common between the ACS and non-ACS cohorts, and 12 of these pathways were also implicated in current ACS exposure and current PE exposure, further suggesting the importance of these pathways in the relationship between PE and CVD (Figure 4C). The identities and statistical significance of all biological pathways identified in the pathway enrichment analysis of predicted gene targets are shown in Supplementary Figure S5. Wnt signaling was the most significantly enriched pathway across all four exposure comparisons (based on the mean of the FDR-adjusted *P*-values; Figure 4C). To further prioritize targets, pathway enrichment analysis was also performed after combining the differentially altered miRNAs with a smaller database of experimentally validated gene targets (i.e. miRTarBase7.0 database). In this analysis, a history of preeclampsia in the ACS and non-ACS cohorts was associated with 20 and 33 significantly enriched pathways (FDR < 0.05), respectively, of which the majority (i.e. 17 pathways) were shared between both cohorts (Figure 4D). Four and two KEGG pathways were significantly enriched (FDR < 0.05) with experimentally validated targets of miRNAs related to current ACS and current PE exposure, respectively. Wnt signaling was the only common pathway between all four exposure groups (Figure 4E). Supplementary Figure S6 shows the identities and statistical significance of all pathways identified using the validated gene targets from miRTarBase.

### Characterization of miRNA–target gene interactions in Wnt signaling associated with PE and ACS

**Figure 5:**
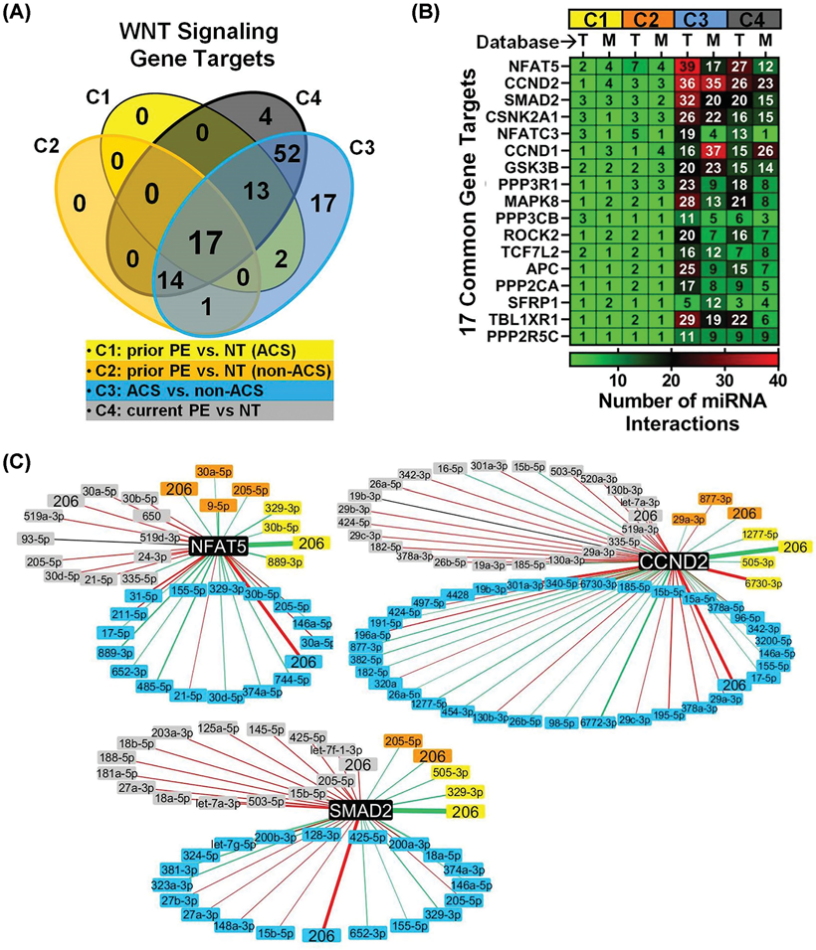
Characterization of miRNA–target gene interactions in Wnt signaling associated with PE and ACS (**A**) Venn diagram showing the number of miRNA target genes related to Wnt signaling that are shared between different exposure cohorts (C1-C4). Only genes that were identified in both TargetScan (T) and miRTarBase (M) databases were included. (**B**) 17 target genes implicated in Wnt signaling in all 4 exposure cohorts. Heatmap shows the number of miRNAs that target each gene, stratified by database and cohort. (**C**) MiRNA interactions with three highly networked genes (NFAT5, CCND2 and SMAD2) identified in miRTarBase. Nodes represent differentially altered miRNAs color-coded according to cohort and related target genes (black nodes). Red and green lines denote increased and decreased miRNA plasma levels, respectively. Line thickness is scaled to the magnitude of fold change in miRNA level.

Since Wnt signaling was the most significantly altered pathway common to both preeclampsia and ACS, we sought to define the network of potential interactions between the altered miRNAs and their predicted/validated gene targets in this pathway. Across all 4 cohorts, there were a total of 2557 and 1676 interactions between the altered miRNAs and their predicted (Targetscan7.2) or experimentally validated (miRTarBase7.0) gene targets, respectively (Figure S7). To further prioritize these miRNA target interactions we focused on overlapping gene targets that were identified in both Targetscan and miRTarBase (Supplementary Table S8) and then identified a further subset of 17 genes that were implicated in all 4 cohorts (Figure 5A). Many of these genes were targeted by multiple miRNAs within the same cohort (Figure 5B) that also showed the same direction of regulation (Figure 5C), suggesting considerable redundancy and/or cooperation in the regulation of Wnt signaling in the cardiovascular sequelae of PE. Among a number of potential effector genes that may be highly regulated in this context were Nuclear Factor of Activated T-cells 5 (NFAT5), Cyclin D2 (CCND2) and Mothers Against Decapentaplegic homolog 2 (SMAD2), which were the most targeted genes showing an average of three different miRNA interactions per gene in cohorts 1 and 2 in relation to a history of PE, 20 miRNA interactions per gene in relation to current PE exposure (cohort 4), and 30 miRNA interactions per gene in relation to ACS exposure (cohort 3) (Figure 5B). The network of experimentally validated (miRTarBase) miRNA interactions with NFAT5, CCND2 and SMAD2 for each cohort is presented in Figure 5C and predicted (Targetscan) miRNA interactions with these genes are presented in Supplementary Figure S8. Of note, each of these genes is a target of miR-206, which was the only miRNA altered in all 4 cohorts.

## Discussion

In the present study, we sought to identify miRNAs and biological pathways that may contribute to the long-term cardiovascular sequelae of PE. Our results showed that women with a history of PE exhibit marked perturbations in the circulating levels of miR-206 and other miRNAs, which were also shown to be dysregulated in relation to acute coronary syndrome. These miRNAs are implicated in the regulation of several different KEGG biological pathways, most notably Wnt-signaling, which is known to play important roles in vascular and cardiac function.

Our key finding that circulating levels of miR-206 were differentially altered in women with a history of (or current) PE and in relation to an acute ischemic event suggests that miR-206 may represent an important link between PE and the increased risk of cardiovascular complications that has been reported in many epidemiological studies [1,16]. In women with ACS, a history of PE was associated with ∼10-fold lower plasma levels of miR-206 compared with a history of NT pregnancy, and this was corroborated in a second cohort of women without ACS, though the magnitude of change was more modest at 1.8-fold. If decreased circulating levels of miRNAs reflect underlying changes in cell/tissue expression, a possibility that has been demonstrated elsewhere [17], a number of previous studies suggest how lower miR-206 levels might exert atherogenic effects through distinct cell-type specific mechanisms to elevate the risk of cardiovascular disease. MiR-206 has been shown to be a negative regulator of the Liver X receptor α (LXRα) gene, which serves as a major regulator of lipid homeostasis in the liver by controlling the expression of lipogenic genes [18]. Therefore, the decreased levels of miR-206 could be speculated to facilitate the expression of lipogenic genes that may promote plague formation. Decreased levels of miR-206 have also been associated with increased risk of atherosclerosis via mechanisms involving vascular smooth muscle cells (VSMC). Tao et al. reported decreased VSMC proliferation in cell cultures supplemented with miR-206 mimics, which suggests that a reduction in miR-206 levels might have the opposite effect and contribute to aberrant VSMC proliferation [19]. The authors also reported reduced expression of miR-206 in human atherosclerosis tissue samples [19]. In a separate study, miR-206 was shown to be reduced in the renal artery of rats with hyperlipidemia, which was associated with increased vascular hyperreactivity [20], and the antisense-mediated down-regulation of miR-206 was shown to increase the contractile response of VSMCs in cell culture, consistent with a pro-atherogenic effect [20]. Another potential mechanism by which decreased miR-206 levels may contribute to the progression of atherosclerosis is through regulation of macrophage function. Xu et al. reported that overexpression of miR-206 inhibited oxidative stress, while lncRNA-mediated reduction of miR-206 via ‘sponging’ reversed this effect and exacerbated atherosclerosis events induced by oxidized low-density lipoprotein (oxLDL) in human monocytic/macrophage cells [21]. In yet another study, Vinod et al. showed that the experimental down-regulation of miR-206 decreased cholesterol efflux in mouse peritoneal macrophages [22]. The impairment of cholesterol release from macrophages might facilitate the development of foam cells and a pro-thrombotic state that precipitates future ischemic events.

The plasma level of miR-206 has also previously been shown to be altered in women at 28 weeks of gestation who later developed PE (compared with women who experienced a NT pregnancy) [23]. Interestingly, however, these women showed a small 1.4-fold increase in miR-206 plasma levels, which mirrored a 1.4-fold increase in placental tissue that was collected at delivery from another cohort of women [23]. Therefore, we speculate that this previously reported elevation in miR-206 plasma level induced near the time of PE may be a transient effect driven by the increased expression and secretion from the placenta. Of note, we generally observed minimal overlap in the differentially altered miRNAs identified in the present study (in relation to prior PE exposure) as compared with miRNAs previously reported to be altered at the time of PE. This suggests that the majority of miRNA alterations associated with a history of PE in the current study may reflect biological changes that occurred after the resolution of PE.

In the present study, plasma levels of miR-206 were also found to be altered in women with ACS (compared with non-ACS controls); however, levels were elevated with ACS exposure in contrast with the decreased levels observed in women with a history of PE. Since levels of circulating miRNAs may potentially be impacted by changes in release and uptake from many different tissues, we speculate that the observed difference in the direction of change of miR-206 may reflect distinct cell-type specific responses to underlying disease activity. For instance, miR-206 expression has been reported to be up-regulated in the heart tissue of rats with myocardial infarction or diabetic myocardial injury [24,25]. Therefore, marked changes in the myocardium at the time of ACS could potentially mask the vascular and metabolic abnormalities associated with PE described earlier. Of note, several other miRNAs that belong to the myomiR family of miRNAs (i.e. that are abundantly expressed in cardiac and skeletal muscle) were also found to be significantly elevated in plasma in the present study in relation to ACS including miR-499a-5p, miR-1 and miR-133a-3p, which provides support for this theory.

Another key finding of the present study is the identification of Wnt signaling as a potentially important pathway linking PE to increased risk of ACS. This is supported by pathway enrichment analyses conducted with two databases comprising both the predicted and experimentally validated gene targets of the differentially altered miRNA candidates. In addition to providing insights into downstream effector genes that may be regulated by the altered miRNAs, pathway enrichment analysis can offer further insight into shared mechanisms that might not otherwise be discernible from miRNA expression profiles alone. For instance, while only 13–20% of differentially altered miRNAs were common between cohorts 1 and 2 (in relation to prior PE exposure), 78–85% of the biological pathways they are predicted to regulate were shared.

Wnt signaling was the top pathway among 12 pathways that were significantly enriched in the predicted gene targets of miRNAs that were altered in subjects with prior and current PE exposure and ACS, and the only pathway that was also confirmed using the miRTarBase database of experimentally validated gene targets. Of note, a number of prior clinical and preclinical studies have implicated Wnt signaling in both vascular (e.g. atherosclerosis, endothelial dysfunction and vascular calcification) and cardiac disease (e.g. myocardial infarction and heart failure) [26], supporting the relevance of this pathway to pathophysiological activity in both PE and CVD. We also identified potential gene targets that may be regulated by the differentially altered miRNAs, and are also known to have atherogenic activities. In particular, NFAT5, CCND2 and SMAD2 were among the most highly targeted genes with connections to multiple miRNAs (including miR-206), which suggests an increased likelihood for regulation. The transcription factor, NFAT5, has been shown to be up-regulated in relation to atherosclerosis and neointimal hyperplasia [27], consistent with a negative regulatory relationship with miR-206 (which was decreased in women with a history of PE in the present study). Another study reported that up-regulation of NFAT5 may increase the migratory and proliferative activity of VSMCs and promote maladaptive arterial stiffening [28]. CCND2, a cell cycle regulator, has also been implicated in the pathobiology of cardiovascular complications related to diabetes [29,30], with increased levels related to increased endothelial cell proliferation [29]. SMAD2 expression has been reported to be up-regulated in human macrophages from atherosclerotic lesions [31], and to negatively regulate inducible nitric oxide synthase (iNOS) expression in macrophages [32].

Because PE and CVD share several common risk factors and pathophysiological features, a critical question is whether the occurrence of PE merely unmasks this existing risk or contributes directly to future CVD. We attempted to provide insight into this question by investigating prior PE exposure in the context of two cohorts with either a high (cohort 1) or significantly lower (cohort 2) burden of cardiovascular risk factors. While not conclusive, the fact that miR-206 and Wnt signaling were identified in both cohorts suggests these biological features may be affected by PE independent of existing CVD risk factors, and is consistent with the possibility of a causal role in future CVD. Evidence of a possible causal relationship between PE and future CVD has previously been demonstrated in a mouse model of experimentally induced PE with no pre-existing CVD risk factors [33]. Pruthi et al. reported that the blood vessels of exposed mice exhibited an enhanced vascular remodelling response to future injury, which persisted after pregnancy and resolution of PE [33]. While the authors showed the enhanced vascular response in exposed mice could be attributed to an increase in smooth muscle cell (SMC) proliferation and vessel fibrosis, they did not explore specific molecular mechanisms in further detail. However, our finding that miR-206 and Wnt signaling (known to contribute to the regulation of vascular SMC proliferation and/or fibrosis [26]) may be central features linking women with prior PE and future CVD is consistent with and provides a new translational link for these prior experimental findings.

The present study has several strengths that distinguish it from previous studies. To the best of our knowledge, this is the first application of next-generation sequencing to assess circulating miRNAs in women with a history of PE, which in contrast with our previous report that screened only 372 miRNAs by PCR array [11], now provides a comprehensive assessment of all 2578 mature human miRNAs annotated in miRBase version 20. The evaluation of two independent cohorts of women with different cardiovascular backgrounds is another strength of the present study and improvement over our prior study [11], which provides evidence of the generalizability of key findings. Another potential strength of the present study is the relatively large sample sizes used for high-throughput miRNA screening (i.e. 17–20/group). By comparison, the majority of previous high-throughput screening studies that assessed circulating miRNA levels in women during pregnancies complicated by PE used sample sizes of less than 10 subjects per group [10], and two prior RNA-sequencing-based studies employed markedly smaller sample sizes (i.e. ≤4 subjects/group) [34,35]. Nevertheless, we also wish to note that while the present study was robustly powered (∼80%) to detect >2.5-fold changes in levels of the majority of miRNAs, the detection of smaller effect sizes would be relatively underpowered and susceptible to false negative errors. This may explain why small alterations (i.e. <2-fold) in several atherogenic miRNAs previously linked to a history of PE (i.e. miR-126-3p, miR-122-5p and miR-146a-5p) [11] were not reproduced in the present study, in addition to other potential factors including differences in methodology and patient heterogeneity.

One key limitation of the present study is the retrospective cross-sectional observational design that limits conclusions about causality, and whether altered miRNAs represent potential mediators or just markers of underlying cardiovascular abnormalities. Although prospective and other clinical study designs with higher evidentiary value would clearly be beneficial, the long latency period of ∼20 years between exposure and outcome in this context would be highly impractical to implement. In addition, the identification of common miRNAs and signaling pathways at the intersection of several independent cohorts of women comprising past and current PE and ACS exposures is suggestive, consistent with the known mechanisms by which different sets of miRNAs can regulate overlapping gene networks, and supports the overall biological plausibility of our findings. While it is beyond the scope of the current observational study, our results should help to inform the design of *in vitro* and/or *in vivo* preclinical experiments to further interrogate potential causal mechanisms related to specific miRNA and gene targets.

The present study provides the most comprehensive assessment of circulating miRNAs in women with a history of PE. The biological implications of altered miRNA levels related to a history of PE were interpreted through gene target integration and pathway enrichment analysis in two distinct cohorts of women with and without ACS, and provided novel evidence that miR-206 and Wnt signaling may play important roles in the relationship between PE and future CVD. This helps to address a marked knowledge gap on potential mechanisms underlying the long-term cardiovascular effects of PE, and should help to inform the design of further experimental studies to delineate whether PE is a marker or mediator of CVD. This may lead to improved identification of high risk women who may benefit from closer monitoring and aggressive management of CVD risk factors.

## Clinical perspectives

- Epidemiological studies have shown that women with a history of preeclampsia have a higher risk of cardiovascular disease in later life, yet the molecular determinants that contribute to this risk remain poorly defined.
- RNA-sequencing and pathway enrichment analysis showed that alterations in circulating miR-206 and Wnt signaling were associated with acute cardiovascular complications and a history of preeclampsia.
- The present study provides novel insights into miRNAs, target genes and biological pathways that may underlie the long term cardiovascular sequelae of preeclampsia.

## Supplementary Material

Supplementary Figure S1-S7 and Tables S1-S8

## Abbreviations

ACS: acute coronary syndrome
CVD: cardiovascular disease
oxLDL: oxidized low-density lipoprotein
PE: preeclampsia
SMC: smooth muscle cell
VSMC: vascular smooth muscle cell
