# Supplementary material for: Circulating miR-206 and Wnt-signaling are associated with cardiovascular complications and a history of preeclampsia in women
Source: Clin Sci (Lond). 2020 Jan 21;134(2):87–101. doi: 10.1042/CS20190920 (PMC8299351; doi:10.1042/CS20190920)
Supplement: Supplementary Figure S1-S7 and Tables S1-S8 [file CS-2019-0920_supp.pdf]

Online Data Supplement for

Circulating miR-206 and Wnt-signaling are associated with cardiovascular complications and a history of preeclampsia in women

Running Title: Circulating miRNAs in prior preeclampsia

Kenny SCHLOSSER[1], Amanpreet KAUR[2], Natalie DAYAN[3], Duncan J STEWART*[1,4], Louise PILOTE* [2,3], Christian DELLES*[5]

denotes co-senior authors

1. Sinclair Centre for Regenerative Medicine, Ottawa Hospital Research Institute, Ottawa, Ontario, Canada
2. Research Institute, McGill University Health Centre, Montreal, Quebec, Canada
3. Department of Medicine, Division of General Internal Medicine, McGill University Health Centre, Montreal, Quebec, Canada
4. Department of Cellular and Molecular Medicine, University of Ottawa, Ottawa, Ontario Canada
5. Institute of Cardiovascular and Medical Sciences, University of Glasgow, Scotland, UK

Corresponding author information

Dr. Christian Delles
Institute of Cardiovascular and Medical Sciences, University of Glasgow
126 University Place, Glasgow, G12 8TA, UK
christian.delles@glasgow.ac.uk

Supplementary Methods

Cohort 1: ACS subjects with a history of PE or NT pregnancy

Women with acute coronary syndrome (ACS) and a history of preeclampsia (PE) or normotensive (NT) pregnancy were identified from the GENESIS-PRAXY multicentre cohort study of adults (aged ≤ 55 years) hospitalized with ACS. Detailed methods have been previously described [1]. Participants were recruited between January 2009 and April 2013 from 24 centers across Canada, one in the US and one in Switzerland. All participating sites received ethics approval from their respective ethics review boards, and participants provided written informed consent. Eligible participants were adults aged 18-55 years diagnosed with ACS, and able to provide informed consent. Pregnancy data was collected by detailed self-reported questionnaires of all female participants at study entry. Women were classified as prior PE if they reported either PE or high blood pressure in addition to proteinuria. Women who were unsure about the presence or absence of a pregnancy complication or for whom completion of these questions was incomplete were excluded. The time since last pregnancy was estimated using the age of the youngest biological child, to serve as a proxy for the interval between pregnancy and incident ACS. Venipuncture was performed on all consenting participants within 24 hours of hospital admission for ACS. Whole blood collected in citrate Vacutainers was spun at 4 ˚C at 3000 rpm for 10 minutes and the plasma supernatant removed and frozen at -80 ˚C. A total of 40 subjects were initially selected for miRNA sequencing (n=20 subjects/exposure group) after matching for cardiovascular disease risk factors including hypertension, diabetes, smoking and age. The final sample size was reduced to n=17-18 subjects/exposure group after excluding plasma specimens that failed RNA- and/or library-quality control tests prior to sequencing (further details provided under quality control assays).

Cohort 2: non-ACS subjects with a history of PE or NT pregnancy

Women without ACS and a history of either preeclampsia or normotensive pregnancy were identified from The Cardiovascular Consequences of Pre-eclampsia (COPS) study at the British Heart Foundation Glasgow Cardiovascular Research Centre (BHF GCRC). Women were recruited from multiple sources including the previous Generation Scotland: Scottish Family Health Study [2], the Proteomics in Pre-eclampsia study [3], patients who attended blood pressure clinics and friends and colleagues of participants who contacted us with interest in participating. The study was approved by the West of Scotland Research Ethics Committee 3 (Reference 12/WS/0306), and participants provided written informed consent. The index pregnancy was defined as the first pregnancy in normotensive women and the first pre-eclamptic pregnancy in those with pre-eclampsia. Women were excluded if they were >60 years old, already had established cardiovascular disease or if they were unable to give informed consent. Participants completed a questionnaire asking for obstetric history, past medical history, drug history, smoking history and family history. Blood samples were taken from the antecubital fossa using a standard tourniquet and Vacutainer system, and centrifuged at 4 °C at 2500 rpm for 15 minutes and plasma supernatant removed and frozen -80 °C. A total of 40 subjects (n=20 subjects/exposure group) matched on hypertension, diabetes, and age were selected for miRNA sequencing. All specimens passed pre-sequencing quality control tests.

Cohort 3: ACS and non-ACS subjects

The comparison of all women in cohort 1 (n=35 total ACS subjects) versus cohort 2 (n=40 total non-ACS subjects) was used to assess the impact of ACS on circulating miRNA levels.

Cohort 4: Women with PE or NT pregnancy
Information for this cohort was derived from six prior independent studies of preeclamptic women (with no ACS) reported in the systematic review by Sheikh et al. [4]. A total of 104 circulating miRNAs were identified via high-throughput screening methods as differentially altered in plasma, serum or whole blood between women with preeclampsia versus normotensive pregnancy.

RNA isolation
Total RNA including small RNAs <200 nt in size were purified from a fixed volume of 200 µL of citrate-plasma using the miRNeasy Serum/Plasma kit (i.e., phenol/guanidine-based sample lysis and silica-column-based purification) according to manufacturer instructions (Qiagen Genomic Services; cat #217184). Cel-miR-39 was added as a spike-in control during RNA purification for downstream quality control assessment. RNA was eluted with 14 µL of RNase-free water.

RNA Quality Control
Because the concentration of eluted total RNA purified from plasma is typically too low to assess RNA purity and integrity by standard methods involving UV absorbance ratios and Agilent Bioanalyzer, the quality of the extracted total RNA was assessed using a miScript miRNA QC PCR array (Qiagen Genomic Services; cat #MIHS-989ZE). The array contains primer assays for several types of external spike-in controls to assess variations during RNA extraction (i.e. cel-miR-39), an indicator to monitor relative efficiency and potential inhibition of the reverse transcription reaction (i.e., miRTC) and an indicator to monitor relative efficiency and potential inhibition of the downstream PCR reaction (i.e., PPC). In addition, primer assays for several endogenous miRNAs (i.e., miR-16, miR-21 and miR-191) are included as positive controls since these miRNAs are ubiquitously expressed across many different biologic specimens including body fluids, and several endogenous small nuclear/nucleolar RNAs (i.e., SNORD61, SNORD95, and SNORD96A) are included as negative controls (or markers of cellular contamination) since these are typically expressed abundantly in cells, but poorly in body fluids. During this quality assessment, specimens from 4 subjects in cohort 1 (including 1 prior PE subject and 3 prior normotensive pregnancy subjects) showed evidence of poor RNA quality (Figure S1), and therefore were not sequenced after the corresponding sequencing libraries showed poor yields (noted below; Figure S2). One additional sample from cohort 1 (a subject with prior PE) was excluded prior to sequencing because the qPCR assay showed evidence of cellular contamination (Figure S1). Overall, the results of the quality control assays suggested that the quality and quantity of extracted RNA was generally comparable between samples, with the above noted exceptions.

Library Construction, Quality Control and Sequencing
A fixed volume of 5 µL of the extracted total RNA was used for NGS library construction using the QIAseq miRNA library kit according to manufacturer instructions (Qiagen Genomic Services; cat #331505). In brief, specifically designed 3' and 5' adapters were sequentially ligated to mature miRNAs. The ligated miRNAs were then reverse-transcribed to generate cDNA using a reverse-transcription (RT) primer with a unique molecular index (UMI) tag. Of note, the kit is designed to minimize adapter dimers and hy4 Y RNA contamination during cDNA cleanup. The

cDNA library was then amplified via PCR (21 cycles) and subsequently purified prior to quality control assessment. Library qualification was conducted with High Sensitivity DNA chips on an Agilent Bioanalyzer 2100 instrument (or Agilent TapeStation 4200) to confirm proper library size and yield (Figure S2). In addition, library concentrations were quantified on a Qubit device using the Quant-iT dsDNA High Sensitivity Assay Kit. Libraries were pooled in equimolar ratios and quantified using qPCR. Library pools were then sequenced on an Illumina NextSeq 500 sequencer using NextSeq 500 Mid Output Reagent Cartridge v2 (75 cycles). Raw data was de-multiplexed and FASTQ data files for each sample were generated using the bcl2fastq software (Illumina Inc.), and checked for quality using the FastQC tool.

Sequence Trimming, UMI consolidation and read mapping
Trimming of library and sequencing adaptors was performed after sequencing with Cutadapt (1.11), and reads were analyzed for the presence of the unique molecular index (UMI) tags that were added during library construction. On average, 22.2 million raw reads were obtained per sample, and 10.2 million reads per sample remained after excluding reads that were missing adaptors, too short (insert sequence <16 nt) or did not contain the minimal length UMI tag (i.e., 10 nt). All reads containing identical insert sequence and UMI sequence (insert-UMI pair) were collapsed into a single read (to correct for potential library amplification bias and improve miRNA quantification) and passed into the analysis pipeline. On average, 2.3 million UMI-corrected reads were obtained per sample. Bowtie2 (2.2.2) was used for mapping reads based on the criterion of having a perfect sequence match to the reference sequence. Reads were aligned to miRBase 20 and/or human GRCh37 reference genome. Conversion of raw reads to UMI-corrected mapped counts was conducted by Qiagen Genomic Services.

miRNA-gene target integration and Pathway Enrichment Analysis
miRNA-gene target integration and pathway enrichment analysis were performed in Partek Genomics Suite using default settings in the miRNA integration and biological interpretation pathway analysis features. Differentially-altered miRNA candidates were combined with predicted gene targets using Targetscan 7.2 [5] (conserved miRNA sites database; 1,468,778 records) or experimentally-validated gene targets using miRTarBase 7.0 (422 517 curated miRNA-target interactions) [6], and pathway enrichment determined with a Fisher's exact test.

Pre and post study sample size and power estimations
Study sample sizes of n=20/group were estimated *a priori* to achieve 80% power to detect a >2-fold change in miRNA level with a bonferonni-adjusted alpha value of 0.0001 assuming 500 detectable miRNAs for differential analysis and a coefficient of variation (CV) of 0.47. This variation in miRNA levels was estimated using the median CV from 235 miRs previously measured by (RT)-qPCR array in a similar set of human plasma samples [7], and extrapolated into a common standard deviation assuming mean miR levels between 10-20 counts. These initial sample size calculations were performed with the online calculator at https://www.stat.ubc.ca/~rollin/stats/ssize/n2.html. Post hoc estimations of the dispersion in miRNA count levels from the completed sequencing experiment, and sample size-power relationships were calculated using the RNASeqSampleSize software package in R language and online interface at http://cqs.mc.vanderbilt.edu/shiny/RnaSeqSampleSize/.[8]

Statistical Analysis

All statistical tests comparing cohort characteristics were performed in Graphpad Prism 8.0. Data normality was assessed using the D'Agostino Pearson test. Differences between exposure groups for continuous data was assessed using a Mann-Whitney or unpaired t-test, depending on data normality as appropriate. Differences in categorical variables were assessed via Fisher's exact test. Data are presented as mean ± standard deviation (SD) unless otherwise specified. Differential expression analysis was conducted on the subset of samples related to the specific groups being compared, using UMI-corrected miRNA counts as input into the EdgeR statistical software package (Bioconductor, http://www.bioconductor.org/). Data was preprocessed to exclude poorly detectable miRNAs such that the sum of the counts per million mapped reads (CPM) for each miRNA in all samples pertaining to the comparison subset were > 10. The filtered data was normalized using the trimmed mean of M-values (TMM) normalization method in EdgeR to compensate for sample specific effects related to variations in sequencing depth and RNA composition. MiRNA levels in some figures are presented simply as counts per million mapped reads (CPM), which only corrects for differences in sequencing depth between samples. P-values and Benjamini-Hochberg false discovery rate (FDR)-correct p values for differentially altered miRNAs were calculated with an exact test assuming a negative binomial distribution in EdgeR. Principal component analysis and unsupervised hierarchical clustering and heatmap construction was performed with default parameters in Partek Genomics Suite 7.2 using log2 transformed TMM-normalized miRNA counts (with offset 1 to account for 0 values).

Table S1. Differential expression analysis of 427 miRNAs detected in plasma from women with acute coronary syndrome (cohort 1) and a history of preeclampsia (PE, n=18) versus normotensive (NT, n=17) pregnancy. MiRNA levels are expressed as mean counts per million mapped reads (CPM). MiRNAs are listed in descending order of statistical significance.

miRNA	Fold Change (PE/NT)	p value	FDR-adjusted p value	miR level (CPM)
miR-206	-10.6	1.64E-06	6.98E-04	242
miR-1292-5p	-3.6	8.28E-05	1.77E-02	8
miR-184	10.3	2.35E-04	3.35E-02	52
miR-376a-3p	-4.7	1.10E-03	1.17E-01	7
miR-499a-5p	5.8	1.85E-03	1.58E-01	45
miR-218-5p	5.4	2.24E-03	1.60E-01	7
miR-6730-3p	7.3	3.46E-03	1.96E-01	4
miR-1299	4.9	3.67E-03	1.96E-01	23
miR-1	3.0	5.46E-03	2.59E-01	531
miR-889-3p	-2.9	7.52E-03	3.21E-01	17
miR-30b-5p	-1.8	1.23E-02	4.45E-01	15
miR-4662a-5p	3.5	1.33E-02	4.45E-01	5
miR-136-3p	-2.7	1.35E-02	4.45E-01	10
miR-431-5p	-2.4	1.47E-02	4.49E-01	73
miR-874-5p	3.3	1.64E-02	4.54E-01	4
miR-28-5p	-2.7	1.70E-02	4.54E-01	5
miR-6767-5p	2.7	1.95E-02	4.91E-01	7
miR-505-3p	-1.9	2.22E-02	5.01E-01	9
miR-493-5p	-2.3	2.29E-02	5.01E-01	13
miR-1277-5p	-1.8	2.35E-02	5.01E-01	13
miR-369-5p	-2.2	2.54E-02	5.07E-01	18
miR-6741-3p	1.7	2.61E-02	5.07E-01	11
miR-133a-3p	2.7	2.78E-02	5.16E-01	76
miR-221-5p	-1.9	3.03E-02	5.39E-01	9
miR-2355-3p	-1.9	3.54E-02	5.91E-01	7
miR-4667-5p	3.6	3.60E-02	5.91E-01	4
miR-3591-5p	3.8	4.01E-02	6.34E-01	5
miR-769-5p	-1.5	4.20E-02	6.40E-01	27
miR-329-3p	-2.5	4.56E-02	6.54E-01	7
miR-202-3p	2.9	4.68E-02	6.54E-01	6
miR-130b-3p	1.5	5.16E-02	6.54E-01	47
miR-27a-5p	2.0	5.32E-02	6.54E-01	5
miR-335-3p	-1.9	5.48E-02	6.54E-01	15

**miR-195-5p**	1.5	5.51E-02	6.54E-01	99
**miR-378c**	1.6	5.79E-02	6.54E-01	15
**miR-5189-3p**	2.3	5.89E-02	6.54E-01	6
**miR-378a-3p**	1.4	6.01E-02	6.54E-01	465
**miR-150-3p**	1.6	6.19E-02	6.54E-01	21
**miR-493-3p**	-2.1	6.62E-02	6.54E-01	9
**miR-128-3p**	-1.3	6.67E-02	6.54E-01	324
**miR-29c-3p**	1.3	6.82E-02	6.54E-01	1241
**miR-339-5p**	-1.4	6.95E-02	6.54E-01	117
**miR-10b-3p**	2.0	7.25E-02	6.54E-01	9
**miR-432-5p**	-1.8	7.28E-02	6.54E-01	434
**miR-628-3p**	-1.4	7.42E-02	6.54E-01	61
**miR-223-3p**	-1.5	7.50E-02	6.54E-01	5871
**miR-369-3p**	-1.8	7.55E-02	6.54E-01	19
**miR-6859-5p**	2.0	7.63E-02	6.54E-01	5
**miR-423-3p**	-1.4	7.72E-02	6.54E-01	553
**miR-4775**	-2.4	7.99E-02	6.54E-01	4
**miR-381-3p**	-2.0	8.08E-02	6.54E-01	34
**miR-548a-3p**	-2.0	8.14E-02	6.54E-01	6
**miR-335-5p**	1.5	8.34E-02	6.54E-01	397
**miR-3127-5p**	1.9	8.73E-02	6.54E-01	8
**miR-382-5p**	-1.8	9.17E-02	6.54E-01	262
**miR-5187-5p**	-1.8	9.32E-02	6.54E-01	8
**miR-3688-3p**	-1.5	9.64E-02	6.54E-01	14
**miR-181c-3p**	-2.0	9.78E-02	6.54E-01	7
**miR-4714-3p**	2.4	9.79E-02	6.54E-01	5
**miR-9-5p**	-2.4	9.89E-02	6.54E-01	12
**miR-26b-3p**	1.7	9.90E-02	6.54E-01	9
**miR-4433b-5p**	-1.7	1.00E-01	6.54E-01	955
**miR-205-5p**	-1.5	1.03E-01	6.54E-01	90
**miR-1250-5p**	2.6	1.03E-01	6.54E-01	4
**miR-491-5p**	-1.7	1.05E-01	6.54E-01	12
**miR-18a-3p**	-1.4	1.07E-01	6.54E-01	39
**miR-143-5p**	1.7	1.09E-01	6.54E-01	16
**miR-664b-5p**	1.5	1.09E-01	6.54E-01	12
**miR-654-3p**	-1.8	1.11E-01	6.54E-01	42
**miR-208b-3p**	2.5	1.12E-01	6.54E-01	88
**miR-30d-5p**	-1.2	1.12E-01	6.54E-01	12944
**miR-376c-3p**	-2.2	1.14E-01	6.54E-01	7
**miR-4669**	-2.3	1.15E-01	6.54E-01	9
**miR-1304-3p**	-1.5	1.15E-01	6.54E-01	14

**miR-652-3p**	-1.4	1.16E-01	6.54E-01	36
**miR-125b-1-3p**	2.1	1.17E-01	6.54E-01	8
**miR-574-3p**	1.5	1.19E-01	6.54E-01	74
**miR-3679-5p**	1.9	1.20E-01	6.54E-01	8
**miR-3613-3p**	-1.8	1.21E-01	6.54E-01	6
**miR-323a-3p**	-1.7	1.26E-01	6.56E-01	21
**miR-379-5p**	-1.7	1.26E-01	6.56E-01	53
**miR-885-5p**	-1.8	1.28E-01	6.56E-01	18
**miR-361-3p**	-1.3	1.28E-01	6.56E-01	351
**miR-145-3p**	2.2	1.31E-01	6.56E-01	6
**miR-127-3p**	-1.9	1.34E-01	6.56E-01	10
**miR-4738-3p**	1.9	1.36E-01	6.56E-01	6
**miR-6721-5p**	-1.7	1.36E-01	6.56E-01	7
**miR-3168**	1.6	1.38E-01	6.56E-01	36
**miR-1237-3p**	-1.7	1.38E-01	6.56E-01	7
**miR-296-5p**	1.4	1.38E-01	6.56E-01	22
**miR-452-5p**	1.8	1.44E-01	6.69E-01	12
**miR-338-3p**	-1.5	1.45E-01	6.69E-01	17
**miR-576-5p**	-1.3	1.46E-01	6.69E-01	171
**miR-1301-3p**	-1.5	1.47E-01	6.69E-01	26
**miR-339-3p**	-1.3	1.57E-01	6.97E-01	38
**miR-1307-3p**	-1.3	1.62E-01	6.97E-01	734
**miR-17-5p**	-1.3	1.64E-01	6.97E-01	117
**let-7d-5p**	1.2	1.64E-01	6.97E-01	1828
**miR-425-5p**	-1.2	1.64E-01	6.97E-01	4293
**miR-23a-5p**	1.9	1.65E-01	6.97E-01	4
**miR-27a-3p**	1.2	1.66E-01	6.97E-01	201
**miR-1273h-3p**	-1.6	1.67E-01	6.97E-01	10
**miR-196a-5p**	-1.7	1.75E-01	7.14E-01	16
**miR-151a-3p**	-1.3	1.76E-01	7.14E-01	2003
**miR-548n**	-1.7	1.76E-01	7.14E-01	6
**miR-106b-5p**	-1.4	1.77E-01	7.14E-01	25
**miR-4732-3p**	-1.3	1.84E-01	7.21E-01	208
**miR-29b-3p**	1.2	1.85E-01	7.21E-01	152
**miR-126-3p**	-1.2	1.88E-01	7.21E-01	9787
**miR-93-3p**	-1.3	1.88E-01	7.21E-01	32
**miR-30a-3p**	1.3	1.92E-01	7.21E-01	58
**miR-323b-3p**	-1.6	1.93E-01	7.21E-01	31
**miR-382-3p**	-1.7	1.94E-01	7.21E-01	13
**miR-1255b-5p**	-1.3	1.94E-01	7.21E-01	45
**miR-95-3p**	1.5	1.96E-01	7.21E-01	18

**miR-425-3p**	-1.2	1.97E-01	7.21E-01	75
**miR-409-3p**	-1.6	1.98E-01	7.21E-01	346
**miR-208a-3p**	2.1	1.99E-01	7.21E-01	5
**miR-22-5p**	-1.5	2.01E-01	7.23E-01	10
**miR-199a-5p**	-1.5	2.07E-01	7.31E-01	17
**miR-30a-5p**	1.3	2.07E-01	7.31E-01	1276
**miR-485-5p**	-1.5	2.13E-01	7.41E-01	41
**miR-144-5p**	-1.3	2.13E-01	7.41E-01	197
**miR-30d-3p**	1.7	2.16E-01	7.41E-01	6
**miR-215-5p**	1.6	2.19E-01	7.41E-01	82
**miR-411-5p**	-1.6	2.20E-01	7.41E-01	13
**miR-222-3p**	1.2	2.20E-01	7.41E-01	113
**miR-129-5p**	-1.5	2.26E-01	7.52E-01	7
**miR-132-3p**	1.3	2.27E-01	7.52E-01	46
**miR-671-5p**	-1.3	2.32E-01	7.59E-01	57
**miR-10b-5p**	1.3	2.33E-01	7.59E-01	1401
**miR-29a-3p**	1.2	2.36E-01	7.59E-01	1092
**miR-1226-3p**	-1.3	2.38E-01	7.59E-01	10
**miR-548l**	-1.3	2.40E-01	7.59E-01	9
**miR-485-3p**	-1.6	2.41E-01	7.59E-01	70
**miR-7976**	-1.4	2.42E-01	7.59E-01	9
**miR-4710**	2.1	2.47E-01	7.69E-01	4
**miR-15a-5p**	1.2	2.58E-01	7.98E-01	599
**miR-21-3p**	-1.3	2.61E-01	8.03E-01	10
**miR-23b-3p**	1.2	2.64E-01	8.06E-01	154
**miR-148a-3p**	-1.2	2.67E-01	8.08E-01	3832
**miR-200b-3p**	-1.6	2.71E-01	8.15E-01	34
**miR-340-5p**	-1.2	2.82E-01	8.40E-01	173
**miR-625-5p**	-1.4	2.84E-01	8.40E-01	8
**miR-625-3p**	-1.3	2.85E-01	8.40E-01	321
**miR-584-5p**	-1.2	2.94E-01	8.54E-01	723
**miR-494-3p**	1.5	2.95E-01	8.54E-01	12
**miR-23b-5p**	1.5	2.97E-01	8.54E-01	9
**miR-377-3p**	-1.4	3.02E-01	8.54E-01	6
**miR-1468-5p**	-1.3	3.07E-01	8.54E-01	9
**miR-15b-5p**	1.2	3.08E-01	8.54E-01	672
**miR-3942-5p**	-1.5	3.09E-01	8.54E-01	4
**miR-421**	1.2	3.09E-01	8.54E-01	47
**miR-191-3p**	-1.3	3.09E-01	8.54E-01	13
**miR-107**	1.2	3.12E-01	8.54E-01	414
**miR-3187-3p**	1.3	3.12E-01	8.54E-01	25

**miR-628-5p**	-1.5	3.15E-01	8.57E-01	13
**miR-33a-5p**	1.3	3.19E-01	8.61E-01	9
**miR-548d-5p**	1.3	3.25E-01	8.68E-01	16
**miR-5583-3p**	-1.7	3.25E-01	8.68E-01	5
**miR-106b-3p**	-1.2	3.32E-01	8.77E-01	575
**miR-548j-5p**	-1.3	3.33E-01	8.77E-01	31
**miR-4433b-3p**	1.7	3.40E-01	8.90E-01	16
**miR-24-3p**	-1.1	3.42E-01	8.90E-01	583
**let-7e-5p**	1.1	3.44E-01	8.91E-01	214
**miR-214-3p**	1.6	3.49E-01	8.93E-01	7
**miR-151a-5p**	-1.2	3.52E-01	8.93E-01	20
**miR-636**	-1.2	3.56E-01	8.93E-01	16
**miR-181a-5p**	-1.2	3.56E-01	8.93E-01	1338
**miR-3605-5p**	1.3	3.63E-01	8.93E-01	8
**miR-3065-5p**	1.4	3.63E-01	8.93E-01	9
**miR-1908-5p**	-1.2	3.66E-01	8.93E-01	49
**miR-197-3p**	-1.2	3.67E-01	8.93E-01	243
**miR-374a-5p**	-1.2	3.67E-01	8.93E-01	86
**miR-671-3p**	-1.3	3.68E-01	8.93E-01	16
**miR-1976**	-1.2	3.69E-01	8.93E-01	42
**miR-3615**	-1.2	3.70E-01	8.93E-01	744
**miR-542-3p**	-1.2	3.73E-01	8.94E-01	20
**miR-337-5p**	-1.4	3.78E-01	9.01E-01	5
**miR-409-5p**	-1.6	3.80E-01	9.01E-01	5
**miR-199a-3p**	-1.2	3.88E-01	9.13E-01	1591
**miR-190a-5p**	1.2	3.94E-01	9.13E-01	95
**miR-34a-5p**	1.3	3.96E-01	9.13E-01	56
**miR-134-5p**	-1.3	3.97E-01	9.13E-01	152
**miR-3158-3p**	-1.3	3.98E-01	9.13E-01	15
**miR-664a-3p**	-1.3	3.99E-01	9.13E-01	9
**miR-146b-5p**	-1.2	4.04E-01	9.13E-01	335
**miR-509-3p**	1.6	4.05E-01	9.13E-01	5
**miR-6514-5p**	-1.4	4.07E-01	9.13E-01	6
**miR-370-3p**	-1.4	4.08E-01	9.13E-01	32
**miR-5010-5p**	-1.2	4.10E-01	9.13E-01	8
**miR-19a-3p**	1.1	4.12E-01	9.13E-01	140
**miR-181d-5p**	-1.3	4.13E-01	9.13E-01	14
**miR-454-3p**	1.2	4.17E-01	9.16E-01	90
**miR-342-3p**	-1.1	4.19E-01	9.16E-01	3341
**miR-140-3p**	1.2	4.21E-01	9.16E-01	963
**miR-6515-3p**	-1.3	4.23E-01	9.16E-01	5

**miR-487b-3p**	-1.4	4.26E-01	9.16E-01	8
**miR-589-5p**	-1.2	4.27E-01	9.16E-01	21
**miR-92a-3p**	-1.1	4.30E-01	9.19E-01	127379
**miR-328-3p**	-1.2	4.34E-01	9.21E-01	648
**miR-199b-3p**	-1.2	4.39E-01	9.26E-01	1256
**miR-100-5p**	-1.2	4.40E-01	9.26E-01	201
**miR-484**	-1.2	4.48E-01	9.37E-01	2247
**miR-143-3p**	1.2	4.50E-01	9.38E-01	1510
**miR-125b-2-3p**	1.3	4.54E-01	9.38E-01	18
**miR-3940-3p**	-1.3	4.58E-01	9.38E-01	7
**miR-6852-5p**	-1.3	4.58E-01	9.38E-01	22
**miR-345-5p**	-1.2	4.66E-01	9.38E-01	28
**miR-25-5p**	1.2	4.66E-01	9.38E-01	40
**miR-125a-3p**	1.5	4.67E-01	9.38E-01	6
**miR-6793-5p**	-1.2	4.67E-01	9.38E-01	5
**miR-497-5p**	1.4	4.68E-01	9.38E-01	8
**miR-1224-5p**	1.2	4.73E-01	9.40E-01	10
**miR-3177-3p**	-1.3	4.74E-01	9.40E-01	8
**miR-4435**	1.5	4.75E-01	9.40E-01	5
**miR-326**	-1.3	4.78E-01	9.40E-01	24
**miR-22-3p**	1.1	4.91E-01	9.49E-01	685
**miR-3613-5p**	1.1	4.92E-01	9.49E-01	381
**miR-744-5p**	-1.2	4.97E-01	9.49E-01	254
**miR-99b-5p**	-1.1	4.98E-01	9.49E-01	297
**miR-223-5p**	-1.1	5.01E-01	9.49E-01	349
**miR-122-3p**	-1.3	5.03E-01	9.49E-01	6
**miR-4732-5p**	1.1	5.05E-01	9.49E-01	260
**miR-1247-5p**	1.3	5.05E-01	9.49E-01	10
**miR-532-5p**	-1.1	5.09E-01	9.49E-01	289
**miR-122-5p**	-1.2	5.24E-01	9.49E-01	50853
**miR-9-3p**	1.4	5.27E-01	9.49E-01	5
**miR-5001-3p**	-1.2	5.30E-01	9.49E-01	9
**miR-181c-5p**	-1.2	5.32E-01	9.49E-01	9
**miR-18b-3p**	-1.2	5.33E-01	9.49E-01	6
**miR-769-3p**	1.2	5.33E-01	9.49E-01	7
**miR-660-5p**	-1.1	5.34E-01	9.49E-01	470
**miR-181b-5p**	-1.1	5.39E-01	9.49E-01	197
**miR-203a**	-1.3	5.41E-01	9.49E-01	272
**miR-148b-5p**	-1.2	5.45E-01	9.49E-01	13
**miR-30e-3p**	-1.1	5.46E-01	9.49E-01	112
**miR-155-5p**	1.1	5.47E-01	9.49E-01	367

**miR-210-3p**	1.2	5.47E-01	9.49E-01	22
**miR-424-5p**	-1.2	5.51E-01	9.49E-01	13
**miR-502-3p**	1.1	5.54E-01	9.49E-01	46
**miR-192-5p**	-1.1	5.61E-01	9.49E-01	972
**miR-324-3p**	-1.1	5.61E-01	9.49E-01	32
**miR-103a-3p**	-1.1	5.64E-01	9.49E-01	3992
**miR-6786-3p**	-1.2	5.64E-01	9.49E-01	7
**miR-651-5p**	1.3	5.65E-01	9.49E-01	15
**let-7f-5p**	1.1	5.65E-01	9.49E-01	15727
**miR-181a-3p**	1.1	5.65E-01	9.49E-01	26
**miR-31-5p**	1.4	5.69E-01	9.49E-01	10
**miR-96-5p**	1.1	5.70E-01	9.49E-01	144
**miR-598-3p**	-1.1	5.72E-01	9.49E-01	37
**miR-320d**	-1.1	5.75E-01	9.49E-01	68
**miR-3120-3p**	-1.2	5.77E-01	9.49E-01	4
**miR-18a-5p**	-1.1	5.86E-01	9.49E-01	26
**miR-378i**	1.2	5.88E-01	9.49E-01	10
**miR-3138**	1.3	5.90E-01	9.49E-01	8
**miR-1260b**	-1.2	5.91E-01	9.49E-01	10
**miR-26b-5p**	-1.1	5.94E-01	9.49E-01	3842
**miR-627-5p**	-1.2	5.95E-01	9.49E-01	8
**miR-181a-2-3p**	1.1	5.96E-01	9.49E-01	40
**miR-200c-3p**	-1.2	5.96E-01	9.49E-01	91
**miR-4742-3p**	-1.1	5.99E-01	9.49E-01	12
**miR-483-5p**	1.2	6.01E-01	9.49E-01	550
**miR-10a-5p**	1.1	6.01E-01	9.49E-01	978
**miR-1246**	-1.1	6.03E-01	9.49E-01	35
**miR-193b-5p**	1.2	6.03E-01	9.49E-01	78
**miR-186-5p**	-1.1	6.08E-01	9.49E-01	277
**miR-4428**	2.3	6.08E-01	9.49E-01	3
**miR-125a-5p**	1.1	6.08E-01	9.49E-01	2160
**miR-6807-5p**	1.3	6.11E-01	9.49E-01	4
**miR-505-5p**	-1.2	6.13E-01	9.49E-01	14
**miR-126-5p**	1.1	6.14E-01	9.49E-01	3411
**miR-199b-5p**	-1.2	6.14E-01	9.49E-01	8
**miR-320a**	1.1	6.15E-01	9.49E-01	2154
**miR-1260a**	-1.2	6.18E-01	9.49E-01	8
**miR-374b-5p**	-1.1	6.20E-01	9.49E-01	23
**miR-140-5p**	-1.1	6.22E-01	9.49E-01	46
**miR-4533**	-2.0	6.23E-01	9.49E-01	4
**miR-7706**	1.2	6.25E-01	9.49E-01	16

**miR-664a-5p**	1.1	6.27E-01	9.49E-01	128
**miR-629-5p**	-1.1	6.29E-01	9.49E-01	219
**miR-3605-3p**	-1.1	6.30E-01	9.49E-01	40
**let-7a-3p**	1.2	6.30E-01	9.49E-01	9
**miR-1306-5p**	-1.1	6.31E-01	9.49E-01	260
**miR-365a-3p**	-1.2	6.39E-01	9.49E-01	14
**miR-99b-3p**	-1.2	6.39E-01	9.49E-01	9
**miR-26a-5p**	-1.1	6.39E-01	9.49E-01	4679
**miR-676-3p**	1.5	6.42E-01	9.49E-01	5
**miR-193a-5p**	1.1	6.46E-01	9.49E-01	305
**miR-1270**	1.1	6.47E-01	9.49E-01	18
**let-7c-5p**	1.1	6.52E-01	9.49E-01	765
**miR-363-3p**	-1.1	6.56E-01	9.49E-01	510
**miR-196b-5p**	-1.1	6.56E-01	9.49E-01	224
**miR-194-5p**	-1.1	6.61E-01	9.49E-01	461
**miR-7-5p**	1.1	6.61E-01	9.49E-01	659
**miR-146b-3p**	-1.1	6.62E-01	9.49E-01	14
**miR-500a-3p**	1.1	6.62E-01	9.49E-01	39
**miR-941**	-1.1	6.62E-01	9.49E-01	139
**miR-27b-3p**	1.1	6.67E-01	9.51E-01	771
**miR-32-5p**	1.1	6.68E-01	9.51E-01	535
**let-7a-5p**	1.1	6.72E-01	9.52E-01	27324
**miR-127-5p**	-1.5	6.73E-01	9.52E-01	5
**miR-125b-5p**	-1.1	6.82E-01	9.55E-01	1302
**miR-1294**	1.1	6.83E-01	9.55E-01	70
**miR-766-3p**	1.2	6.84E-01	9.55E-01	7
**miR-99a-5p**	-1.1	6.89E-01	9.55E-01	334
**miR-6780a-5p**	1.1	6.96E-01	9.55E-01	6
**miR-548at-5p**	-1.1	6.98E-01	9.55E-01	7
**miR-885-3p**	1.2	7.02E-01	9.55E-01	61
**miR-6862-5p**	1.4	7.03E-01	9.55E-01	5
**miR-454-5p**	1.1	7.04E-01	9.55E-01	21
**miR-1343-3p**	-1.1	7.05E-01	9.55E-01	8
**miR-15b-3p**	-1.1	7.06E-01	9.55E-01	38
**miR-30c-5p**	1.1	7.07E-01	9.55E-01	62
**miR-365b-3p**	-1.1	7.08E-01	9.55E-01	12
**miR-101-3p**	1.1	7.08E-01	9.55E-01	4921
**miR-28-3p**	-1.1	7.10E-01	9.55E-01	359
**miR-139-5p**	-1.1	7.17E-01	9.55E-01	13
**miR-130b-5p**	1.1	7.18E-01	9.55E-01	11
**miR-224-5p**	-1.1	7.20E-01	9.55E-01	23

**miR-301a-3p**	-1.1	7.22E-01	9.55E-01	17
**miR-3200-3p**	1.2	7.25E-01	9.55E-01	9
**miR-21-5p**	1.0	7.27E-01	9.55E-01	23355
**miR-486-3p**	-1.1	7.31E-01	9.55E-01	87
**miR-19b-3p**	1.1	7.32E-01	9.55E-01	570
**miR-132-5p**	1.2	7.34E-01	9.55E-01	7
**miR-148b-3p**	-1.0	7.35E-01	9.55E-01	785
**miR-6803-3p**	-1.1	7.35E-01	9.55E-01	15
**miR-145-5p**	-1.1	7.36E-01	9.55E-01	22
**miR-6511a-3p**	1.1	7.39E-01	9.56E-01	8
**miR-4685-3p**	-1.1	7.41E-01	9.56E-01	8
**miR-874-3p**	1.1	7.44E-01	9.57E-01	32
**miR-106a-5p**	1.1	7.57E-01	9.70E-01	10
**miR-211-5p**	1.2	7.61E-01	9.73E-01	7
**miR-30e-5p**	-1.0	7.75E-01	9.80E-01	4562
**miR-141-3p**	1.1	7.80E-01	9.80E-01	53
**miR-501-3p**	1.0	7.83E-01	9.80E-01	204
**miR-4429**	-1.1	7.85E-01	9.80E-01	7
**miR-760**	-1.1	7.87E-01	9.80E-01	30
**miR-320c**	-1.1	7.89E-01	9.80E-01	129
**miR-25-3p**	-1.0	7.89E-01	9.80E-01	14922
**miR-330-3p**	-1.1	7.91E-01	9.80E-01	7
**miR-191-5p**	-1.0	7.91E-01	9.80E-01	3448
**miR-3173-5p**	1.1	7.95E-01	9.80E-01	22
**miR-185-5p**	1.0	7.96E-01	9.80E-01	3116
**miR-6747-3p**	-1.1	7.96E-01	9.80E-01	6
**miR-3934-5p**	-1.2	7.97E-01	9.80E-01	4
**miR-92b-3p**	-1.0	8.05E-01	9.84E-01	493
**miR-2110**	1.1	8.05E-01	9.84E-01	56
**miR-16-2-3p**	1.0	8.09E-01	9.84E-01	34
**miR-1307-5p**	1.1	8.09E-01	9.84E-01	21
**miR-3198**	-1.0	8.15E-01	9.87E-01	5
**miR-17-3p**	-1.1	8.16E-01	9.87E-01	11
**miR-10a-3p**	1.2	8.18E-01	9.87E-01	9
**miR-130a-3p**	1.1	8.21E-01	9.87E-01	74
**miR-6764-5p**	1.2	8.25E-01	9.89E-01	6
**miR-483-3p**	-1.1	8.27E-01	9.89E-01	260
**miR-503-5p**	1.0	8.30E-01	9.90E-01	38
**miR-423-5p**	-1.0	8.32E-01	9.90E-01	7674
**miR-324-5p**	-1.0	8.40E-01	9.93E-01	86
**miR-1249**	-1.0	8.48E-01	9.93E-01	12

**miR-152-3p**	-1.0	8.54E-01	9.93E-01	156
**miR-16-5p**	1.0	8.59E-01	9.93E-01	334345
**miR-142-5p**	1.0	8.61E-01	9.93E-01	2532
**miR-532-3p**	-1.0	8.61E-01	9.93E-01	56
**miR-2116-3p**	1.1	8.61E-01	9.93E-01	7
**miR-183-5p**	-1.0	8.66E-01	9.93E-01	532
**miR-486-5p**	-1.0	8.70E-01	9.93E-01	217490
**miR-1229-3p**	-1.0	8.70E-01	9.93E-01	7
**miR-320b**	1.0	8.71E-01	9.93E-01	151
**miR-23a-3p**	1.0	8.71E-01	9.93E-01	812
**miR-451a**	-1.0	8.71E-01	9.93E-01	18628
**miR-331-3p**	1.0	8.73E-01	9.93E-01	9
**miR-361-5p**	-1.0	8.75E-01	9.93E-01	257
**miR-429**	-1.0	8.79E-01	9.93E-01	7
**miR-342-5p**	-1.1	8.79E-01	9.93E-01	5
**miR-185-3p**	-1.0	8.81E-01	9.93E-01	66
**miR-190b**	-1.0	8.81E-01	9.93E-01	32
**miR-200a-3p**	1.1	8.81E-01	9.93E-01	34
**miR-148a-5p**	-1.0	8.91E-01	9.96E-01	8
**miR-146a-5p**	-1.0	8.91E-01	9.96E-01	4532
**let-7d-3p**	-1.0	8.92E-01	9.96E-01	287
**miR-144-3p**	-1.0	8.97E-01	9.96E-01	209
**miR-942-5p**	-1.0	9.02E-01	9.96E-01	71
**miR-6511b-3p**	1.1	9.03E-01	9.96E-01	9
**miR-221-3p**	-1.0	9.03E-01	9.96E-01	1293
**miR-20a-5p**	-1.0	9.06E-01	9.96E-01	416
**miR-937-3p**	-1.1	9.07E-01	9.96E-01	5
**miR-450b-5p**	1.0	9.11E-01	9.96E-01	11
**miR-374a-3p**	-1.0	9.12E-01	9.96E-01	11
**miR-1180-3p**	1.0	9.12E-01	9.96E-01	164
**miR-182-5p**	-1.0	9.15E-01	9.97E-01	959
**let-7b-5p**	1.0	9.17E-01	9.97E-01	33604
**miR-548ay-5p**	1.0	9.23E-01	1.00E+00	15
**miR-6805-5p**	1.0	9.26E-01	1.00E+00	7
**let-7i-5p**	1.0	9.31E-01	1.00E+00	17929
**miR-7151-3p**	1.1	9.33E-01	1.00E+00	5
**miR-7-1-3p**	-1.0	9.41E-01	1.00E+00	7
**miR-150-5p**	-1.0	9.41E-01	1.00E+00	2592
**miR-20b-5p**	-1.0	9.49E-01	1.00E+00	223
**miR-378a-5p**	1.0	9.53E-01	1.00E+00	14
**let-7g-5p**	-1.0	9.53E-01	1.00E+00	1408

**miR-3682-3p**	1.0	9.57E-01	1.00E+00	6
**miR-365a-5p**	1.1	9.58E-01	1.00E+00	6
**miR-29c-5p**	1.0	9.60E-01	1.00E+00	12
**miR-375**	1.0	9.61E-01	1.00E+00	465
**miR-330-5p**	1.1	9.66E-01	1.00E+00	4
**miR-98-5p**	1.0	9.68E-01	1.00E+00	137
**miR-142-3p**	-1.0	9.72E-01	1.00E+00	1713
**miR-4446-3p**	1.0	9.72E-01	1.00E+00	13
**miR-92b-5p**	-1.0	9.82E-01	1.00E+00	33
**miR-204-5p**	-1.0	9.88E-01	1.00E+00	12
**miR-590-3p**	-1.0	9.88E-01	1.00E+00	14
**miR-93-5p**	1.0	9.89E-01	1.00E+00	7457
**miR-139-3p**	-1.0	9.89E-01	1.00E+00	173
**miR-3913-5p**	-1.1	9.97E-01	1.00E+00	12
**miR-6796-5p**	1.2	1.00E+00	1.00E+00	4
**miR-3150a-5p**	1.2	1.00E+00	1.00E+00	4
**miR-877-3p**	1.1	1.00E+00	1.00E+00	6
**miR-550a-3p**	1.0	1.00E+00	1.00E+00	13
**miR-3928-3p**	-1.0	1.00E+00	1.00E+00	7
**let-7b-3p**	1.0	1.00E+00	1.00E+00	19
**miR-1296-5p**	-1.0	1.00E+00	1.00E+00	6
**miR-6734-5p**	1.0	1.00E+00	1.00E+00	7
**miR-6842-3p**	1.0	1.00E+00	1.00E+00	7
**miR-26a-1-3p**	-1.0	1.00E+00	1.00E+00	6
**miR-1287-5p**	-1.0	1.00E+00	1.00E+00	17

Table S2. Differential expression analysis of 392 miRNAs detected in plasma from women without ACS (cohort 2) and a history of preeclampsia (PE, n=20) versus normotensive (NT, n=20) pregnancy. MiRNA levels are expressed as mean counts per million mapped reads (CPM). MiRNAs are listed in descending order of statistical significance.

miRNA	Fold Change (PE/NT)	p value	FDR-adjusted p value	miR level (CPM)
**miR-122-5p**	-2.6	4.65E-04	1.82E-01	15405
**miR-29a-3p**	-1.4	1.98E-03	2.37E-01	449
**miR-99a-5p**	-1.5	2.17E-03	2.37E-01	126
**miR-203a**	-2.4	2.67E-03	2.37E-01	251
**miR-125b-5p**	-1.5	3.07E-03	2.37E-01	603
**miR-1299**	4.0	3.63E-03	2.37E-01	10
**miR-205-5p**	-1.6	4.95E-03	2.77E-01	56
**miR-4662a-5p**	-2.0	9.21E-03	4.51E-01	4
**miR-193b-5p**	-2.0	1.11E-02	4.84E-01	17
**miR-9-5p**	-2.9	1.40E-02	5.32E-01	8
**miR-382-3p**	-1.7	1.49E-02	5.32E-01	15
**miR-885-3p**	-2.3	1.74E-02	5.70E-01	15
**miR-206**	-1.8	2.06E-02	6.21E-01	34
**miR-877-3p**	1.6	2.32E-02	6.50E-01	8
**miR-204-5p**	-1.7	2.68E-02	7.02E-01	8
**miR-885-5p**	-2.0	2.95E-02	7.23E-01	8
**miR-22-3p**	1.3	3.47E-02	7.63E-01	506
**miR-376a-3p**	-1.6	3.50E-02	7.63E-01	8
**miR-1224-5p**	2.1	3.78E-02	7.79E-01	10
**miR-30a-5p**	-1.2	4.93E-02	9.18E-01	491
**miR-5189-3p**	-1.7	5.07E-02	9.18E-01	5
**miR-598-3p**	-1.3	5.15E-02	9.18E-01	34
**miR-483-5p**	-1.5	6.03E-02	1.00E+00	118
**miR-329-3p**	-1.7	6.26E-02	1.00E+00	9
**miR-543**	-1.5	7.29E-02	1.00E+00	7
**miR-27b-3p**	-1.2	7.61E-02	1.00E+00	439
**miR-25-5p**	1.3	7.65E-02	1.00E+00	37
**miR-33a-5p**	1.4	7.67E-02	1.00E+00	6
**miR-1294**	1.4	8.99E-02	1.00E+00	56
**miR-652-3p**	1.2	9.10E-02	1.00E+00	52
**miR-625-5p**	1.4	9.20E-02	1.00E+00	8
**miR-378a-5p**	-1.5	9.48E-02	1.00E+00	8
**miR-1287-5p**	1.3	9.80E-02	1.00E+00	14
**miR-28-5p**	-1.5	1.10E-01	1.00E+00	6

**miR-483-3p**	-1.5	1.14E-01	1.00E+00	117
**miR-625-3p**	1.3	1.16E-01	1.00E+00	528
**miR-143-3p**	1.2	1.18E-01	1.00E+00	873
**miR-96-5p**	-1.4	1.21E-01	1.00E+00	93
**miR-181c-5p**	-1.4	1.30E-01	1.00E+00	12
**miR-196b-5p**	1.3	1.31E-01	1.00E+00	202
**miR-485-3p**	-1.4	1.35E-01	1.00E+00	180
**miR-629-5p**	1.2	1.35E-01	1.00E+00	198
**miR-503-5p**	1.3	1.36E-01	1.00E+00	30
**miR-423-5p**	1.2	1.37E-01	1.00E+00	6692
**miR-4646-3p**	-1.3	1.45E-01	1.00E+00	7
**miR-556-3p**	-1.6	1.53E-01	1.00E+00	4
**miR-324-3p**	1.2	1.60E-01	1.00E+00	31
**miR-548a-3p**	-1.6	1.60E-01	1.00E+00	4
**miR-487b-5p**	-1.5	1.66E-01	1.00E+00	5
**miR-548l**	1.3	1.71E-01	1.00E+00	8
**miR-377-3p**	-1.5	1.79E-01	1.00E+00	5
**miR-140-3p**	-1.2	1.80E-01	1.00E+00	650
**miR-2110**	1.2	1.85E-01	1.00E+00	55
**let-7d-3p**	1.1	1.91E-01	1.00E+00	317
**miR-140-5p**	1.2	1.94E-01	1.00E+00	49
**miR-484**	1.2	1.95E-01	1.00E+00	2471
**miR-454-3p**	1.3	1.96E-01	1.00E+00	157
**miR-196a-5p**	1.5	1.99E-01	1.00E+00	7
**miR-3613-5p**	1.3	2.04E-01	1.00E+00	415
**miR-150-5p**	-1.3	2.05E-01	1.00E+00	1679
**miR-142-3p**	1.2	2.10E-01	1.00E+00	1340
**miR-99b-5p**	-1.3	2.10E-01	1.00E+00	511
**miR-130b-3p**	1.2	2.13E-01	1.00E+00	24
**miR-664b-5p**	1.2	2.24E-01	1.00E+00	12
**miR-1**	-1.3	2.27E-01	1.00E+00	132
**miR-17-3p**	-1.3	2.29E-01	1.00E+00	7
**miR-125b-2-3p**	-1.5	2.34E-01	1.00E+00	5
**let-7f-5p**	1.2	2.39E-01	1.00E+00	19987
**miR-7151-3p**	-1.5	2.43E-01	1.00E+00	4
**miR-95-3p**	-1.4	2.43E-01	1.00E+00	6
**miR-494-3p**	-1.3	2.47E-01	1.00E+00	12
**miR-942-5p**	1.2	2.50E-01	1.00E+00	77
**miR-98-5p**	1.1	2.52E-01	1.00E+00	201
**miR-1260a**	1.3	2.55E-01	1.00E+00	10
**miR-338-3p**	-1.2	2.69E-01	1.00E+00	18

**miR-4738-3p**	1.5	2.73E-01	1.00E+00	4
**miR-100-5p**	-1.3	2.74E-01	1.00E+00	85
**miR-3168**	-1.4	2.78E-01	1.00E+00	31
**miR-1538**	1.3	2.78E-01	1.00E+00	5
**miR-548d-5p**	1.3	2.79E-01	1.00E+00	11
**miR-185-3p**	1.1	2.80E-01	1.00E+00	85
**miR-34a-5p**	-1.2	2.80E-01	1.00E+00	14
**miR-382-5p**	-1.3	2.83E-01	1.00E+00	489
**miR-26b-3p**	1.2	2.87E-01	1.00E+00	7
**miR-550a-3p**	1.3	2.87E-01	1.00E+00	11
**miR-6805-5p**	1.3	2.89E-01	1.00E+00	7
**miR-6515-3p**	1.3	2.92E-01	1.00E+00	5
**miR-27a-3p**	-1.1	2.93E-01	1.00E+00	125
**miR-210-3p**	1.3	2.96E-01	1.00E+00	11
**miR-345-5p**	-1.2	3.04E-01	1.00E+00	22
**miR-26b-5p**	1.1	3.04E-01	1.00E+00	5431
**miR-1292-5p**	-1.2	3.05E-01	1.00E+00	9
**miR-3605-3p**	1.2	3.08E-01	1.00E+00	37
**miR-664a-5p**	1.1	3.09E-01	1.00E+00	132
**miR-1908-5p**	1.2	3.15E-01	1.00E+00	75
**miR-16-2-3p**	1.2	3.17E-01	1.00E+00	28
**miR-337-5p**	-1.4	3.17E-01	1.00E+00	5
**miR-192-5p**	-1.2	3.18E-01	1.00E+00	550
**miR-10b-5p**	-1.2	3.20E-01	1.00E+00	525
**miR-148a-3p**	-1.1	3.27E-01	1.00E+00	2413
**miR-181a-2-3p**	-1.2	3.29E-01	1.00E+00	46
**miR-31-5p**	1.7	3.31E-01	1.00E+00	4
**miR-1247-5p**	-1.3	3.32E-01	1.00E+00	7
**miR-3173-5p**	1.1	3.32E-01	1.00E+00	23
**miR-431-5p**	-1.2	3.33E-01	1.00E+00	134
**miR-6741-5p**	1.3	3.34E-01	1.00E+00	6
**miR-186-5p**	1.1	3.34E-01	1.00E+00	279
**miR-532-3p**	1.2	3.37E-01	1.00E+00	42
**let-7b-3p**	-1.2	3.37E-01	1.00E+00	12
**miR-1249**	-1.2	3.40E-01	1.00E+00	19
**miR-374a-3p**	1.2	3.45E-01	1.00E+00	13
**miR-191-5p**	-1.1	3.52E-01	1.00E+00	4263
**miR-342-3p**	-1.2	3.57E-01	1.00E+00	3886
**miR-4742-3p**	1.2	3.60E-01	1.00E+00	16
**miR-4669**	1.6	3.62E-01	1.00E+00	5
**let-7i-5p**	1.1	3.66E-01	1.00E+00	24167

**miR-1296-5p**	1.2	3.66E-01	1.00E+00	8
**miR-4467**	-1.6	3.67E-01	1.00E+00	4
**miR-4732-5p**	1.2	3.67E-01	1.00E+00	172
**miR-3198**	1.2	3.68E-01	1.00E+00	5
**miR-107**	1.2	3.69E-01	1.00E+00	265
**miR-223-5p**	1.1	3.72E-01	1.00E+00	415
**miR-505-5p**	1.2	3.73E-01	1.00E+00	12
**miR-15b-3p**	1.2	3.74E-01	1.00E+00	31
**miR-1246**	-1.2	3.76E-01	1.00E+00	17
**miR-92b-3p**	1.1	3.77E-01	1.00E+00	630
**miR-222-3p**	1.1	3.82E-01	1.00E+00	84
**miR-6786-3p**	1.3	3.86E-01	1.00E+00	6
**miR-136-3p**	-1.3	3.86E-01	1.00E+00	9
**miR-148a-5p**	-1.2	3.87E-01	1.00E+00	8
**miR-1270**	-1.2	3.88E-01	1.00E+00	16
**miR-3613-3p**	-1.3	3.91E-01	1.00E+00	5
**miR-627-5p**	1.2	3.91E-01	1.00E+00	5
**miR-1226-3p**	1.2	3.91E-01	1.00E+00	13
**let-7c-5p**	-1.1	3.92E-01	1.00E+00	579
**miR-221-3p**	-1.1	3.94E-01	1.00E+00	1580
**miR-628-3p**	1.1	3.96E-01	1.00E+00	83
**miR-15b-5p**	1.2	3.96E-01	1.00E+00	474
**miR-487b-3p**	-1.3	4.03E-01	1.00E+00	9
**miR-3187-3p**	-1.1	4.11E-01	1.00E+00	18
**miR-4732-3p**	1.2	4.12E-01	1.00E+00	230
**miR-4435**	1.2	4.14E-01	1.00E+00	4
**miR-320a**	1.1	4.16E-01	1.00E+00	1427
**miR-6764-5p**	1.2	4.17E-01	1.00E+00	4
**miR-491-5p**	1.2	4.18E-01	1.00E+00	17
**miR-365b-3p**	-1.3	4.20E-01	1.00E+00	5
**miR-1976**	1.2	4.20E-01	1.00E+00	48
**miR-139-5p**	-1.2	4.23E-01	1.00E+00	11
**miR-654-3p**	-1.2	4.24E-01	1.00E+00	82
**miR-3928-3p**	-1.2	4.26E-01	1.00E+00	6
**miR-92a-3p**	1.1	4.28E-01	1.00E+00	153484
**let-7d-5p**	1.1	4.30E-01	1.00E+00	1729
**miR-7706**	1.2	4.33E-01	1.00E+00	16
**miR-103a-3p**	1.1	4.38E-01	1.00E+00	6256
**miR-485-5p**	-1.2	4.45E-01	1.00E+00	93
**let-7a-5p**	1.1	4.49E-01	1.00E+00	31676
**miR-376c-3p**	-1.3	4.50E-01	1.00E+00	8

**miR-301a-3p**	1.1	4.57E-01	1.00E+00	21
**miR-125a-5p**	-1.1	4.66E-01	1.00E+00	2273
**miR-486-3p**	1.1	4.66E-01	1.00E+00	100
**miR-30e-5p**	1.1	4.70E-01	1.00E+00	4051
**miR-191-3p**	-1.2	4.78E-01	1.00E+00	17
**miR-29c-5p**	1.1	4.80E-01	1.00E+00	12
**miR-1179**	1.2	4.82E-01	1.00E+00	6
**miR-134-5p**	-1.2	4.82E-01	1.00E+00	241
**miR-411-5p**	-1.2	4.91E-01	1.00E+00	23
**miR-136-5p**	1.2	4.93E-01	1.00E+00	4
**miR-425-5p**	1.1	4.99E-01	1.00E+00	5463
**miR-320d**	-1.1	5.07E-01	1.00E+00	28
**miR-5187-5p**	1.2	5.10E-01	1.00E+00	10
**miR-190a-5p**	1.2	5.11E-01	1.00E+00	159
**miR-7110-3p**	1.3	5.13E-01	1.00E+00	3
**miR-193a-5p**	-1.1	5.24E-01	1.00E+00	88
**miR-493-3p**	-1.2	5.24E-01	1.00E+00	14
**miR-532-5p**	1.1	5.24E-01	1.00E+00	213
**miR-106a-5p**	1.1	5.26E-01	1.00E+00	10
**miR-3127-5p**	1.2	5.27E-01	1.00E+00	6
**miR-197-3p**	1.1	5.35E-01	1.00E+00	436
**miR-18b-3p**	-1.2	5.36E-01	1.00E+00	6
**let-7a-3p**	-1.1	5.36E-01	1.00E+00	6
**miR-323b-3p**	-1.2	5.45E-01	1.00E+00	51
**miR-25-3p**	1.1	5.50E-01	1.00E+00	13596
**miR-1343-3p**	-1.1	5.50E-01	1.00E+00	9
**miR-194-5p**	-1.1	5.50E-01	1.00E+00	175
**miR-20b-5p**	-1.1	5.52E-01	1.00E+00	174
**miR-381-3p**	-1.1	5.52E-01	1.00E+00	66
**miR-7976**	1.2	5.57E-01	1.00E+00	10
**miR-1180-3p**	1.1	5.61E-01	1.00E+00	191
**miR-127-5p**	1.1	5.62E-01	1.00E+00	4
**miR-221-5p**	-1.1	5.72E-01	1.00E+00	20
**miR-23b-3p**	-1.0	5.76E-01	1.00E+00	86
**let-7g-5p**	1.1	5.79E-01	1.00E+00	1962
**miR-92b-5p**	1.1	5.80E-01	1.00E+00	47
**miR-3615**	1.1	5.81E-01	1.00E+00	635
**miR-29c-3p**	-1.1	5.81E-01	1.00E+00	648
**miR-32-5p**	1.1	5.82E-01	1.00E+00	308
**miR-5010-3p**	-1.1	5.83E-01	1.00E+00	9
**miR-143-5p**	-1.1	5.94E-01	1.00E+00	8

**miR-324-5p**	1.1	5.96E-01	1.00E+00	128
**miR-375**	-1.1	5.99E-01	1.00E+00	404
**miR-3200-5p**	1.2	6.00E-01	1.00E+00	6
**miR-766-3p**	1.1	6.01E-01	1.00E+00	11
**miR-18a-3p**	-1.1	6.01E-01	1.00E+00	51
**miR-584-5p**	1.1	6.04E-01	1.00E+00	1358
**miR-1260b**	1.1	6.10E-01	1.00E+00	14
**miR-342-5p**	-1.2	6.12E-01	1.00E+00	5
**miR-409-3p**	-1.1	6.12E-01	1.00E+00	846
**miR-10a-5p**	-1.0	6.17E-01	1.00E+00	572
**miR-3200-3p**	1.1	6.17E-01	1.00E+00	7
**miR-7-5p**	1.1	6.17E-01	1.00E+00	545
**miR-378c**	-1.2	6.18E-01	1.00E+00	5
**miR-323a-3p**	-1.1	6.22E-01	1.00E+00	36
**miR-3177-3p**	-1.1	6.23E-01	1.00E+00	8
**miR-3913-5p**	1.1	6.31E-01	1.00E+00	9
**miR-101-3p**	1.1	6.33E-01	1.00E+00	4474
**miR-548ay-5p**	1.1	6.34E-01	1.00E+00	12
**miR-224-5p**	-1.1	6.38E-01	1.00E+00	18
**miR-548j-5p**	1.1	6.38E-01	1.00E+00	54
**miR-146b-5p**	-1.1	6.39E-01	1.00E+00	374
**miR-148b-5p**	-1.1	6.41E-01	1.00E+00	16
**miR-370-3p**	1.1	6.41E-01	1.00E+00	55
**miR-502-3p**	1.1	6.41E-01	1.00E+00	31
**miR-3620-3p**	-1.1	6.48E-01	1.00E+00	4
**miR-320c**	-1.1	6.53E-01	1.00E+00	51
**miR-21-3p**	-1.1	6.57E-01	1.00E+00	9
**miR-4750-5p**	1.2	6.59E-01	1.00E+00	4
**miR-30d-5p**	1.0	6.60E-01	1.00E+00	18694
**miR-152-3p**	1.1	6.64E-01	1.00E+00	142
**miR-941**	1.1	6.66E-01	1.00E+00	172
**miR-21-5p**	1.0	6.67E-01	1.00E+00	15203
**miR-4433-5p**	1.1	6.67E-01	1.00E+00	6
**miR-15a-5p**	1.1	6.68E-01	1.00E+00	409
**miR-3158-3p**	-1.1	6.69E-01	1.00E+00	15
**miR-542-3p**	1.1	6.72E-01	1.00E+00	13
**miR-744-5p**	-1.1	6.75E-01	1.00E+00	455
**miR-589-5p**	-1.1	6.76E-01	1.00E+00	20
**let-7e-5p**	-1.1	6.77E-01	1.00E+00	182
**miR-671-3p**	1.1	6.89E-01	1.00E+00	27
**miR-5698**	1.2	6.90E-01	1.00E+00	5

**miR-423-3p**	1.1	6.92E-01	1.00E+00	1036
**miR-636**	1.1	6.93E-01	1.00E+00	18
**miR-1277-5p**	1.1	6.93E-01	1.00E+00	20
**miR-486-5p**	1.1	6.95E-01	1.00E+00	267381
**miR-505-3p**	-1.1	7.00E-01	1.00E+00	8
**miR-432-5p**	-1.1	7.05E-01	1.00E+00	915
**miR-361-3p**	1.1	7.08E-01	1.00E+00	366
**miR-127-3p**	-1.1	7.10E-01	1.00E+00	24
**miR-3138**	1.1	7.14E-01	1.00E+00	11
**miR-320b**	1.0	7.15E-01	1.00E+00	67
**miR-1273h-3p**	1.1	7.16E-01	1.00E+00	18
**miR-1468-5p**	1.1	7.20E-01	1.00E+00	8
**miR-142-5p**	1.0	7.23E-01	1.00E+00	2015
**miR-6780a-5p**	-1.2	7.23E-01	1.00E+00	5
**miR-18a-5p**	1.1	7.25E-01	1.00E+00	32
**miR-501-5p**	-1.1	7.29E-01	1.00E+00	5
**miR-200a-3p**	1.1	7.30E-01	1.00E+00	17
**miR-130a-3p**	1.0	7.33E-01	1.00E+00	56
**miR-218-5p**	1.3	7.34E-01	1.00E+00	5
**miR-4433b-3p**	1.1	7.36E-01	1.00E+00	44
**miR-452-5p**	-1.1	7.39E-01	1.00E+00	6
**miR-23b-5p**	1.1	7.40E-01	1.00E+00	7
**miR-874-3p**	-1.0	7.41E-01	1.00E+00	15
**miR-760**	-1.1	7.42E-01	1.00E+00	28
**miR-146b-3p**	-1.1	7.43E-01	1.00E+00	12
**miR-664a-3p**	1.1	7.48E-01	1.00E+00	13
**miR-628-5p**	-1.1	7.50E-01	1.00E+00	18
**miR-30e-3p**	-1.0	7.50E-01	1.00E+00	185
**miR-4685-3p**	1.1	7.54E-01	1.00E+00	10
**miR-6881-3p**	-1.1	7.56E-01	1.00E+00	6
**miR-6842-3p**	-1.1	7.57E-01	1.00E+00	10
**miR-590-3p**	-1.0	7.57E-01	1.00E+00	18
**miR-3688-3p**	1.1	7.59E-01	1.00E+00	11
**miR-24-3p**	1.0	7.60E-01	1.00E+00	592
**miR-5193**	1.1	7.63E-01	1.00E+00	5
**miR-145-5p**	1.1	7.63E-01	1.00E+00	15
**miR-6747-3p**	-1.0	7.65E-01	1.00E+00	9
**miR-1237-3p**	-1.1	7.66E-01	1.00E+00	7
**miR-1307-5p**	1.1	7.68E-01	1.00E+00	18
**miR-369-5p**	-1.1	7.69E-01	1.00E+00	34
**miR-181d-5p**	-1.1	7.72E-01	1.00E+00	21

**miR-4446-3p**	-1.1	7.73E-01	1.00E+00	25
**miR-6511a-3p**	1.1	7.74E-01	1.00E+00	9
**miR-5189-5p**	1.1	7.75E-01	1.00E+00	4
**miR-199a-3p**	1.0	7.75E-01	1.00E+00	2127
**miR-769-5p**	-1.0	7.77E-01	1.00E+00	28
**miR-1255b-5p**	1.1	7.78E-01	1.00E+00	44
**miR-4433b-5p**	1.1	7.79E-01	1.00E+00	3099
**miR-16-5p**	1.1	7.79E-01	1.00E+00	310612
**miR-30c-5p**	1.0	7.80E-01	1.00E+00	51
**miR-26a-5p**	1.0	7.81E-01	1.00E+00	5795
**miR-182-5p**	1.1	7.82E-01	1.00E+00	1635
**miR-215-5p**	-1.1	7.82E-01	1.00E+00	31
**miR-495-3p**	-1.1	7.88E-01	1.00E+00	4
**miR-199b-5p**	-1.1	7.93E-01	1.00E+00	6
**miR-190b**	1.0	7.95E-01	1.00E+00	22
**miR-200b-3p**	-1.1	7.97E-01	1.00E+00	15
**miR-379-5p**	-1.1	8.01E-01	1.00E+00	104
**miR-19b-3p**	-1.0	8.04E-01	1.00E+00	388
**miR-126-5p**	1.0	8.09E-01	1.00E+00	3886
**miR-1304-3p**	1.1	8.09E-01	1.00E+00	21
**miR-421**	1.0	8.11E-01	1.00E+00	35
**miR-6803-3p**	-1.0	8.13E-01	1.00E+00	24
**miR-28-3p**	-1.0	8.13E-01	1.00E+00	493
**miR-151a-3p**	-1.0	8.17E-01	1.00E+00	2949
**miR-133a-3p**	1.1	8.19E-01	1.00E+00	26
**miR-146a-3p**	-1.2	8.20E-01	1.00E+00	3
**miR-361-5p**	-1.0	8.22E-01	1.00E+00	225
**miR-1306-5p**	-1.0	8.24E-01	1.00E+00	437
**miR-144-5p**	-1.1	8.33E-01	1.00E+00	147
**miR-126-3p**	-1.0	8.37E-01	1.00E+00	13164
**miR-330-3p**	-1.0	8.38E-01	1.00E+00	9
**miR-199b-3p**	1.0	8.39E-01	1.00E+00	1710
**miR-331-3p**	1.0	8.41E-01	1.00E+00	13
**miR-339-5p**	-1.0	8.44E-01	1.00E+00	231
**miR-424-5p**	1.1	8.44E-01	1.00E+00	7
**miR-378a-3p**	1.0	8.45E-01	1.00E+00	185
**miR-23a-3p**	-1.0	8.45E-01	1.00E+00	785
**miR-26a-1-3p**	-1.1	8.47E-01	1.00E+00	7
**miR-30b-5p**	-1.0	8.47E-01	1.00E+00	19
**miR-183-5p**	-1.0	8.48E-01	1.00E+00	703
**miR-150-3p**	-1.1	8.49E-01	1.00E+00	12

**miR-6741-3p**	-1.0	8.53E-01	1.00E+00	15
**miR-335-5p**	-1.0	8.53E-01	1.00E+00	286
**miR-671-5p**	-1.0	8.53E-01	1.00E+00	65
**let-7b-5p**	1.0	8.56E-01	1.00E+00	30143
**miR-30d-3p**	1.1	8.59E-01	1.00E+00	6
**miR-106b-3p**	-1.0	8.59E-01	1.00E+00	616
**miR-6511b-3p**	-1.0	8.63E-01	1.00E+00	9
**miR-6772-3p**	-1.1	8.64E-01	1.00E+00	8
**miR-1229-3p**	-1.0	8.65E-01	1.00E+00	11
**miR-106b-5p**	-1.0	8.65E-01	1.00E+00	27
**miR-181b-5p**	-1.0	8.66E-01	1.00E+00	183
**miR-20a-5p**	1.0	8.67E-01	1.00E+00	410
**miR-363-3p**	1.0	8.67E-01	1.00E+00	468
**miR-181c-3p**	1.1	8.68E-01	1.00E+00	9
**miR-660-5p**	1.0	8.70E-01	1.00E+00	356
**miR-6721-5p**	1.0	8.74E-01	1.00E+00	13
**miR-181a-5p**	1.0	8.75E-01	1.00E+00	1516
**miR-148b-3p**	1.0	8.76E-01	1.00E+00	902
**miR-151a-5p**	-1.0	8.82E-01	1.00E+00	26
**miR-99b-3p**	-1.0	8.85E-01	1.00E+00	9
**miR-146a-5p**	1.0	8.85E-01	1.00E+00	5516
**miR-296-5p**	-1.0	8.86E-01	1.00E+00	30
**miR-2355-3p**	1.1	8.87E-01	1.00E+00	7
**miR-185-5p**	-1.0	8.96E-01	1.00E+00	2212
**miR-10a-3p**	1.0	8.96E-01	1.00E+00	4
**miR-130b-5p**	1.0	8.98E-01	1.00E+00	15
**miR-30a-3p**	1.0	8.99E-01	1.00E+00	31
**miR-141-3p**	-1.0	9.02E-01	1.00E+00	23
**miR-19a-3p**	-1.0	9.03E-01	1.00E+00	108
**miR-493-5p**	-1.0	9.03E-01	1.00E+00	25
**miR-651-5p**	-1.0	9.06E-01	1.00E+00	13
**miR-29b-3p**	-1.0	9.13E-01	1.00E+00	118
**miR-1307-3p**	1.0	9.13E-01	1.00E+00	1217
**miR-369-3p**	-1.0	9.16E-01	1.00E+00	27
**miR-155-5p**	1.0	9.18E-01	1.00E+00	435
**miR-17-5p**	1.0	9.21E-01	1.00E+00	162
**miR-181a-3p**	-1.0	9.27E-01	1.00E+00	31
**miR-335-3p**	-1.0	9.29E-01	1.00E+00	29
**miR-326**	1.0	9.31E-01	1.00E+00	47
**miR-374b-5p**	1.0	9.31E-01	1.00E+00	31
**miR-501-3p**	1.0	9.33E-01	1.00E+00	144

**miR-4665-5p**	-1.0	9.38E-01	1.00E+00	4
**miR-1301-3p**	-1.0	9.38E-01	1.00E+00	57
**miR-889-3p**	1.0	9.43E-01	1.00E+00	36
**miR-454-5p**	1.0	9.44E-01	1.00E+00	21
**miR-139-3p**	1.0	9.46E-01	1.00E+00	226
**miR-132-3p**	1.0	9.47E-01	1.00E+00	32
**miR-451a**	1.0	9.54E-01	1.00E+00	14636
**miR-22-5p**	-1.0	9.57E-01	1.00E+00	7
**miR-340-5p**	-1.0	9.58E-01	1.00E+00	211
**miR-500a-3p**	-1.0	9.61E-01	1.00E+00	23
**miR-128-3p**	1.0	9.61E-01	1.00E+00	380
**miR-450b-5p**	1.0	9.65E-01	1.00E+00	7
**miR-223-3p**	-1.0	9.70E-01	1.00E+00	9090
**miR-576-5p**	1.0	9.72E-01	1.00E+00	183
**miR-574-3p**	1.0	9.72E-01	1.00E+00	49
**miR-200c-3p**	-1.0	9.77E-01	1.00E+00	58
**miR-339-3p**	1.0	9.78E-01	1.00E+00	44
**miR-6852-5p**	1.0	9.82E-01	1.00E+00	38
**miR-93-3p**	1.0	9.83E-01	1.00E+00	35
**miR-93-5p**	-1.0	9.85E-01	1.00E+00	7863
**miR-144-3p**	-1.0	9.89E-01	1.00E+00	223
**miR-374a-5p**	1.0	9.92E-01	1.00E+00	141
**miR-328-3p**	-1.0	9.95E-01	1.00E+00	1229
**miR-425-3p**	-1.0	9.99E-01	1.00E+00	92
**miR-4533**	-1.1	1.00E+00	1.00E+00	4
**miR-3940-3p**	1.0	1.00E+00	1.00E+00	9
**miR-5010-5p**	1.0	1.00E+00	1.00E+00	7
**miR-195-5p**	-1.0	1.00E+00	1.00E+00	33
**miR-409-5p**	-1.0	1.00E+00	1.00E+00	6
**miR-199a-5p**	-1.0	1.00E+00	1.00E+00	29

Table S3. Differential expression analysis of 458 miRNAs detected in plasma between women with (n=35) and without (n=40) acute coronary syndrome. MiRNA levels are expressed as mean counts per million mapped reads (CPM). MiRNAs are listed in descending order of statistical significance.

miRNA	Fold Change (ACS/non-ACS)	p value	FDR-adjusted p value	miR level (CPM)
miR-208b-3p	148.0	1.18E-23	5.39E-21	37
miR-29a-3p	2.2	3.12E-23	7.14E-21	726
miR-378a-3p	2.4	3.39E-18	5.18E-16	303
miR-30a-5p	2.3	2.48E-17	2.84E-15	828
miR-499a-5p	34.2	8.30E-17	7.60E-15	19
miR-193a-5p	3.3	1.08E-16	8.24E-15	181
miR-34a-5p	3.9	1.32E-16	8.64E-15	32
miR-10b-5p	2.5	4.51E-16	2.53E-14	897
miR-195-5p	2.8	4.98E-16	2.53E-14	61
miR-483-5p	4.4	7.31E-16	3.35E-14	305
miR-320c	2.3	1.01E-13	4.20E-12	84
miR-320d	2.3	1.39E-13	5.30E-12	45
miR-99a-5p	2.4	1.53E-13	5.38E-12	214
miR-320b	2.1	4.78E-12	1.56E-10	103
miR-1306-5p	-1.9	7.19E-12	2.19E-10	369
miR-194-5p	2.5	7.64E-12	2.19E-10	298
miR-184	11.7	9.00E-12	2.43E-10	24
miR-193b-5p	4.3	1.07E-11	2.73E-10	43
miR-29c-3p	1.8	2.87E-11	6.91E-10	896
miR-208a-3p	15.2	7.87E-11	1.80E-09	3
miR-221-5p	-3.4	9.56E-11	2.08E-09	16
miR-206	6.9	4.15E-10	8.65E-09	127
miR-4433b-5p	-4.0	7.04E-10	1.38E-08	2369
miR-6772-3p	-4.1	7.21E-10	1.38E-08	7
miR-378c	2.9	8.02E-10	1.47E-08	9
miR-423-3p	-2.1	1.84E-09	3.24E-08	858
miR-5010-3p	-3.3	2.07E-09	3.52E-08	8
miR-584-5p	-2.2	2.23E-09	3.64E-08	1129
miR-328-3p	-2.3	4.61E-09	7.28E-08	1034
miR-4646-3p	-3.0	5.76E-09	8.79E-08	6
miR-95-3p	2.9	1.03E-08	1.52E-07	11
miR-127-3p	-4.1	1.45E-08	2.07E-07	19
miR-30e-3p	-1.9	1.67E-08	2.32E-07	158

**miR-103a-3p**	-1.7	1.95E-08	2.63E-07	5337
**miR-98-5p**	-1.6	2.16E-08	2.82E-07	174
**let-7i-5p**	-1.4	2.29E-08	2.92E-07	21241
**miR-197-3p**	-2.1	2.60E-08	3.21E-07	375
**miR-1301-3p**	-2.8	2.86E-08	3.45E-07	46
**miR-125b-5p**	2.0	3.51E-08	4.12E-07	899
**miR-339-5p**	-2.4	5.74E-08	6.58E-07	191
**miR-125b-2-3p**	3.6	6.46E-08	7.21E-07	10
**miR-26b-5p**	-1.5	7.87E-08	8.58E-07	4744
**miR-6803-3p**	-2.0	8.60E-08	9.16E-07	21
**miR-744-5p**	-2.1	1.43E-07	1.49E-06	384
**miR-1**	3.4	1.55E-07	1.58E-06	296
**miR-23b-3p**	1.5	1.78E-07	1.76E-06	116
**miR-374a-5p**	-1.8	1.81E-07	1.76E-06	118
**miR-30d-5p**	-1.7	1.93E-07	1.84E-06	16794
**miR-125b-1-3p**	6.3	2.98E-07	2.78E-06	4
**miR-1307-3p**	-1.9	3.30E-07	3.03E-06	1047
**miR-335-3p**	-2.6	4.06E-07	3.65E-06	24
**miR-874-3p**	1.9	5.28E-07	4.65E-06	23
**miR-100-5p**	2.3	5.54E-07	4.79E-06	132
**miR-148a-3p**	1.5	5.72E-07	4.85E-06	3030
**miR-122-5p**	3.1	6.56E-07	5.42E-06	30458
**miR-99b-5p**	-2.0	6.63E-07	5.42E-06	437
**miR-885-3p**	4.1	6.85E-07	5.51E-06	34
**miR-27a-3p**	1.4	7.43E-07	5.87E-06	160
**miR-1229-3p**	-2.6	1.43E-06	1.11E-05	10
**miR-130b-3p**	1.8	1.51E-06	1.16E-05	34
**miR-1273h-3p**	-2.7	1.64E-06	1.23E-05	15
**miR-485-3p**	-3.1	1.72E-06	1.27E-05	136
**miR-365a-3p**	2.8	2.21E-06	1.58E-05	9
**miR-409-3p**	-2.8	2.21E-06	1.58E-05	654
**miR-21-5p**	1.4	2.26E-06	1.59E-05	18833
**miR-3620-3p**	-4.4	2.45E-06	1.70E-05	4
**miR-485-5p**	-2.8	2.56E-06	1.75E-05	73
**miR-543**	-3.1	2.65E-06	1.79E-05	6
**miR-1246**	2.0	2.84E-06	1.88E-05	25
**miR-1908-5p**	-1.9	2.87E-06	1.88E-05	67
**miR-6842-3p**	-2.3	3.32E-06	2.14E-05	9
**miR-192-5p**	1.7	3.75E-06	2.38E-05	730
**miR-652-3p**	-1.7	6.26E-06	3.90E-05	45
**miR-378i**	2.2	6.30E-06	3.90E-05	7

**miR-4446-3p**	-2.8	6.57E-06	4.01E-05	21
**miR-548j-5p**	-2.2	8.20E-06	4.94E-05	46
**miR-6747-3p**	-2.2	8.31E-06	4.94E-05	8
**miR-215-5p**	2.6	8.41E-06	4.94E-05	54
**miR-10a-5p**	1.5	9.16E-06	5.31E-05	752
**miR-326**	-2.6	9.37E-06	5.36E-05	39
**miR-122-3p**	4.6	9.81E-06	5.55E-05	4
**miR-4433-5p**	-2.8	1.20E-05	6.69E-05	6
**miR-5193**	-3.3	1.21E-05	6.70E-05	5
**miR-30a-3p**	1.7	1.28E-05	6.98E-05	43
**miR-493-5p**	-2.8	1.41E-05	7.61E-05	21
**miR-6721-5p**	-2.8	1.69E-05	8.99E-05	11
**miR-889-3p**	-2.9	1.88E-05	9.92E-05	30
**miR-151a-3p**	-1.7	2.18E-05	1.14E-04	2627
**miR-432-5p**	-2.5	2.60E-05	1.34E-04	736
**miR-454-3p**	-1.8	2.69E-05	1.37E-04	126
**miR-27b-3p**	1.5	3.20E-05	1.61E-04	588
**miR-6741-3p**	-2.0	3.24E-05	1.61E-04	14
**miR-126-3p**	-1.5	3.63E-05	1.79E-04	12027
**miR-379-5p**	-2.4	3.82E-05	1.86E-04	85
**miR-1277-5p**	-2.1	3.92E-05	1.87E-04	18
**miR-500a-3p**	1.6	3.95E-05	1.87E-04	30
**miR-671-3p**	-2.3	3.97E-05	1.87E-04	24
**miR-5698**	-4.2	4.21E-05	1.97E-04	5
**miR-331-3p**	-2.1	4.28E-05	1.98E-04	12
**miR-625-3p**	-2.0	4.48E-05	2.05E-04	471
**miR-10b-3p**	2.5	4.63E-05	2.10E-04	6
**miR-1249**	-2.2	5.69E-05	2.56E-04	17
**miR-3065-5p**	2.7	6.32E-05	2.79E-04	6
**miR-26a-5p**	-1.3	6.34E-05	2.79E-04	5364
**miR-1304-3p**	-2.0	6.68E-05	2.91E-04	19
**miR-374b-5p**	-1.6	6.74E-05	2.91E-04	28
**miR-654-3p**	-2.4	7.06E-05	3.02E-04	68
**miR-320a**	1.4	7.83E-05	3.32E-04	1742
**miR-4429**	2.5	8.75E-05	3.65E-04	5
**miR-92b-5p**	-1.6	8.77E-05	3.65E-04	41
**miR-1296-5p**	-2.4	9.09E-05	3.75E-04	8
**miR-182-5p**	-1.7	1.02E-04	4.12E-04	1298
**miR-664a-3p**	-2.1	1.02E-04	4.12E-04	12
**miR-4433b-3p**	-3.7	1.02E-04	4.12E-04	34
**miR-483-3p**	2.1	1.04E-04	4.16E-04	177

**miR-590-3p**	-1.7	1.09E-04	4.32E-04	17
**miR-369-5p**	-2.4	1.20E-04	4.68E-04	28
**miR-141-3p**	2.2	1.27E-04	4.95E-04	37
**miR-323a-3p**	-2.2	1.36E-04	5.24E-04	31
**miR-223-3p**	-1.8	1.37E-04	5.25E-04	8005
**miR-6852-5p**	-2.2	1.46E-04	5.53E-04	33
**miR-1179**	-2.1	1.54E-04	5.78E-04	6
**miR-411-5p**	-2.4	1.59E-04	5.93E-04	20
**miR-3138**	-2.1	1.70E-04	6.30E-04	10
**miR-1260b**	-2.2	1.85E-04	6.78E-04	13
**miR-381-3p**	-2.3	1.90E-04	6.91E-04	54
**miR-628-3p**	-1.6	1.95E-04	7.03E-04	76
**miR-185-3p**	-1.5	2.13E-04	7.63E-04	79
**miR-4685-3p**	-1.8	2.37E-04	8.42E-04	9
**miR-365b-3p**	2.3	2.55E-04	8.97E-04	8
**miR-17-5p**	-1.5	2.76E-04	9.66E-04	142
**miR-382-5p**	-2.1	2.81E-04	9.74E-04	402
**miR-211-5p**	2.5	2.94E-04	1.01E-03	5
**miR-199a-5p**	-2.1	2.98E-04	1.02E-03	25
**miR-181d-5p**	-1.9	3.05E-04	1.03E-03	19
**miR-32-5p**	1.7	3.35E-04	1.13E-03	402
**miR-324-5p**	-1.6	3.40E-04	1.13E-03	108
**let-7g-5p**	-1.4	3.42E-04	1.13E-03	1689
**miR-10a-3p**	2.4	3.63E-04	1.20E-03	6
**miR-5187-5p**	-2.0	3.83E-04	1.25E-03	9
**miR-28-3p**	-1.6	4.20E-04	1.36E-03	454
**miR-151a-5p**	-1.6	4.47E-04	1.43E-03	24
**miR-766-3p**	-2.6	4.47E-04	1.43E-03	10
**miR-130b-5p**	-1.9	4.49E-04	1.43E-03	14
**miR-431-5p**	-2.2	4.74E-04	1.50E-03	112
**miR-133a-3p**	2.5	5.46E-04	1.71E-03	47
**miR-877-3p**	-2.1	5.74E-04	1.79E-03	7
**miR-200b-3p**	2.2	5.79E-04	1.79E-03	23
**miR-181c-5p**	-1.8	6.18E-04	1.89E-03	11
**miR-491-5p**	-2.0	6.21E-04	1.89E-03	15
**miR-181c-3p**	-2.2	6.24E-04	1.89E-03	9
**miR-107**	1.5	6.52E-04	1.97E-03	328
**miR-196a-5p**	2.3	6.81E-04	2.04E-03	11
**miR-4742-3p**	-1.6	6.98E-04	2.07E-03	14
**miR-128-3p**	-1.3	7.01E-04	2.07E-03	364
**miR-1226-3p**	-1.8	7.12E-04	2.09E-03	12

**miR-191-5p**	-1.4	7.54E-04	2.20E-03	3984
**miR-425-3p**	-1.4	8.07E-04	2.34E-03	87
**let-7f-5p**	-1.3	8.27E-04	2.38E-03	17925
**miR-556-3p**	-2.8	8.84E-04	2.53E-03	4
**miR-424-5p**	1.8	9.05E-04	2.57E-03	9
**miR-3591-5p**	4.0	9.51E-04	2.68E-03	3
**miR-365a-5p**	3.8	9.55E-04	2.68E-03	4
**miR-143-5p**	1.9	9.84E-04	2.75E-03	11
**miR-143-3p**	1.6	1.07E-03	2.97E-03	1159
**miR-210-3p**	1.8	1.09E-03	2.99E-03	16
**miR-497-5p**	2.2	1.17E-03	3.22E-03	5
**miR-487b-5p**	-2.4	1.24E-03	3.39E-03	5
**miR-199b-3p**	-1.6	1.34E-03	3.63E-03	1572
**miR-425-5p**	-1.4	1.40E-03	3.77E-03	4999
**miR-31-5p**	3.4	1.41E-03	3.78E-03	6
**miR-26a-1-3p**	-2.1	1.45E-03	3.83E-03	7
**miR-155-5p**	-1.3	1.45E-03	3.83E-03	414
**miR-190a-5p**	-1.7	1.66E-03	4.36E-03	128
**miR-4428**	3.6	1.71E-03	4.48E-03	2
**miR-140-3p**	1.4	1.73E-03	4.48E-03	781
**miR-221-3p**	-1.4	1.73E-03	4.48E-03	1500
**miR-4750-5p**	-2.5	1.75E-03	4.50E-03	4
**miR-676-3p**	2.7	1.79E-03	4.58E-03	3
**miR-370-3p**	-2.1	1.83E-03	4.66E-03	47
**miR-199a-3p**	-1.6	1.90E-03	4.81E-03	1964
**miR-628-5p**	-1.9	1.95E-03	4.90E-03	17
**miR-6807-5p**	3.4	1.97E-03	4.92E-03	3
**miR-150-3p**	1.7	1.98E-03	4.92E-03	16
**miR-7110-3p**	-2.6	2.13E-03	5.27E-03	4
**miR-19b-3p**	1.4	2.19E-03	5.39E-03	467
**miR-493-3p**	-2.1	2.27E-03	5.56E-03	12
**miR-301a-3p**	-1.6	2.29E-03	5.58E-03	20
**miR-323b-3p**	-2.0	2.42E-03	5.86E-03	44
**miR-92b-3p**	-1.4	2.66E-03	6.42E-03	564
**miR-139-3p**	-1.6	2.71E-03	6.50E-03	214
**miR-200a-3p**	1.9	2.77E-03	6.60E-03	25
**miR-330-3p**	-2.2	2.97E-03	7.05E-03	8
**miR-3940-3p**	-1.8	3.14E-03	7.41E-03	8
**miR-329-3p**	-2.1	3.32E-03	7.79E-03	8
**miR-30b-5p**	-1.5	3.43E-03	8.02E-03	17
**miR-1260a**	-1.8	3.46E-03	8.05E-03	10

**miR-4667-5p**	3.5	3.53E-03	8.16E-03	3
**miR-6515-3p**	-1.9	3.60E-03	8.29E-03	5
**miR-452-5p**	2.0	3.66E-03	8.38E-03	8
**miR-28-5p**	-2.0	3.76E-03	8.56E-03	5
**miR-409-5p**	-2.2	3.95E-03	8.95E-03	6
**miR-501-3p**	1.3	4.04E-03	9.09E-03	168
**miR-134-5p**	-1.8	4.06E-03	9.09E-03	206
**miR-4732-5p**	1.4	4.07E-03	9.09E-03	207
**miR-433-3p**	-2.5	4.28E-03	9.51E-03	4
**miR-214-3p**	2.1	4.34E-03	9.59E-03	5
**miR-126-5p**	-1.3	4.35E-03	9.59E-03	3778
**miR-191-3p**	-1.7	4.47E-03	9.80E-03	16
**miR-296-5p**	-1.5	4.57E-03	9.97E-03	27
**miR-4533**	-2.6	4.74E-03	1.03E-02	4
**miR-532-5p**	1.3	5.03E-03	1.09E-02	245
**miR-148b-3p**	-1.3	5.08E-03	1.09E-02	871
**miR-340-5p**	-1.4	5.23E-03	1.12E-02	201
**miR-6881-3p**	-1.8	5.31E-03	1.13E-02	6
**miR-885-5p**	2.1	5.33E-03	1.13E-02	12
**miR-6730-3p**	3.6	5.34E-03	1.13E-02	3
**miR-185-5p**	1.4	5.54E-03	1.16E-02	2589
**miR-18a-3p**	-1.4	5.54E-03	1.16E-02	45
**miR-342-5p**	-1.7	6.13E-03	1.27E-02	5
**miR-15b-5p**	1.4	6.13E-03	1.27E-02	556
**miR-205-5p**	1.5	6.15E-03	1.27E-02	70
**miR-369-3p**	-1.8	6.20E-03	1.27E-02	25
**miR-223-5p**	-1.4	6.22E-03	1.27E-02	400
**miR-502-3p**	1.4	6.93E-03	1.41E-02	37
**miR-146a-5p**	-1.4	7.60E-03	1.54E-02	5306
**miR-148b-5p**	-1.5	7.69E-03	1.55E-02	15
**miR-15a-5p**	1.4	7.80E-03	1.57E-02	489
**let-7d-3p**	-1.3	9.53E-03	1.91E-02	314
**miR-378a-5p**	1.6	9.94E-03	1.98E-02	10
**miR-18a-5p**	-1.4	9.97E-03	1.98E-02	30
**miR-339-3p**	-1.3	1.01E-02	1.99E-02	42
**miR-27a-5p**	2.0	1.04E-02	2.04E-02	4
**miR-5189-5p**	-2.0	1.06E-02	2.07E-02	4
**miR-941**	-1.4	1.16E-02	2.26E-02	159
**miR-542-3p**	1.5	1.25E-02	2.42E-02	16
**miR-181a-2-3p**	-1.4	1.25E-02	2.42E-02	44
**miR-4665-5p**	-1.8	1.40E-02	2.69E-02	5

**miR-150-5p**	1.5	1.40E-02	2.69E-02	2090
**miR-1343-3p**	-1.5	1.45E-02	2.77E-02	9
**miR-96-5p**	1.5	1.49E-02	2.83E-02	113
**miR-92a-3p**	-1.3	1.50E-02	2.83E-02	140962
**miR-660-5p**	1.2	1.64E-02	3.08E-02	407
**miR-374a-3p**	-1.5	1.64E-02	3.08E-02	13
**miR-1538**	-1.8	1.67E-02	3.13E-02	6
**miR-4513**	-5.6	1.85E-02	3.44E-02	4
**miR-6805-5p**	-1.6	1.88E-02	3.47E-02	7
**miR-181a-3p**	-1.5	1.88E-02	3.47E-02	30
**miR-671-5p**	-1.4	1.91E-02	3.52E-02	64
**miR-142-5p**	1.2	2.26E-02	4.15E-02	2247
**miR-22-3p**	1.3	2.28E-02	4.15E-02	584
**miR-342-3p**	-1.3	2.39E-02	4.34E-02	3780
**miR-3200-5p**	-1.6	2.44E-02	4.41E-02	6
**let-7c-5p**	1.2	2.50E-02	4.51E-02	654
**miR-6511a-3p**	-1.5	2.54E-02	4.55E-02	9
**miR-3934-5p**	2.7	2.54E-02	4.55E-02	3
**miR-4714-3p**	1.9	2.61E-02	4.66E-02	4
**miR-200c-3p**	1.4	2.67E-02	4.73E-02	73
**miR-127-5p**	-1.9	2.68E-02	4.74E-02	5
**miR-636**	-1.3	2.90E-02	5.11E-02	17
**miR-190b**	1.3	3.04E-02	5.34E-02	26
**miR-2355-3p**	-1.7	3.11E-02	5.44E-02	7
**miR-376a-3p**	-1.7	3.22E-02	5.62E-02	8
**miR-146b-5p**	-1.3	3.27E-02	5.68E-02	369
**let-7b-3p**	1.4	3.41E-02	5.89E-02	15
**miR-183-5p**	-1.3	3.45E-02	5.94E-02	610
**miR-532-3p**	1.3	4.00E-02	6.85E-02	48
**miR-6741-5p**	-1.5	4.01E-02	6.85E-02	6
**miR-7976**	-1.4	4.12E-02	7.01E-02	10
**miR-1237-3p**	-1.6	4.19E-02	7.10E-02	7
**miR-132-3p**	1.3	4.29E-02	7.26E-02	39
**miR-154-5p**	-1.7	4.41E-02	7.43E-02	4
**miR-222-3p**	1.2	4.58E-02	7.68E-02	98
**miR-29b-3p**	1.2	4.63E-02	7.74E-02	133
**miR-3177-3p**	-1.5	4.86E-02	8.10E-02	8
**miR-486-5p**	-1.3	4.95E-02	8.21E-02	242014
**miR-574-3p**	1.3	5.01E-02	8.28E-02	61
**miR-3605-5p**	1.5	5.02E-02	8.28E-02	6
**miR-29c-5p**	-1.3	5.26E-02	8.63E-02	12

**miR-129-5p**	1.7	5.33E-02	8.71E-02	5
**miR-6767-5p**	1.6	5.34E-02	8.71E-02	5
**miR-6734-5p**	1.7	5.70E-02	9.26E-02	5
**miR-19a-3p**	1.2	5.77E-02	9.33E-02	122
**miR-18b-3p**	-1.5	5.85E-02	9.43E-02	6
**miR-204-5p**	1.4	5.95E-02	9.56E-02	10
**miR-337-5p**	-1.7	6.18E-02	9.90E-02	5
**miR-3064-5p**	-1.5	6.38E-02	1.02E-01	4
**miR-125a-5p**	-1.2	6.40E-02	1.02E-01	2302
**miR-30d-3p**	-1.4	6.55E-02	1.04E-01	6
**miR-3198**	-1.6	6.60E-02	1.04E-01	5
**miR-1299**	2.0	6.63E-02	1.04E-01	16
**miR-2116-3p**	1.5	6.80E-02	1.07E-01	5
**miR-9-3p**	1.8	6.94E-02	1.09E-01	4
**miR-1292-5p**	-1.3	6.99E-02	1.09E-01	9
**miR-6514-5p**	-1.4	7.56E-02	1.17E-01	5
**miR-376c-3p**	-1.7	7.69E-02	1.19E-01	8
**miR-548d-5p**	1.4	7.71E-02	1.19E-01	13
**miR-93-3p**	-1.2	7.85E-02	1.21E-01	34
**miR-421**	1.2	8.11E-02	1.24E-01	40
**miR-145-5p**	1.3	8.51E-02	1.30E-01	18
**miR-148a-5p**	-1.5	8.63E-02	1.31E-01	8
**miR-664a-5p**	-1.2	8.71E-02	1.32E-01	133
**miR-130a-3p**	1.2	8.81E-02	1.33E-01	65
**miR-99b-3p**	-1.5	9.39E-02	1.42E-01	9
**miR-625-5p**	-1.4	9.44E-02	1.42E-01	8
**miR-181a-5p**	-1.2	9.50E-02	1.42E-01	1450
**miR-450b-5p**	1.4	9.65E-02	1.44E-01	9
**miR-24-3p**	-1.2	9.74E-02	1.45E-01	604
**let-7a-5p**	-1.2	9.84E-02	1.46E-01	29100
**miR-20b-5p**	1.2	9.85E-02	1.46E-01	194
**miR-3682-3p**	1.6	9.93E-02	1.46E-01	4
**miR-451a**	1.2	9.98E-02	1.46E-01	16308
**miR-495-3p**	-1.6	1.00E-01	1.46E-01	5
**miR-4435**	-1.7	1.00E-01	1.46E-01	4
**miR-3187-3p**	1.2	1.01E-01	1.47E-01	21
**miR-769-5p**	-1.2	1.04E-01	1.51E-01	28
**miR-144-5p**	1.3	1.05E-01	1.52E-01	166
**miR-382-3p**	-1.4	1.06E-01	1.53E-01	15
**miR-6796-5p**	2.0	1.07E-01	1.54E-01	3
**miR-431-3p**	-1.6	1.08E-01	1.55E-01	4

**miR-4467**	-1.7	1.09E-01	1.55E-01	4
**miR-1976**	-1.2	1.10E-01	1.57E-01	45
**miR-627-5p**	1.5	1.11E-01	1.57E-01	6
**miR-136-5p**	-1.5	1.13E-01	1.59E-01	5
**miR-3173-5p**	-1.2	1.13E-01	1.59E-01	23
**miR-664b-5p**	-1.4	1.14E-01	1.60E-01	12
**miR-942-5p**	-1.2	1.14E-01	1.60E-01	74
**miR-487b-3p**	-1.5	1.17E-01	1.63E-01	9
**miR-125a-3p**	1.5	1.36E-01	1.90E-01	4
**miR-6511b-3p**	-1.3	1.37E-01	1.90E-01	9
**miR-1180-3p**	-1.2	1.39E-01	1.92E-01	175
**miR-145-3p**	1.6	1.40E-01	1.93E-01	4
**miR-338-3p**	-1.3	1.47E-01	2.03E-01	18
**miR-106a-5p**	-1.2	1.49E-01	2.05E-01	10
**miR-361-3p**	-1.2	1.51E-01	2.06E-01	369
**miR-140-5p**	-1.1	1.56E-01	2.12E-01	48
**miR-3679-5p**	1.4	1.58E-01	2.15E-01	6
**miR-486-3p**	-1.2	1.68E-01	2.28E-01	93
**miR-17-3p**	1.3	1.74E-01	2.34E-01	9
**miR-1250-5p**	-1.7	1.77E-01	2.39E-01	3
**miR-377-3p**	-1.4	1.81E-01	2.43E-01	5
**miR-1294**	1.2	1.88E-01	2.51E-01	61
**miR-642a-5p**	1.5	1.93E-01	2.57E-01	4
**miR-146a-3p**	-1.7	1.93E-01	2.57E-01	4
**miR-494-3p**	-1.4	1.95E-01	2.59E-01	12
**miR-5189-3p**	-1.4	2.08E-01	2.75E-01	6
**miR-7-5p**	1.2	2.16E-01	2.85E-01	588
**miR-22-5p**	1.2	2.27E-01	2.99E-01	8
**miR-7706**	-1.2	2.33E-01	3.06E-01	16
**miR-93-5p**	-1.1	2.35E-01	3.08E-01	7639
**miR-7151-3p**	-1.5	2.38E-01	3.11E-01	5
**miR-454-5p**	-1.2	2.42E-01	3.14E-01	21
**miR-23a-3p**	-1.1	2.43E-01	3.15E-01	820
**miR-6786-3p**	-1.2	2.43E-01	3.15E-01	7
**miR-30c-5p**	1.1	2.47E-01	3.19E-01	57
**miR-335-5p**	1.2	2.48E-01	3.20E-01	345
**miR-576-5p**	-1.1	2.49E-01	3.20E-01	177
**miR-6793-5p**	1.4	2.51E-01	3.21E-01	4
**miR-484**	-1.2	2.53E-01	3.23E-01	2367
**let-7a-3p**	1.3	2.62E-01	3.33E-01	7
**miR-106b-3p**	-1.1	2.76E-01	3.50E-01	593

**miR-106b-5p**	-1.2	2.77E-01	3.50E-01	26
**miR-9-5p**	1.4	2.92E-01	3.69E-01	9
**miR-15b-3p**	1.2	2.93E-01	3.69E-01	34
**miR-503-5p**	1.2	3.12E-01	3.92E-01	34
**miR-4732-3p**	-1.1	3.13E-01	3.92E-01	217
**miR-7-1-3p**	1.2	3.22E-01	4.01E-01	5
**miR-1224-5p**	-1.3	3.22E-01	4.01E-01	10
**miR-3928-3p**	-1.2	3.35E-01	4.15E-01	6
**miR-3615**	1.1	3.42E-01	4.23E-01	682
**miR-142-3p**	1.1	3.48E-01	4.30E-01	1526
**miR-16-2-3p**	1.1	3.51E-01	4.32E-01	30
**miR-548l**	-1.2	3.53E-01	4.33E-01	9
**miR-202-3p**	1.4	3.57E-01	4.38E-01	5
**miR-4775**	-1.1	3.59E-01	4.39E-01	4
**miR-3613-5p**	-1.1	3.62E-01	4.41E-01	391
**miR-4662a-5p**	-1.3	3.65E-01	4.43E-01	5
**miR-3158-3p**	-1.2	3.73E-01	4.52E-01	15
**miR-33a-5p**	1.2	3.78E-01	4.57E-01	7
**miR-136-3p**	-1.2	3.90E-01	4.70E-01	10
**miR-139-5p**	-1.2	4.00E-01	4.81E-01	12
**miR-4669**	1.4	4.10E-01	4.91E-01	7
**miR-330-5p**	1.3	4.12E-01	4.93E-01	3
**miR-1247-5p**	1.2	4.14E-01	4.94E-01	9
**miR-3913-5p**	1.1	4.19E-01	4.99E-01	10
**miR-5001-3p**	1.2	4.43E-01	5.26E-01	7
**miR-345-5p**	1.1	4.48E-01	5.31E-01	25
**miR-760**	-1.1	4.60E-01	5.42E-01	30
**miR-505-5p**	-1.1	4.64E-01	5.47E-01	13
**miR-548n**	-1.2	4.66E-01	5.47E-01	5
**miR-4738-3p**	1.2	4.71E-01	5.51E-01	5
**miR-324-3p**	-1.1	4.77E-01	5.57E-01	32
**let-7b-5p**	1.1	5.01E-01	5.84E-01	31199
**miR-6859-5p**	1.2	5.06E-01	5.88E-01	4
**miR-423-5p**	1.1	5.10E-01	5.91E-01	7124
**miR-186-5p**	-1.1	5.11E-01	5.91E-01	280
**miR-874-5p**	1.3	5.14E-01	5.93E-01	3
**miR-589-5p**	-1.1	5.25E-01	6.04E-01	21
**miR-1468-5p**	-1.1	5.28E-01	6.06E-01	9
**miR-101-3p**	1.1	5.37E-01	6.15E-01	4635
**miR-144-3p**	-1.1	5.43E-01	6.18E-01	213
**miR-598-3p**	-1.1	5.43E-01	6.18E-01	36

**miR-3942-5p**	1.3	5.59E-01	6.35E-01	3
**miR-25-3p**	1.1	5.63E-01	6.38E-01	14035
**miR-5583-3p**	-1.1	5.71E-01	6.46E-01	5
**miR-429**	1.2	5.79E-01	6.53E-01	5
**miR-152-3p**	-1.1	5.80E-01	6.53E-01	153
**miR-23a-5p**	-1.3	5.88E-01	6.60E-01	4
**miR-548at-5p**	1.2	5.91E-01	6.62E-01	6
**miR-3127-5p**	-1.1	5.95E-01	6.65E-01	7
**miR-21-3p**	-1.1	5.97E-01	6.66E-01	10
**miR-30e-5p**	1.0	6.00E-01	6.67E-01	4305
**miR-5010-5p**	-1.0	6.44E-01	7.14E-01	8
**miR-548ay-5p**	1.1	6.46E-01	7.14E-01	14
**miR-2110**	-1.0	6.62E-01	7.29E-01	55
**miR-224-5p**	1.1	6.64E-01	7.29E-01	20
**miR-26b-3p**	-1.1	6.64E-01	7.29E-01	8
**miR-375**	1.1	6.65E-01	7.29E-01	419
**miR-363-3p**	1.1	6.67E-01	7.29E-01	480
**miR-196b-5p**	1.1	6.70E-01	7.31E-01	209
**miR-20a-5p**	-1.0	6.72E-01	7.31E-01	414
**miR-16-5p**	1.1	6.88E-01	7.47E-01	317310
**miR-23b-5p**	-1.1	7.14E-01	7.73E-01	8
**miR-3688-3p**	1.1	7.38E-01	7.97E-01	12
**miR-505-3p**	-1.1	7.54E-01	8.12E-01	9
**miR-4710**	-1.1	7.59E-01	8.14E-01	3
**miR-6764-5p**	1.1	7.59E-01	8.14E-01	5
**miR-629-5p**	1.0	7.66E-01	8.20E-01	207
**miR-1255b-5p**	-1.0	7.69E-01	8.21E-01	44
**miR-5196-3p**	-1.2	7.74E-01	8.24E-01	5
**let-7d-5p**	1.0	7.84E-01	8.34E-01	1747
**miR-6780a-5p**	1.1	7.92E-01	8.36E-01	5
**miR-132-5p**	-1.0	7.92E-01	8.36E-01	6
**miR-550a-3p**	-1.0	7.94E-01	8.36E-01	12
**miR-218-5p**	1.1	7.94E-01	8.36E-01	6
**miR-199b-5p**	1.1	8.12E-01	8.53E-01	7
**miR-1270**	1.1	8.16E-01	8.55E-01	17
**let-7e-5p**	1.0	8.27E-01	8.65E-01	200
**miR-769-3p**	-1.0	8.38E-01	8.74E-01	6
**miR-25-5p**	1.0	8.43E-01	8.77E-01	38
**miR-3168**	1.0	8.44E-01	8.77E-01	32
**miR-3200-3p**	1.0	8.48E-01	8.77E-01	8
**miR-3150a-5p**	-1.2	8.49E-01	8.77E-01	3

**miR-937-3p**	1.1	8.65E-01	8.92E-01	4
**miR-6862-5p**	-1.0	8.77E-01	9.01E-01	4
**miR-146b-3p**	-1.0	8.78E-01	9.01E-01	13
**miR-181b-5p**	-1.0	8.80E-01	9.01E-01	190
**miR-509-3p**	-1.1	8.89E-01	9.09E-01	4
**miR-3613-3p**	-1.0	8.95E-01	9.13E-01	5
**miR-1287-5p**	-1.0	8.98E-01	9.14E-01	16
**miR-1307-5p**	-1.0	9.15E-01	9.30E-01	20
**miR-501-5p**	1.0	9.34E-01	9.47E-01	6
**miR-548a-3p**	1.0	9.39E-01	9.50E-01	5
**miR-361-5p**	1.0	9.45E-01	9.53E-01	243
**miR-203a**	1.0	9.57E-01	9.63E-01	249
**miR-3605-3p**	1.0	9.78E-01	9.82E-01	38
**miR-651-5p**	1.0	9.86E-01	9.89E-01	13
**miR-3120-3p**	-1.0	1.00E+00	1.00E+00	4

Table S4. Overlap in differentially altered miRs (p<0.05) between cohorts 1 and 2 in relation to a history of preeclampsia versus normotensive pregnancy. MiRNA levels are expressed as mean counts per million mapped reads (CPM).

4 common miRNAs	prior PE vs. NT pregnancy (ACS cohort 1)				prior PE vs. NT preg. (non-ACS cohort 2)			
	Fold Change	p value	FDR-adjusted p value	miR level (CPM)	Fold Change	p value	FDR-adjusted p value	miR level (CPM)
miR-1299	4.9	3.67E-03	1.96E-01	23	4.0	3.63E-03	2.37E-01	10
miR-4662a-5p	3.5	1.33E-02	4.45E-01	5	-2.0	9.21E-03	4.51E-01	4
miR-376a-3p	-4.7	1.10E-03	1.17E-01	7	-1.6	3.50E-02	7.63E-01	8
miR-206	-10.6	1.64E-06	6.98E-04	242	-1.8	2.06E-02	6.21E-01	34
26 miRs unique to cohort 1	prior PE vs. NT pregnancy (ACS cohort 1)				prior PE vs. NT preg. (non-ACS cohort 2)			
	Fold Change	p value	FDR-adjusted p value	miR level (CPM)	Fold Change	p value	FDR-adjusted p value	miR level (CPM)
miR-184	10.3	2.35E-04	3.35E-02	52	na	>0.05	na	na
miR-6730-3p	7.3	3.46E-03	1.96E-01	4	na	>0.05	na	na
miR-499a-5p	5.8	1.85E-03	1.58E-01	45	na	>0.05	na	na
miR-218-5p	5.4	2.24E-03	1.60E-01	7	na	>0.05	na	na
miR-3591-5p	3.8	4.01E-02	6.34E-01	5	na	>0.05	na	na
miR-4667-5p	3.6	3.60E-02	5.91E-01	4	na	>0.05	na	na
miR-874-5p	3.3	1.64E-02	4.54E-01	4	na	>0.05	na	na
miR-1	3.0	5.46E-03	2.59E-01	531	na	>0.05	na	na
miR-202-3p	2.9	4.68E-02	6.54E-01	6	na	>0.05	na	na
miR-133a-3p	2.7	2.78E-02	5.16E-01	76	na	>0.05	na	na
miR-6767-5p	2.7	1.95E-02	4.91E-01	7	na	>0.05	na	na
miR-6741-3p	1.7	2.61E-02	5.07E-01	11	na	>0.05	na	na
miR-769-5p	-1.5	4.20E-02	6.40E-01	27	na	>0.05	na	na
miR-30b-5p	-1.8	1.23E-02	4.45E-01	15	na	>0.05	na	na
miR-1277-5p	-1.8	2.35E-02	5.01E-01	13	na	>0.05	na	na
miR-221-5p	-1.9	3.03E-02	5.39E-01	9	na	>0.05	na	na
miR-2355-3p	-1.9	3.54E-02	5.91E-01	7	na	>0.05	na	na
miR-505-3p	-1.9	2.22E-02	5.01E-01	9	na	>0.05	na	na
miR-369-5p	-2.2	2.54E-02	5.07E-01	18	na	>0.05	na	na
miR-493-5p	-2.3	2.29E-02	5.01E-01	13	na	>0.05	na	na
miR-431-5p	-2.4	1.47E-02	4.49E-01	73	na	>0.05	na	na
miR-329-3p	-2.5	4.56E-02	6.54E-01	7	na	>0.05	na	na
miR-136-3p	-2.7	1.35E-02	4.45E-01	10	na	>0.05	na	na
miR-28-5p	-2.7	1.70E-02	4.54E-01	5	na	>0.05	na	na
miR-889-3p	-2.9	7.52E-03	3.21E-01	17	na	>0.05	na	na
miR-1292-5p	-3.6	8.28E-05	1.77E-02	8	na	>0.05	na	na
16 miRs unique to cohort 2	prior PE vs. NT pregnancy (ACS cohort 1)				prior PE vs. NT preg. (non-ACS cohort 2)			
	Fold Change	p value	FDR-adjusted p value	miR level (CPM)	Fold Change	p value	FDR-adjusted p value	miR level (CPM)
miR-1224-5p	na	>0.05	na	na	2.1	3.8E-02	7.8E-01	10
miR-877-3p	na	>0.05	na	na	1.6	2.3E-02	6.5E-01	8
miR-22-3p	na	>0.05	na	na	1.3	3.5E-02	7.6E-01	506
miR-30a-5p	na	>0.05	na	na	-1.2	4.9E-02	9.2E-01	491
miR-29a-3p	na	>0.05	na	na	-1.4	2.0E-03	2.4E-01	449
miR-125b-5p	na	>0.05	na	na	-1.5	3.1E-03	2.4E-01	603
miR-99a-5p	na	>0.05	na	na	-1.5	2.2E-03	2.4E-01	126
miR-205-5p	na	>0.05	na	na	-1.6	5.0E-03	2.8E-01	56
miR-204-5p	na	>0.05	na	na	-1.7	2.7E-02	7.0E-01	8
miR-382-3p	na	>0.05	na	na	-1.7	1.5E-02	5.3E-01	15
miR-193b-5p	na	>0.05	na	na	-2.0	1.1E-02	4.8E-01	17
miR-885-5p	na	>0.05	na	na	-2.0	3.0E-02	7.2E-01	8
miR-885-3p	na	>0.05	na	na	-2.3	1.7E-02	5.7E-01	15
miR-203a	na	>0.05	na	na	-2.4	2.7E-03	2.4E-01	251
miR-122-5p	na	>0.05	na	na	-2.6	4.6E-04	1.8E-01	15405
miR-9-5p	na	>0.05	na	na	-2.9	1.4E-02	5.3E-01	8

Table S5. Overlap in differentially altered miRs (p<0.05) identified in cohorts 1, 2 and 3 in relation to a history of PE or NT pregnancy (cohorts 1 and 2) or acute coronary syndrome (cohort 3). MiRNA levels are expressed as mean counts per million mapped reads (CPM).

1 common miR	prior PE vs. NT pregnancy (ACS cohort 1)				prior PE vs. NT preg. (non-ACS cohort 2)				ACS vs non-ACS (cohort 1 vs cohort 2)			
	Fold Change	p value	FDR-adjusted p value	miR level (CPM)	Fold Change	p value	FDR-adjusted p value	miR level (CPM)	Fold Change	p value	FDR-adjusted p value	miR level (CPM)
miR-206	-10.6	1.64E-06	6.98E-04	242	-1.8	2.06E-02	6.21E-01	34	6.9	4.15E-10	8.65E-09	127
3 miR overlap	prior PE vs. NT pregnancy (ACS cohort 1)				prior PE vs. NT preg. (non-ACS cohort 2)				ACS vs non-ACS (cohort 1 vs cohort 2)			
	Fold Change	p value	FDR-adjusted p value	miR level (CPM)	Fold Change	p value	FDR-adjusted p value	miR level (CPM)	Fold Change	p value	FDR-adjusted p value	miR level (CPM)
miR-1299	4.9	3.67E-03	1.96E-01	23.5	4.0	3.63E-03	2.37E-01	10	na	>0.05	na	na
miR-4662a-5p	3.5	1.33E-02	4.45E-01	5.0	-2.0	9.21E-03	4.51E-01	4	na	>0.05	na	na
miR-376a-3p	-4.7	1.10E-03	1.17E-01	7.0	-1.6	3.50E-02	7.63E-01	8	na	>0.05	na	na
11 miR overlap	prior PE vs. NT pregnancy (ACS cohort 1)				prior PE vs. NT preg. (non-ACS cohort 2)				ACS vs non-ACS (cohort 1 vs cohort 2)			
	Fold Change	p value	FDR-adjusted p value	miR level (CPM)	Fold Change	p value	FDR-adjusted p value	miR level (CPM)	Fold Change	p value	FDR-adjusted p value	miR level (CPM)
miR-877-3p	na	>0.05	na	na	1.6	2.32E-02	6.50E-01	8	-2.1	5.74E-04	1.79E-03	7
miR-22-3p	na	>0.05	na	na	1.3	3.47E-02	7.63E-01	506	1.3	2.28E-02	4.15E-02	584
miR-30a-5p	na	>0.05	na	na	-1.2	4.93E-02	9.18E-01	491	2.3	2.48E-17	2.84E-15	828
miR-29a-3p	na	>0.05	na	na	-1.4	1.98E-03	2.37E-01	449	2.2	3.12E-23	7.14E-21	726
miR-125b-5p	na	>0.05	na	na	-1.5	3.07E-03	2.37E-01	603	2.0	3.51E-08	4.12E-07	899
miR-99a-5p	na	>0.05	na	na	-1.5	2.17E-03	2.37E-01	126	2.4	1.53E-13	5.38E-12	214
miR-205-5p	na	>0.05	na	na	-1.6	4.95E-03	2.77E-01	56	1.5	6.15E-03	1.27E-02	70
miR-193b-5p	na	>0.05	na	na	-2.0	1.11E-02	4.84E-01	17	4.3	1.07E-11	2.73E-10	43
miR-885-5p	na	>0.05	na	na	-2.0	2.95E-02	7.23E-01	8	2.1	5.33E-03	1.13E-02	12
miR-885-3p	na	>0.05	na	na	-2.3	1.74E-02	5.70E-01	15	4.1	6.85E-07	5.51E-06	34
miR-122-5p	na	>0.05	na	na	-2.6	4.65E-04	1.82E-01	15405	3.1	6.56E-07	5.42E-06	30457
17 miR overlap	prior PE vs. NT pregnancy (ACS cohort 1)				prior PE vs. NT preg. (non-ACS cohort 2)				ACS vs non-ACS (cohort 1 vs cohort 2)			
	Fold Change	p value	FDR-adjusted p value	miR level (CPM)	Fold Change	p value	FDR-adjusted p value	miR level (CPM)	Fold Change	p value	FDR-adjusted p value	miR level (CPM)
miR-184	10.3	2.35E-04	3.35E-02	52	na	>0.05	na	na	11.7	9.00E-12	2.43E-10	24
miR-6730-3p	7.3	3.46E-03	1.96E-01	4	na	>0.05	na	na	3.6	5.34E-03	1.13E-02	3
miR-499a-5p	5.8	1.85E-03	1.58E-01	45	na	>0.05	na	na	34.2	8.30E-17	7.60E-15	19
miR-3591-5p	3.8	4.01E-02	6.34E-01	5	na	>0.05	na	na	4.0	9.51E-04	2.68E-03	3
miR-4667-5p	3.6	3.60E-02	5.91E-01	4	na	>0.05	na	na	3.5	3.53E-03	8.16E-03	3
miR-1	3.0	5.46E-03	2.59E-01	531	na	>0.05	na	na	3.4	1.55E-07	1.58E-06	296
miR-133a-3p	2.7	2.78E-02	5.16E-01	76	na	>0.05	na	na	2.5	5.46E-04	1.71E-03	47
miR-6741-3p	1.7	2.61E-02	5.07E-01	11	na	>0.05	na	na	-2.0	3.24E-05	1.61E-04	14
miR-30b-5p	-1.8	1.23E-02	4.45E-01	15	na	>0.05	na	na	-1.5	3.43E-03	8.02E-03	17
miR-1277-5p	-1.8	2.35E-02	5.01E-01	13	na	>0.05	na	na	-2.1	3.92E-05	1.87E-04	18
miR-221-5p	-1.9	3.03E-02	5.39E-01	9	na	>0.05	na	na	-3.4	9.56E-11	2.08E-09	16
miR-369-5p	-2.2	2.54E-02	5.07E-01	18	na	>0.05	na	na	-2.4	1.20E-04	4.68E-04	28
miR-493-5p	-2.3	2.29E-02	5.01E-01	13	na	>0.05	na	na	-2.8	1.41E-05	7.61E-05	21
miR-431-5p	-2.4	1.47E-02	4.49E-01	73	na	>0.05	na	na	-2.2	4.74E-04	1.50E-03	112
miR-329-3p	-2.5	4.56E-02	6.54E-01	7	na	>0.05	na	na	-2.1	3.32E-03	7.79E-03	8
miR-28-5p	-2.7	1.70E-02	4.54E-01	5	na	>0.05	na	na	-2.0	3.76E-03	8.56E-03	5
miR-889-3p	-2.9	7.52E-03	3.21E-01	17	na	>0.05	na	na	-2.9	1.88E-05	9.92E-05	30
5 miRs unique to cohort 2	prior PE vs. NT pregnancy (ACS cohort 1)				prior PE vs. NT preg. (non-ACS cohort 2)				ACS vs non-ACS (cohort 1 vs cohort 2)			
	Fold Change	p value	FDR-adjusted p value	miR level (CPM)	Fold Change	p value	FDR-adjusted p value	miR level (CPM)	Fold Change	p value	FDR-adjusted p value	miR level (CPM)
miR-1224-5p	na	>0.05	na	na	2.1	3.78E-02	7.79E-01	10	na	>0.05	na	na
miR-204-5p	na	>0.05	na	na	-1.7	2.68E-02	7.02E-01	8	na	>0.05	na	na
miR-382-3p	na	>0.05	na	na	-1.7	1.49E-02	5.32E-01	15	na	>0.05	na	na
miR-203a	na	>0.05	na	na	-2.4	2.67E-03	2.37E-01	251	na	>0.05	na	na
miR-9-5p	na	>0.05	na	na	-2.9	1.40E-02	5.32E-01	8	na	>0.05	na	na

Table S5. Continued.

9 miRs unique to cohort 1	prior PE vs. NT pregnancy (ACS cohort 1)				prior PE vs. NT preg. (non-ACS cohort 2)				ACS vs non-ACS (cohort 1 vs cohort 2)			
	Fold Change	p value	FDR-adjusted p value	miR level (CPM)	Fold Change	p value	FDR-adjusted p value	miR level (CPM)	Fold Change	p value	FDR-adjusted p value	miR level (CPM)
miR-218-5p	5.4	2.24E-03	1.60E-01	7	na	>0.05	na	na	na	>0.05	na	na
miR-874-5p	3.3	1.64E-02	4.54E-01	4	na	>0.05	na	na	na	>0.05	na	na
miR-202-3p	2.9	4.68E-02	6.54E-01	6	na	>0.05	na	na	na	>0.05	na	na
miR-6767-5p	2.7	1.95E-02	4.91E-01	7	na	>0.05	na	na	na	>0.05	na	na
miR-769-5p	-1.5	4.20E-02	6.40E-01	27	na	>0.05	na	na	na	>0.05	na	na
miR-2355-3p	-1.9	3.54E-02	5.91E-01	7	na	>0.05	na	na	na	>0.05	na	na
miR-505-3p	-1.9	2.22E-02	5.01E-01	9	na	>0.05	na	na	na	>0.05	na	na
miR-136-3p	-2.7	1.35E-02	4.45E-01	10	na	>0.05	na	na	na	>0.05	na	na
miR-1292-5p	-3.6	8.28E-05	1.77E-02	8	na	>0.05	na	na	na	>0.05	na	na

230 miRs unique to cohort 3	prior PE vs. NT pregnancy (ACS cohort 1)				prior PE vs. NT preg. (non-ACS cohort 2)				ACS vs non-ACS (cohort 1 vs cohort 2)			
	Fold Change	p value	FDR-adjusted p value	miR level (CPM)	Fold Change	p value	FDR-adjusted p value	miR level (CPM)	Fold Change	p value	FDR-adjusted p value	miR level (CPM)
miR-208b-3p	na	>0.05	na	na	na	>0.05	na	na	148.0	1.18E-23	5.39E-21	37
miR-208a-3p	na	>0.05	na	na	na	>0.05	na	na	15.2	7.87E-11	1.80E-09	3
miR-125b-1-3p	na	>0.05	na	na	na	>0.05	na	na	6.3	2.98E-07	2.78E-06	4
miR-122-3p	na	>0.05	na	na	na	>0.05	na	na	4.6	9.81E-06	5.55E-05	4
miR-483-5p	na	>0.05	na	na	na	>0.05	na	na	4.4	7.31E-16	3.35E-14	305
miR-34a-5p	na	>0.05	na	na	na	>0.05	na	na	3.9	1.32E-16	8.64E-15	32
miR-365a-5p	na	>0.05	na	na	na	>0.05	na	na	3.8	9.55E-04	2.68E-03	4
miR-125b-2-3p	na	>0.05	na	na	na	>0.05	na	na	3.6	6.46E-08	7.21E-07	10
miR-4428	na	>0.05	na	na	na	>0.05	na	na	3.6	1.71E-03	4.48E-03	2
miR-6807-5p	na	>0.05	na	na	na	>0.05	na	na	3.4	1.97E-03	4.92E-03	3
miR-31-5p	na	>0.05	na	na	na	>0.05	na	na	3.4	1.41E-03	3.78E-03	6
miR-193a-5p	na	>0.05	na	na	na	>0.05	na	na	3.3	1.08E-16	8.24E-15	181
miR-378c	na	>0.05	na	na	na	>0.05	na	na	2.9	8.02E-10	1.47E-08	9
miR-95-3p	na	>0.05	na	na	na	>0.05	na	na	2.9	1.03E-08	1.52E-07	11
miR-195-5p	na	>0.05	na	na	na	>0.05	na	na	2.8	4.98E-16	2.53E-14	61
miR-365a-3p	na	>0.05	na	na	na	>0.05	na	na	2.8	2.21E-06	1.58E-05	9
miR-3065-5p	na	>0.05	na	na	na	>0.05	na	na	2.7	6.32E-05	2.79E-04	6
miR-3934-5p	na	>0.05	na	na	na	>0.05	na	na	2.7	2.54E-02	4.55E-02	3
miR-676-3p	na	>0.05	na	na	na	>0.05	na	na	2.7	1.79E-03	4.58E-03	3
miR-215-5p	na	>0.05	na	na	na	>0.05	na	na	2.6	8.41E-06	4.94E-05	54
miR-194-5p	na	>0.05	na	na	na	>0.05	na	na	2.5	7.64E-12	2.19E-10	298
miR-10b-5p	na	>0.05	na	na	na	>0.05	na	na	2.5	4.51E-16	2.53E-14	897
miR-211-5p	na	>0.05	na	na	na	>0.05	na	na	2.5	2.94E-04	1.01E-03	5
miR-10b-3p	na	>0.05	na	na	na	>0.05	na	na	2.5	4.63E-05	2.10E-04	6
miR-4429	na	>0.05	na	na	na	>0.05	na	na	2.5	8.75E-05	3.65E-04	5
miR-10a-3p	na	>0.05	na	na	na	>0.05	na	na	2.4	3.63E-04	1.20E-03	6
miR-378a-3p	na	>0.05	na	na	na	>0.05	na	na	2.4	3.39E-18	5.18E-16	303
miR-320c	na	>0.05	na	na	na	>0.05	na	na	2.3	1.01E-13	4.20E-12	84
miR-365b-3p	na	>0.05	na	na	na	>0.05	na	na	2.3	2.55E-04	8.97E-04	8
miR-100-5p	na	>0.05	na	na	na	>0.05	na	na	2.3	5.54E-07	4.79E-06	132
miR-320d	na	>0.05	na	na	na	>0.05	na	na	2.3	1.39E-13	5.30E-12	45
miR-196a-5p	na	>0.05	na	na	na	>0.05	na	na	2.3	6.81E-04	2.04E-03	11
miR-497-5p	na	>0.05	na	na	na	>0.05	na	na	2.2	1.17E-03	3.22E-03	5
miR-378i	na	>0.05	na	na	na	>0.05	na	na	2.2	6.30E-06	3.90E-05	7
miR-141-3p	na	>0.05	na	na	na	>0.05	na	na	2.2	1.27E-04	4.95E-04	37
miR-200b-3p	na	>0.05	na	na	na	>0.05	na	na	2.2	5.79E-04	1.79E-03	23
miR-320b	na	>0.05	na	na	na	>0.05	na	na	2.1	4.78E-12	1.56E-10	103
miR-214-3p	na	>0.05	na	na	na	>0.05	na	na	2.1	4.34E-03	9.59E-03	5
miR-483-3p	na	>0.05	na	na	na	>0.05	na	na	2.1	1.04E-04	4.16E-04	177
miR-27a-5p	na	>0.05	na	na	na	>0.05	na	na	2.0	1.04E-02	2.04E-02	4
miR-452-5p	na	>0.05	na	na	na	>0.05	na	na	2.0	3.66E-03	8.38E-03	8
miR-1246	na	>0.05	na	na	na	>0.05	na	na	2.0	2.84E-06	1.88E-05	25
miR-4714-3p	na	>0.05	na	na	na	>0.05	na	na	1.9	2.61E-02	4.66E-02	4
miR-143-5p	na	>0.05	na	na	na	>0.05	na	na	1.9	9.84E-04	2.75E-03	11
miR-874-3p	na	>0.05	na	na	na	>0.05	na	na	1.9	5.28E-07	4.65E-06	23

Table S5. Continued

miR-200a-3p	na	>0.05	na	na	na	>0.05	na	na	1.9	2.77E-03	6.60E-03	25
miR-424-5p	na	>0.05	na	na	na	>0.05	na	na	1.8	9.05E-04	2.57E-03	9
miR-210-3p	na	>0.05	na	na	na	>0.05	na	na	1.8	1.09E-03	2.99E-03	16
miR-29c-3p	na	>0.05	na	na	na	>0.05	na	na	1.8	2.87E-11	6.91E-10	896
miR-130b-3p	na	>0.05	na	na	na	>0.05	na	na	1.8	1.51E-06	1.16E-05	34
miR-150-3p	na	>0.05	na	na	na	>0.05	na	na	1.7	1.98E-03	4.92E-03	16
miR-32-5p	na	>0.05	na	na	na	>0.05	na	na	1.7	3.35E-04	1.13E-03	402
miR-192-5p	na	>0.05	na	na	na	>0.05	na	na	1.7	3.75E-06	2.38E-05	730
miR-30a-3p	na	>0.05	na	na	na	>0.05	na	na	1.7	1.28E-05	6.98E-05	43
miR-378a-5p	na	>0.05	na	na	na	>0.05	na	na	1.6	9.94E-03	1.98E-02	10
miR-500a-3p	na	>0.05	na	na	na	>0.05	na	na	1.6	3.95E-05	1.87E-04	30
miR-143-3p	na	>0.05	na	na	na	>0.05	na	na	1.6	1.07E-03	2.97E-03	1159
miR-23b-3p	na	>0.05	na	na	na	>0.05	na	na	1.5	1.78E-07	1.76E-06	116
miR-27b-3p	na	>0.05	na	na	na	>0.05	na	na	1.5	3.20E-05	1.61E-04	588
miR-10a-5p	na	>0.05	na	na	na	>0.05	na	na	1.5	9.16E-06	5.31E-05	752
miR-150-5p	na	>0.05	na	na	na	>0.05	na	na	1.5	1.40E-02	2.69E-02	2090
miR-107	na	>0.05	na	na	na	>0.05	na	na	1.5	6.52E-04	1.97E-03	328
miR-96-5p	na	>0.05	na	na	na	>0.05	na	na	1.5	1.49E-02	2.83E-02	113
miR-148a-3p	na	>0.05	na	na	na	>0.05	na	na	1.5	5.72E-07	4.85E-06	3030
miR-542-3p	na	>0.05	na	na	na	>0.05	na	na	1.5	1.25E-02	2.42E-02	16
miR-4732-5p	na	>0.05	na	na	na	>0.05	na	na	1.4	4.07E-03	9.09E-03	207
miR-27a-3p	na	>0.05	na	na	na	>0.05	na	na	1.4	7.43E-07	5.87E-06	160
miR-200c-3p	na	>0.05	na	na	na	>0.05	na	na	1.4	2.67E-02	4.73E-02	73
miR-140-3p	na	>0.05	na	na	na	>0.05	na	na	1.4	1.73E-03	4.48E-03	781
miR-21-5p	na	>0.05	na	na	na	>0.05	na	na	1.4	2.26E-06	1.59E-05	18833
miR-15a-5p	na	>0.05	na	na	na	>0.05	na	na	1.4	7.80E-03	1.57E-02	489
miR-320a	na	>0.05	na	na	na	>0.05	na	na	1.4	7.83E-05	3.32E-04	1741
miR-19b-3p	na	>0.05	na	na	na	>0.05	na	na	1.4	2.19E-03	5.39E-03	467
miR-502-3p	na	>0.05	na	na	na	>0.05	na	na	1.4	6.93E-03	1.41E-02	37
miR-15b-5p	na	>0.05	na	na	na	>0.05	na	na	1.4	6.13E-03	1.27E-02	556
miR-185-5p	na	>0.05	na	na	na	>0.05	na	na	1.4	5.54E-03	1.16E-02	2589
miR-501-3p	na	>0.05	na	na	na	>0.05	na	na	1.3	4.04E-03	9.09E-03	168
miR-532-5p	na	>0.05	na	na	na	>0.05	na	na	1.3	5.03E-03	1.09E-02	245
let-7c-5p	na	>0.05	na	na	na	>0.05	na	na	1.2	2.50E-02	4.51E-02	654
miR-660-5p	na	>0.05	na	na	na	>0.05	na	na	1.2	1.64E-02	3.08E-02	407
miR-142-5p	na	>0.05	na	na	na	>0.05	na	na	1.2	2.26E-02	4.15E-02	2247
miR-92a-3p	na	>0.05	na	na	na	>0.05	na	na	-1.3	1.50E-02	2.83E-02	140957
miR-148b-3p	na	>0.05	na	na	na	>0.05	na	na	-1.3	5.08E-03	1.09E-02	871
let-7d-3p	na	>0.05	na	na	na	>0.05	na	na	-1.3	9.53E-03	1.91E-02	314
miR-126-5p	na	>0.05	na	na	na	>0.05	na	na	-1.3	4.35E-03	9.59E-03	3778
miR-128-3p	na	>0.05	na	na	na	>0.05	na	na	-1.3	7.01E-04	2.07E-03	364
miR-342-3p	na	>0.05	na	na	na	>0.05	na	na	-1.3	2.39E-02	4.34E-02	3780
miR-155-5p	na	>0.05	na	na	na	>0.05	na	na	-1.3	1.45E-03	3.83E-03	414
miR-339-3p	na	>0.05	na	na	na	>0.05	na	na	-1.3	1.01E-02	1.99E-02	42
miR-26a-5p	na	>0.05	na	na	na	>0.05	na	na	-1.3	6.34E-05	2.79E-04	5364
let-7f-5p	na	>0.05	na	na	na	>0.05	na	na	-1.3	8.27E-04	2.38E-03	17925
miR-92b-3p	na	>0.05	na	na	na	>0.05	na	na	-1.4	2.66E-03	6.42E-03	564
miR-941	na	>0.05	na	na	na	>0.05	na	na	-1.4	1.16E-02	2.26E-02	159
miR-425-5p	na	>0.05	na	na	na	>0.05	na	na	-1.4	1.40E-03	3.77E-03	4999
miR-181a-2-3p	na	>0.05	na	na	na	>0.05	na	na	-1.4	1.25E-02	2.42E-02	44
miR-671-5p	na	>0.05	na	na	na	>0.05	na	na	-1.4	1.91E-02	3.52E-02	64

Table S5. Continued

**miR-223-5p**	na	>0.05	na	na	na	>0.05	na	na	-1.4	6.22E-03	1.27E-02	400
**miR-191-5p**	na	>0.05	na	na	na	>0.05	na	na	-1.4	7.54E-04	2.20E-03	3983
**miR-18a-5p**	na	>0.05	na	na	na	>0.05	na	na	-1.4	9.97E-03	1.98E-02	30
**miR-425-3p**	na	>0.05	na	na	na	>0.05	na	na	-1.4	8.07E-04	2.34E-03	87
**miR-340-5p**	na	>0.05	na	na	na	>0.05	na	na	-1.4	5.23E-03	1.12E-02	201
**miR-221-3p**	na	>0.05	na	na	na	>0.05	na	na	-1.4	1.73E-03	4.48E-03	1500
**let-7i-5p**	na	>0.05	na	na	na	>0.05	na	na	-1.4	2.29E-08	2.92E-07	21242
**let-7g-5p**	na	>0.05	na	na	na	>0.05	na	na	-1.4	3.42E-04	1.13E-03	1689
**miR-146a-5p**	na	>0.05	na	na	na	>0.05	na	na	-1.4	7.60E-03	1.54E-02	5306
**miR-18a-3p**	na	>0.05	na	na	na	>0.05	na	na	-1.4	5.54E-03	1.16E-02	45
**miR-6511a-3p**	na	>0.05	na	na	na	>0.05	na	na	-1.5	2.54E-02	4.55E-02	9
**miR-374a-3p**	na	>0.05	na	na	na	>0.05	na	na	-1.5	1.64E-02	3.08E-02	13
**miR-185-3p**	na	>0.05	na	na	na	>0.05	na	na	-1.5	2.13E-04	7.63E-04	79
**miR-181a-3p**	na	>0.05	na	na	na	>0.05	na	na	-1.5	1.88E-02	3.47E-02	30
**miR-17-5p**	na	>0.05	na	na	na	>0.05	na	na	-1.5	2.76E-04	9.66E-04	142
**miR-148b-5p**	na	>0.05	na	na	na	>0.05	na	na	-1.5	7.69E-03	1.55E-02	15
**miR-126-3p**	na	>0.05	na	na	na	>0.05	na	na	-1.5	3.63E-05	1.79E-04	12027
**miR-26b-5p**	na	>0.05	na	na	na	>0.05	na	na	-1.5	7.87E-08	8.58E-07	4744
**miR-296-5p**	na	>0.05	na	na	na	>0.05	na	na	-1.5	4.57E-03	9.97E-03	27
**miR-1343-3p**	na	>0.05	na	na	na	>0.05	na	na	-1.5	1.45E-02	2.77E-02	9
**miR-301a-3p**	na	>0.05	na	na	na	>0.05	na	na	-1.6	2.29E-03	5.58E-03	20
**miR-92b-5p**	na	>0.05	na	na	na	>0.05	na	na	-1.6	8.77E-05	3.65E-04	41
**miR-199a-3p**	na	>0.05	na	na	na	>0.05	na	na	-1.6	1.90E-03	4.81E-03	1964
**miR-139-3p**	na	>0.05	na	na	na	>0.05	na	na	-1.6	2.71E-03	6.50E-03	214
**miR-6805-5p**	na	>0.05	na	na	na	>0.05	na	na	-1.6	1.88E-02	3.47E-02	7
**miR-324-5p**	na	>0.05	na	na	na	>0.05	na	na	-1.6	3.40E-04	1.13E-03	108
**miR-199b-3p**	na	>0.05	na	na	na	>0.05	na	na	-1.6	1.34E-03	3.63E-03	1572
**miR-628-3p**	na	>0.05	na	na	na	>0.05	na	na	-1.6	1.95E-04	7.03E-04	76
**miR-98-5p**	na	>0.05	na	na	na	>0.05	na	na	-1.6	2.16E-08	2.82E-07	174
**miR-28-3p**	na	>0.05	na	na	na	>0.05	na	na	-1.6	4.20E-04	1.36E-03	454
**miR-151a-5p**	na	>0.05	na	na	na	>0.05	na	na	-1.6	4.47E-04	1.43E-03	24
**miR-374b-5p**	na	>0.05	na	na	na	>0.05	na	na	-1.6	6.74E-05	2.91E-04	28
**miR-3200-5p**	na	>0.05	na	na	na	>0.05	na	na	-1.6	2.44E-02	4.41E-02	6
**miR-4742-3p**	na	>0.05	na	na	na	>0.05	na	na	-1.6	6.98E-04	2.07E-03	14
**miR-191-3p**	na	>0.05	na	na	na	>0.05	na	na	-1.7	4.47E-03	9.80E-03	16
**miR-652-3p**	na	>0.05	na	na	na	>0.05	na	na	-1.7	6.26E-06	3.90E-05	45
**miR-30d-5p**	na	>0.05	na	na	na	>0.05	na	na	-1.7	1.93E-07	1.84E-06	16794
**miR-151a-3p**	na	>0.05	na	na	na	>0.05	na	na	-1.7	2.18E-05	1.14E-04	2627
**miR-590-3p**	na	>0.05	na	na	na	>0.05	na	na	-1.7	1.09E-04	4.32E-04	17
**miR-190a-5p**	na	>0.05	na	na	na	>0.05	na	na	-1.7	1.66E-03	4.36E-03	128
**miR-103a-3p**	na	>0.05	na	na	na	>0.05	na	na	-1.7	1.95E-08	2.63E-07	5337
**miR-182-5p**	na	>0.05	na	na	na	>0.05	na	na	-1.7	1.02E-04	4.12E-04	1298
**miR-342-5p**	na	>0.05	na	na	na	>0.05	na	na	-1.7	6.13E-03	1.27E-02	5
**miR-223-3p**	na	>0.05	na	na	na	>0.05	na	na	-1.8	1.37E-04	5.25E-04	8005
**miR-1538**	na	>0.05	na	na	na	>0.05	na	na	-1.8	1.67E-02	3.13E-02	6
**miR-1226-3p**	na	>0.05	na	na	na	>0.05	na	na	-1.8	7.12E-04	2.09E-03	12
**miR-3940-3p**	na	>0.05	na	na	na	>0.05	na	na	-1.8	3.14E-03	7.41E-03	8
**miR-374a-5p**	na	>0.05	na	na	na	>0.05	na	na	-1.8	1.81E-07	1.76E-06	118
**miR-181c-5p**	na	>0.05	na	na	na	>0.05	na	na	-1.8	6.18E-04	1.89E-03	11
**miR-6881-3p**	na	>0.05	na	na	na	>0.05	na	na	-1.8	5.31E-03	1.13E-02	6
**miR-369-3p**	na	>0.05	na	na	na	>0.05	na	na	-1.8	6.20E-03	1.27E-02	25

Table S5. Continued

**miR-134-5p**	na	>0.05	na	na	na	>0.05	na	na	-1.8	4.06E-03	9.09E-03	206
**miR-1260a**	na	>0.05	na	na	na	>0.05	na	na	-1.8	3.46E-03	8.05E-03	10
**miR-4665-5p**	na	>0.05	na	na	na	>0.05	na	na	-1.8	1.40E-02	2.69E-02	5
**miR-454-3p**	na	>0.05	na	na	na	>0.05	na	na	-1.8	2.69E-05	1.37E-04	126
**miR-4685-3p**	na	>0.05	na	na	na	>0.05	na	na	-1.8	2.37E-04	8.42E-04	9
**miR-6515-3p**	na	>0.05	na	na	na	>0.05	na	na	-1.9	3.60E-03	8.29E-03	5
**miR-130b-5p**	na	>0.05	na	na	na	>0.05	na	na	-1.9	4.49E-04	1.43E-03	14
**miR-1908-5p**	na	>0.05	na	na	na	>0.05	na	na	-1.9	2.87E-06	1.88E-05	67
**miR-127-5p**	na	>0.05	na	na	na	>0.05	na	na	-1.9	2.68E-02	4.74E-02	5
**miR-30e-3p**	na	>0.05	na	na	na	>0.05	na	na	-1.9	1.67E-08	2.32E-07	158
**miR-1306-5p**	na	>0.05	na	na	na	>0.05	na	na	-1.9	7.19E-12	2.19E-10	369
**miR-181d-5p**	na	>0.05	na	na	na	>0.05	na	na	-1.9	3.05E-04	1.03E-03	19
**miR-628-5p**	na	>0.05	na	na	na	>0.05	na	na	-1.9	1.95E-03	4.90E-03	17
**miR-1307-3p**	na	>0.05	na	na	na	>0.05	na	na	-1.9	3.30E-07	3.03E-06	1047
**miR-491-5p**	na	>0.05	na	na	na	>0.05	na	na	-2.0	6.21E-04	1.89E-03	15
**miR-6803-3p**	na	>0.05	na	na	na	>0.05	na	na	-2.0	8.60E-08	9.16E-07	21
**miR-5189-5p**	na	>0.05	na	na	na	>0.05	na	na	-2.0	1.06E-02	2.07E-02	4
**miR-323b-3p**	na	>0.05	na	na	na	>0.05	na	na	-2.0	2.42E-03	5.86E-03	44
**miR-625-3p**	na	>0.05	na	na	na	>0.05	na	na	-2.0	4.48E-05	2.05E-04	471
**miR-5187-5p**	na	>0.05	na	na	na	>0.05	na	na	-2.0	3.83E-04	1.25E-03	9
**miR-99b-5p**	na	>0.05	na	na	na	>0.05	na	na	-2.0	6.63E-07	5.42E-06	437
**miR-1304-3p**	na	>0.05	na	na	na	>0.05	na	na	-2.0	6.68E-05	2.91E-04	19
**miR-26a-1-3p**	na	>0.05	na	na	na	>0.05	na	na	-2.1	1.45E-03	3.83E-03	7
**miR-370-3p**	na	>0.05	na	na	na	>0.05	na	na	-2.1	1.83E-03	4.66E-03	47
**miR-664a-3p**	na	>0.05	na	na	na	>0.05	na	na	-2.1	1.02E-04	4.12E-04	12
**miR-744-5p**	na	>0.05	na	na	na	>0.05	na	na	-2.1	1.43E-07	1.49E-06	384
**miR-1179**	na	>0.05	na	na	na	>0.05	na	na	-2.1	1.54E-04	5.78E-04	6
**miR-331-3p**	na	>0.05	na	na	na	>0.05	na	na	-2.1	4.28E-05	1.98E-04	12
**miR-382-5p**	na	>0.05	na	na	na	>0.05	na	na	-2.1	2.81E-04	9.74E-04	402
**miR-197-3p**	na	>0.05	na	na	na	>0.05	na	na	-2.1	2.60E-08	3.21E-07	375
**miR-493-3p**	na	>0.05	na	na	na	>0.05	na	na	-2.1	2.27E-03	5.56E-03	12
**miR-199a-5p**	na	>0.05	na	na	na	>0.05	na	na	-2.1	2.98E-04	1.02E-03	25
**miR-423-3p**	na	>0.05	na	na	na	>0.05	na	na	-2.1	1.84E-09	3.24E-08	858
**miR-3138**	na	>0.05	na	na	na	>0.05	na	na	-2.1	1.70E-04	6.30E-04	10
**miR-6852-5p**	na	>0.05	na	na	na	>0.05	na	na	-2.2	1.46E-04	5.53E-04	33
**miR-1249**	na	>0.05	na	na	na	>0.05	na	na	-2.2	5.69E-05	2.56E-04	17
**miR-409-5p**	na	>0.05	na	na	na	>0.05	na	na	-2.2	3.95E-03	8.95E-03	6
**miR-584-5p**	na	>0.05	na	na	na	>0.05	na	na	-2.2	2.23E-09	3.64E-08	1129
**miR-548j-5p**	na	>0.05	na	na	na	>0.05	na	na	-2.2	8.20E-06	4.94E-05	46
**miR-323a-3p**	na	>0.05	na	na	na	>0.05	na	na	-2.2	1.36E-04	5.24E-04	31
**miR-1260b**	na	>0.05	na	na	na	>0.05	na	na	-2.2	1.85E-04	6.78E-04	13
**miR-6747-3p**	na	>0.05	na	na	na	>0.05	na	na	-2.2	8.31E-06	4.94E-05	8
**miR-181c-3p**	na	>0.05	na	na	na	>0.05	na	na	-2.2	6.24E-04	1.89E-03	9
**miR-330-3p**	na	>0.05	na	na	na	>0.05	na	na	-2.2	2.97E-03	7.05E-03	8
**miR-328-3p**	na	>0.05	na	na	na	>0.05	na	na	-2.3	4.61E-09	7.28E-08	1034
**miR-6842-3p**	na	>0.05	na	na	na	>0.05	na	na	-2.3	3.32E-06	2.14E-05	9
**miR-381-3p**	na	>0.05	na	na	na	>0.05	na	na	-2.3	1.90E-04	6.91E-04	54
**miR-671-3p**	na	>0.05	na	na	na	>0.05	na	na	-2.3	3.97E-05	1.87E-04	24
**miR-379-5p**	na	>0.05	na	na	na	>0.05	na	na	-2.4	3.82E-05	1.86E-04	85
**miR-339-5p**	na	>0.05	na	na	na	>0.05	na	na	-2.4	5.74E-08	6.58E-07	191
**miR-487b-5p**	na	>0.05	na	na	na	>0.05	na	na	-2.4	1.24E-03	3.39E-03	5

Table S5. Continued

miR-1296-5p	na	>0.05	na	na	na	>0.05	na	na	-2.4	9.09E-05	3.75E-04	8
miR-411-5p	na	>0.05	na	na	na	>0.05	na	na	-2.4	1.59E-04	5.93E-04	20
miR-654-3p	na	>0.05	na	na	na	>0.05	na	na	-2.4	7.06E-05	3.02E-04	68
miR-432-5p	na	>0.05	na	na	na	>0.05	na	na	-2.5	2.60E-05	1.34E-04	736
miR-433-3p	na	>0.05	na	na	na	>0.05	na	na	-2.5	4.28E-03	9.51E-03	4
miR-4750-5p	na	>0.05	na	na	na	>0.05	na	na	-2.5	1.75E-03	4.50E-03	4
miR-7110-3p	na	>0.05	na	na	na	>0.05	na	na	-2.6	2.13E-03	5.27E-03	4
miR-1229-3p	na	>0.05	na	na	na	>0.05	na	na	-2.6	1.43E-06	1.11E-05	10
miR-326	na	>0.05	na	na	na	>0.05	na	na	-2.6	9.37E-06	5.36E-05	39
miR-4533	na	>0.05	na	na	na	>0.05	na	na	-2.6	4.74E-03	1.03E-02	4
miR-335-3p	na	>0.05	na	na	na	>0.05	na	na	-2.6	4.06E-07	3.65E-06	24
miR-766-3p	na	>0.05	na	na	na	>0.05	na	na	-2.6	4.47E-04	1.43E-03	10
miR-1273h-3p	na	>0.05	na	na	na	>0.05	na	na	-2.7	1.64E-06	1.23E-05	15
miR-4433-5p	na	>0.05	na	na	na	>0.05	na	na	-2.8	1.20E-05	6.69E-05	6
miR-485-5p	na	>0.05	na	na	na	>0.05	na	na	-2.8	2.56E-06	1.75E-05	73
miR-1301-3p	na	>0.05	na	na	na	>0.05	na	na	-2.8	2.86E-08	3.45E-07	46
miR-556-3p	na	>0.05	na	na	na	>0.05	na	na	-2.8	8.84E-04	2.53E-03	4
miR-6721-5p	na	>0.05	na	na	na	>0.05	na	na	-2.8	1.69E-05	8.99E-05	11
miR-409-3p	na	>0.05	na	na	na	>0.05	na	na	-2.8	2.21E-06	1.58E-05	654
miR-4446-3p	na	>0.05	na	na	na	>0.05	na	na	-2.8	6.57E-06	4.01E-05	21
miR-4646-3p	na	>0.05	na	na	na	>0.05	na	na	-3.0	5.76E-09	8.79E-08	6
miR-543	na	>0.05	na	na	na	>0.05	na	na	-3.1	2.65E-06	1.79E-05	6
miR-485-3p	na	>0.05	na	na	na	>0.05	na	na	-3.1	1.72E-06	1.27E-05	136
miR-5193	na	>0.05	na	na	na	>0.05	na	na	-3.3	1.21E-05	6.70E-05	5
miR-5010-3p	na	>0.05	na	na	na	>0.05	na	na	-3.3	2.07E-09	3.52E-08	8
miR-4433b-3p	na	>0.05	na	na	na	>0.05	na	na	-3.7	1.02E-04	4.12E-04	34
miR-4433b-5p	na	>0.05	na	na	na	>0.05	na	na	-4.0	7.04E-10	1.38E-08	2369
miR-6772-3p	na	>0.05	na	na	na	>0.05	na	na	-4.1	7.21E-10	1.38E-08	7
miR-127-3p	na	>0.05	na	na	na	>0.05	na	na	-4.1	1.45E-08	2.07E-07	19
miR-5698	na	>0.05	na	na	na	>0.05	na	na	-4.2	4.21E-05	1.97E-04	5
miR-3620-3p	na	>0.05	na	na	na	>0.05	na	na	-4.4	2.45E-06	1.70E-05	4
miR-4513	na	>0.05	na	na	na	>0.05	na	na	-5.6	1.85E-02	3.44E-02	4

Table S6. 104 circulating miRNAs previously identified via high-throughput screening to be differentially altered in plasma, serum or whole blood of women with preeclampsia versus normotensive pregnancy. Data is adapted from a systematic review by Sheikh et al. [4].

MicroRNA ID		Number of Citations	References
Source Nomenclature	Updated Nomenclature		
519d	miR-519d-3p	3	Li et al., 2013[9], Yang et al., 2011[10], Yang et al., 2015[11]
517c	miR-517c-3p	3	Li et al., 2013, Yang et al., 2011, Yang et al., 2015
29a	miR-29a-3p	3	Li et al., 2013, Yang et al., 2011, Yang et al., 2015
144	miR-144-3p	3	Li et al., 2013, Ura et al., 2014[12], Wu et al., 2012[13]
130a	miR-130a-3p	3	Li et al., 2013, Wu et al., 2012, Yang et al., 2015
18a	miR-18a-5p	2	Li et al., 2013, Yang et al., 2015
125b	miR-125b-5p	2	Li et al., 2013, Yang et al., 2011
27a	miR-27a-3p	2	Li et al., 2013, Yang et al., 2015
24	miR-24-3p	2	Li et al., 2013, Wu et al., 2012
518b	miR-518b	2	Li et al., 2013, Ura et al., 2014
25	miR-25-3p	2	Li et al., 2013, Ura et al., 2014
223	miR-223-3p	2	Li et al., 2013, Yang et al., 2011
185	miR-185-5p	2	Li et al., 2013, Yang et al., 2011
126	miR-126-3p	2	Ura et al., 2014, Yang et al., 2015
518e	miR-518e-3p	2	Yang et al., 2011, Yang et al., 2015
19a	miR-19a-3p	1	Li et al., 2013
101	miR-101-3p	1	Li et al., 2013
26b	miR-26b-5p	1	Li et al., 2013
378	miR-378a-3p	1	Li et al., 2013
144*	miR-144-5p	1	Li et al., 2013
182	miR-182-5p	1	Li et al., 2013
29c	miR-29c-3p	1	Li et al., 2013
518c	miR-518c-3p	1	Li et al., 2013
515-3p	miR-515-3p	1	Li et al., 2013
424	miR-424-5p	1	Li et al., 2013
29b	miR-29b-3p	1	Li et al., 2013
21	miR-21-5p	1	Li et al., 2013
19b	miR-19b-3p	1	Li et al., 2013
451	miR-451a	1	Li et al., 2013
210	miR-210-3p	1	Ura et al., 2014
32	miR-32-5p	1	Ura et al., 2014
204	miR-204-5p	1	Ura et al., 2014
296-5p	miR-296-5p	1	Ura et al., 2014

152	miR-152-3p	1	Ura et al., 2014
335	miR-335-5p	1	Ura et al., 2014
26a	miR-26a-5p	1	Wu et al., 2012
151-3p	miR-151-3p	1	Wu et al., 2012
181a	miR-181a-5p	1	Wu et al., 2012
30d	miR-30d-5p	1	Wu et al., 2012
342-3p	miR-342-3p	1	Wu et al., 2012
16	miR-16-5p	1	Wu et al., 2012
520g	miR-520g-3p	1	Yang et al., 2011
542-3p	miR-542-3p	1	Yang et al., 2011
135b	miR-135b-5p	1	Yang et al., 2015
149	miR-149-5p	1	Yang et al., 2015
188-5p	miR-188-5p	1	Yang et al., 2015
18b	miR-18b-5p	1	Yang et al., 2015
203	miR-203-3p	1	Yang et al., 2015
205	miR-205-5p	1	Yang et al., 2015
224	miR-224-5p	1	Yang et al., 2015
301a	miR-301a-3p	1	Yang et al., 2015
518a-3p	miR-518a-3p	1	Yang et al., 2015
126*	miR-126-5p	1	Yang et al., 2015
142-3p	miR-142-3p	1	Yang et al., 2015
93	miR-93-5p	1	Yang et al., 2015
34a	miR-34a-5p	1	Li et al., 2013
517b	miR-517b-3p	2	Li et al., 2013, Yang et al., 2011
221	miR-221-3p	2	Li et al., 2013, Wu et al., 2012
521	miR-521	2	Li et al., 2013, Yang et al., 2011
519a	miR-519a-3p	2	Li et al., 2013, Yang et al., 2011
520h	miR-520h	2	Li et al., 2013, Yang et al., 2011
125a-5p	miR-125a-5p	2	Li et al., 2013, Yang et al., 2011
145	miR-145-5p	2	Li et al., 2013, Wu et al., 2012
15b	miR-15b-5p	2	Li et al., 2013, Ura et al., 2014
320c	miR-320c	2	Li et al., 2013, Yang et al., 2011
let-7f	let-7f-5p	2	Li et al., 2013, Yang et al., 2011
23a*	miR-23a-5p	1	Akehurst et al., 2015 [14]
196b-5p	miR-196b-5p	1	Akehurst et al., 2015
206-5p	miR-206-5p	1	Akehurst et al., 2015
502-5p	miR-502-5p	1	Akehurst et al., 2015
503-5p	miR-503-5p	1	Akehurst et al., 2015
758-3p	miR-758-3p	1	Akehurst et al., 2015
10a	miR-10a-5p	1	Li et al., 2013
114	miR-114	1	Li et al., 2013

15b*	miR-15b-3p	1	Li et al., 2013
30a	miR-30a-5p	1	Li et al., 2013
519e	miR-519e-3p	1	Li et al., 2013
299a-5p	miR-299a-5p	1	Li et al., 2013
23a	miR-23a-3p	1	Li et al., 2013
23b	miR-23b-3p	1	Li et al., 2013
525-3p	miR-525-3p	1	Li et al., 2013
199a-5p	miR-199a-5p	1	Li et al., 2013
100	miR-100-5p	1	Li et al., 2013
99a	miR-99a-5p	1	Li et al., 2013
512-5p	miR-512-5p	1	Li et al., 2013
30b	miR-30b-5p	1	Li et al., 2013
107	miR-107	1	Li et al., 2013
1233	miR-1233-3p	1	Ura et al., 2014
650	miR-650	1	Ura et al., 2014
520a	miR-520a-3p	1	Ura et al., 2014
215	miR-215-5p	1	Ura et al., 2014
193a-3p	miR-193a-3p	1	Ura et al., 2014
668	miR-668-3p	1	Ura et al., 2014
376a	miR-376a-3p	1	Ura et al., 2014
574-5p	miR-574-5p	1	Wu et al., 2012
130b	miR-130b-3p	1	Wu et al., 2012
103	miR-103a-3p	1	Wu et al., 2012
425	miR-425-5p	1	Wu et al., 2012
136	miR-136-5p	1	Yang et al., 2011
let-7f-1-star	let-7f-1-3p	1	Yang et al., 2011
let-7a-star	let-7a-3p	1	Yang et al., 2011
1260	miR-1260a	1	Yang et al., 2011
let-7d	let-7d-5p	1	Yang et al., 2011
1272	miR-1272	1	Yang et al., 2011

Table S7. Overlap in differentially altered miRs (p<0.05) identified in cohorts 1, 2 and 4 in relation to a history of PE or NT pregnancy (cohort 1 and 2) or current PE versus NT. Only the direction of change in miRNA level is shown for previously published data. Up/Down denotes conflicting information on direction.

	prior PE vs. NT pregnancy (ACS cohort 1)				prior PE vs. NT preg. (non-ACS cohort 2)				current PE vs NT pregnancy (cohort 4; published literature)					
**2 common miRs**	Fold Change	p value	FDR-adjusted p value	miR level (CPM)	Fold Change	p value	FDR-adjusted p value	miR level (CPM)	Li et al 2013	Yang et al 2011	Wu et al. 2012	Yang et al 2015	Ura et al. 2014	Akehurst et al., 2015
miR-206	-10.6	1.64E-06	6.98E-04	242	-1.8	2.06E-02	6.21E-01	25						Up
miR-376a-3p	-4.7	1.10E-03	1.17E-01	7	-1.6	3.50E-02	7.63E-01	23					Down	
	prior PE vs. NT pregnancy (ACS cohort 1)				prior PE vs. NT preg. (non-ACS cohort 2)				current PE vs NT pregnancy (cohort 4; published literature)					
**6 miRs common to cohort 2 and 4**	Fold Change	p value	FDR-adjusted p value	miR level (CPM)	Fold Change	p value	FDR-adjusted p value	miR level (CPM)	Li et al 2013	Yang et al 2011	Wu et al. 2012	Yang et al 2015	Ura et al. 2014	Akehurst et al., 2015
miR-30a-5p	na	>0.05	na	na	-1.2	4.93E-02	9.18E-01	491	Up					
miR-29a-3p	na	>0.05	na	na	-1.4	1.98E-03	2.37E-01	449	Up	Up		Up		
miR-125b-5p	na	>0.05	na	na	-1.5	3.07E-03	2.37E-01	603	Up	Up				
miR-99a-5p	na	>0.05	na	na	-1.5	2.17E-03	2.37E-01	126	Up					
miR-205-5p	na	>0.05	na	na	-1.6	4.95E-03	2.77E-01	56				Up		
miR-204-5p	na	>0.05	na	na	-1.7	2.68E-02	7.02E-01	8					Up	
	prior PE vs. NT pregnancy (ACS cohort 1)				prior PE vs. NT preg. (non-ACS cohort 2)				current PE vs NT pregnancy (cohort 4; published literature)					
**1 miR common to cohort 1 and 4**	Fold Change	p value	FDR-adjusted p value	miR level (CPM)	Fold Change	p value	FDR-adjusted p value	miR level (CPM)	Li et al 2013	Yang et al 2011	Wu et al. 2012	Yang et al 2015	Ura et al. 2014	Akehurst et al., 2015
miR-30b-5p	-1.8	1.23E-02	4.45E-01	14.88265	na	>0.05	na	na	Up					
	prior PE vs. NT pregnancy (ACS cohort 1)				prior PE vs. NT preg. (non-ACS cohort 2)				current PE vs NT pregnancy (cohort 4; published literature)					
**2 common miRs**	Fold Change	p value	FDR-adjusted p value	miR level (CPM)	Fold Change	p value	FDR-adjusted p value	miR level (CPM)	Li et al 2013	Yang et al 2011	Wu et al. 2012	Yang et al 2015	Ura et al. 2014	Akehurst et al., 2015
miR-1299	4.9	3.67E-03	1.96E-01	23	4.0	3.63E-03	2.37E-01	10	na	na	na	na	na	na
miR-4662a-5p	3.5	1.33E-02	4.45E-01	5	-2.0	9.21E-03	4.51E-01	4	na	na	na	na	na	na
	prior PE vs. NT pregnancy (ACS cohort 1)				prior PE vs. NT preg. (non-ACS cohort 2)				current PE vs NT pregnancy (cohort 4; published literature)					
**10 miRs unique to cohort 2**	Fold Change	p value	FDR-adjusted p value	miR level (CPM)	Fold Change	p value	FDR-adjusted p value	miR level (CPM)	Li et al 2013	Yang et al 2011	Wu et al. 2012	Yang et al 2015	Ura et al. 2014	Akehurst et al., 2015
miR-1224-5p	na	>0.05	na	na	2.1	3.78E-02	7.79E-01	10	na	na	na	na	na	na
miR-877-3p	na	>0.05	na	na	1.6	2.32E-02	6.50E-01	8	na	na	na	na	na	na
miR-22-3p	na	>0.05	na	na	1.3	3.47E-02	7.63E-01	506	na	na	na	na	na	na
miR-382-3p	na	>0.05	na	na	-1.7	1.49E-02	5.32E-01	15	na	na	na	na	na	na
miR-193b-5p	na	>0.05	na	na	-2.0	1.11E-02	4.84E-01	17	na	na	na	na	na	na
miR-885-5p	na	>0.05	na	na	-2.0	2.95E-02	7.23E-01	8	na	na	na	na	na	na
miR-885-3p	na	>0.05	na	na	-2.3	1.74E-02	5.70E-01	15	na	na	na	na	na	na
miR-203a	na	>0.05	na	na	-2.4	2.67E-03	2.37E-01	251	na	na	na	na	na	na
miR-122-5p	na	>0.05	na	na	-2.6	4.65E-04	1.82E-01	15405	na	na	na	na	na	na
miR-9-5p	na	>0.05	na	na	-2.9	1.40E-02	5.32E-01	8	na	na	na	na	na	na
	prior PE vs. NT pregnancy (ACS cohort 1)				prior PE vs. NT preg. (non-ACS cohort 2)				current PE vs NT pregnancy (cohort 4; published literature)					
**25 miRs unique to cohort 1**	Fold Change	p value	FDR-adjusted p value	miR level (CPM)	Fold Change	p value	FDR-adjusted p value	miR level (CPM)	Li et al 2013	Yang et al 2011	Wu et al. 2012	Yang et al 2015	Ura et al. 2014	Akehurst et al., 2015
miR-184	10.3	2.35E-04	3.35E-02	52	na	>0.05	na	na	na	na	na	na	na	na
miR-6730-3p	7.3	3.46E-03	1.96E-01	4	na	>0.05	na	na	na	na	na	na	na	na
miR-499a-5p	5.8	1.85E-03	1.58E-01	45	na	>0.05	na	na	na	na	na	na	na	na
miR-218-5p	5.4	2.24E-03	1.60E-01	7	na	>0.05	na	na	na	na	na	na	na	na
miR-3591-5p	3.8	4.01E-02	6.34E-01	5	na	>0.05	na	na	na	na	na	na	na	na
miR-4667-5p	3.6	3.60E-02	5.91E-01	4	na	>0.05	na	na	na	na	na	na	na	na
miR-874-5p	3.3	1.64E-02	4.54E-01	4	na	>0.05	na	na	na	na	na	na	na	na
miR-1	3.0	5.46E-03	2.59E-01	531	na	>0.05	na	na	na	na	na	na	na	na
miR-202-3p	2.9	4.68E-02	6.54E-01	6	na	>0.05	na	na	na	na	na	na	na	na
miR-133a-3p	2.7	2.78E-02	5.16E-01	76	na	>0.05	na	na	na	na	na	na	na	na
miR-6767-5p	2.7	1.95E-02	4.91E-01	7	na	>0.05	na	na	na	na	na	na	na	na
miR-6741-3p	1.7	2.61E-02	5.07E-01	11	na	>0.05	na	na	na	na	na	na	na	na
miR-769-5p	-1.5	4.20E-02	6.40E-01	27	na	>0.05	na	na	na	na	na	na	na	na
miR-1277-5p	-1.8	2.35E-02	5.01E-01	13	na	>0.05	na	na	na	na	na	na	na	na
miR-221-5p	-1.9	3.03E-02	5.39E-01	9	na	>0.05	na	na	na	na	na	na	na	na
miR-2355-3p	-1.9	3.54E-02	5.91E-01	7	na	>0.05	na	na	na	na	na	na	na	na
miR-505-3p	-1.9	2.22E-02	5.01E-01	9	na	>0.05	na	na	na	na	na	na	na	na
miR-369-5p	-2.2	2.54E-02	5.07E-01	18	na	>0.05	na	na	na	na	na	na	na	na
miR-493-5p	-2.3	2.29E-02	5.01E-01	13	na	>0.05	na	na	na	na	na	na	na	na
miR-431-5p	-2.4	1.47E-02	4.49E-01	73	na	>0.05	na	na	na	na	na	na	na	na
miR-329-3p	-2.5	4.56E-02	6.54E-01	7	na	>0.05	na	na	na	na	na	na	na	na
miR-136-3p	-2.7	1.35E-02	4.45E-01	10	na	>0.05	na	na	na	na	na	na	na	na
miR-28-5p	-2.7	1.70E-02	4.54E-01	5	na	>0.05	na	na	na	na	na	na	na	na
miR-889-3p	-2.9	7.52E-03	3.21E-01	17	na	>0.05	na	na	na	na	na	na	na	na
miR-1292-5p	-3.6	8.28E-05	1.77E-02	8	na	>0.05	na	na	na	na	na	na	na	na

Table S7. Continued.

95 miRs unique to cohort 4	prior PE vs. NT pregnancy (ACS cohort 1)				prior PE vs. NT preg. (non-ACS cohort 2)				current PE vs NT pregnancy (cohort 4; published literature)					
	Fold Change	p value	FDR-adjusted p value	miR level (CPM)	Fold Change	p value	FDR-adjusted p value	miR level (CPM)	Li et al 2013	Yang et al 2011	Wu et al. 2012	Yang et al 2015	Ura et al. 2014	Akehurst et al., 2015
let-7a-3p	na	>0.05	na	na	na	>0.05	na	na		Up				
let-7d-5p	na	>0.05	na	na	na	>0.05	na	na		Down				
let-7f-1-3p	na	>0.05	na	na	na	>0.05	na	na		Up				
let-7f-5p	na	>0.05	na	na	na	>0.05	na	na	Down	Down				
miR-100-5p	na	>0.05	na	na	na	>0.05	na	na	Up					
miR-101-3p	na	>0.05	na	na	na	>0.05	na	na	Up					
miR-103a-3p	na	>0.05	na	na	na	>0.05	na	na			Up			
miR-107	na	>0.05	na	na	na	>0.05	na	na	Down					
miR-10a-5p	na	>0.05	na	na	na	>0.05	na	na	Up					
miR-114	na	>0.05	na	na	na	>0.05	na	na	Up					
miR-1233-3p	na	>0.05	na	na	na	>0.05	na	na					Up	
miR-125a-5p	na	>0.05	na	na	na	>0.05	na	na	Up	Up				
miR-126-3p	na	>0.05	na	na	na	>0.05	na	na				Up	Down	
miR-126-5p	na	>0.05	na	na	na	>0.05	na	na				Up/Down		
miR-1260a	na	>0.05	na	na	na	>0.05	na	na		Down				
miR-1272	na	>0.05	na	na	na	>0.05	na	na		Down				
miR-130a-3p	na	>0.05	na	na	na	>0.05	na	na	Up			Up		
miR-130b-3p	na	>0.05	na	na	na	>0.05	na	na			Up			
miR-135b-5p	na	>0.05	na	na	na	>0.05	na	na				Up		
miR-136-5p	na	>0.05	na	na	na	>0.05	na	na		Up				
miR-142-3p	na	>0.05	na	na	na	>0.05	na	na				Up		
miR-144-3p	na	>0.05	na	na	na	>0.05	na	na	Up/down		Down			
miR-144-5p	na	>0.05	na	na	na	>0.05	na	na	Up					
miR-145-5p	na	>0.05	na	na	na	>0.05	na	na	Up		Up			
miR-149-5p	na	>0.05	na	na	na	>0.05	na	na				Up		
miR-151a-3p	na	>0.05	na	na	na	>0.05	na	na			Up			
miR-152-3p	na	>0.05	na	na	na	>0.05	na	na					Up	
miR-15b-3p	na	>0.05	na	na	na	>0.05	na	na	Up					
miR-15b-5p	na	>0.05	na	na	na	>0.05	na	na	Down				Down	
miR-16-5p	na	>0.05	na	na	na	>0.05	na	na			Down			
miR-181a-5p	na	>0.05	na	na	na	>0.05	na	na			Up			
miR-182-5p	na	>0.05	na	na	na	>0.05	na	na	Up					
miR-185-5p	na	>0.05	na	na	na	>0.05	na	na	Down	Down				
miR-188-5p	na	>0.05	na	na	na	>0.05	na	na				Up		
miR-18a-5p	na	>0.05	na	na	na	>0.05	na	na	Up			Up		
miR-18b-5p	na	>0.05	na	na	na	>0.05	na	na				Up		
miR-193a-3p	na	>0.05	na	na	na	>0.05	na	na					Up	
miR-196b-5p	na	>0.05	na	na	na	>0.05	na	na						Up
miR-199a-5p	na	>0.05	na	na	na	>0.05	na	na	Up					
miR-19a-3p	na	>0.05	na	na	na	>0.05	na	na	Up/down					
miR-19b-3p	na	>0.05	na	na	na	>0.05	na	na	Down/Up					
miR-203a-3p	na	>0.05	na	na	na	>0.05	na	na				Up		
miR-21-5p	na	>0.05	na	na	na	>0.05	na	na	Up					
miR-210-3p	na	>0.05	na	na	na	>0.05	na	na					Up	
miR-215-5p	na	>0.05	na	na	na	>0.05	na	na					Up	
miR-221-3p	na	>0.05	na	na	na	>0.05	na	na	Up		Up			
miR-223-3p	na	>0.05	na	na	na	>0.05	na	na	Down	Down				

Table S7. Continued.

**miR-224-5p**	na	>0.05	na	na	na	>0.05	na	na				Up		
**miR-23a-3p**	na	>0.05	na	na	na	>0.05	na	na	Up					
**miR-23a-5p**	na	>0.05	na	na	na	>0.05	na	na						Down
**miR-23b-3p**	na	>0.05	na	na	na	>0.05	na	na	Up					
**miR-24-3p**	na	>0.05	na	na	na	>0.05	na	na	Up					
**miR-25-3p**	na	>0.05	na	na	na	>0.05	na	na	Down/Up					
**miR-26a-5p**	na	>0.05	na	na	na	>0.05	na	na			Up			
**miR-26b-5p**	na	>0.05	na	na	na	>0.05	na	na	UP					
**miR-27a-3p**	na	>0.05	na	na	na	>0.05	na	na	UP			Up		
**miR-296-5p**	na	>0.05	na	na	na	>0.05	na	na					Up	
**miR-29b-3p**	na	>0.05	na	na	na	>0.05	na	na	Up					
**miR-29c-3p**	na	>0.05	na	na	na	>0.05	na	na	Up					
**miR-301a-3p**	na	>0.05	na	na	na	>0.05	na	na				Up		
**miR-30d-5p**	na	>0.05	na	na	na	>0.05	na	na			Up			
**miR-32-5p**	na	>0.05	na	na	na	>0.05	na	na					Up	
**miR-320c**	na	>0.05	na	na	na	>0.05	na	na	Down	Down				
**miR-335-5p**	na	>0.05	na	na	na	>0.05	na	na					Down	
**miR-342-3p**	na	>0.05	na	na	na	>0.05	na	na			Up			
**miR-34a-5p**	na	>0.05	na	na	na	>0.05	na	na	Up					
**miR-378a-3p**	na	>0.05	na	na	na	>0.05	na	na	Up					
**miR-424-5p**	na	>0.05	na	na	na	>0.05	na	na	Up					
**miR-425-5p**	na	>0.05	na	na	na	>0.05	na	na			Up			
**miR-451a**	na	>0.05	na	na	na	>0.05	na	na	Down					
**miR-502-5p**	na	>0.05	na	na	na	>0.05	na	na						Up
**miR-503-5p**	na	>0.05	na	na	na	>0.05	na	na						Up
**miR-512-5p**	na	>0.05	na	na	na	>0.05	na	na	Up					
**miR-515-3p**	na	>0.05	na	na	na	>0.05	na	na	Up					
**miR-517b-3p**	na	>0.05	na	na	na	>0.05	na	na	Up	Up				
**miR-517c-3p**	na	>0.05	na	na	na	>0.05	na	na	Up	Up		Up		
**miR-518a-3p**	na	>0.05	na	na	na	>0.05	na	na				Up		
**miR-518b**	na	>0.05	na	na	na	>0.05	na	na	Up					
**miR-518c-3p**	na	>0.05	na	na	na	>0.05	na	na	Up					
**miR-518e-3p**	na	>0.05	na	na	na	>0.05	na	na		Up		Up		
**miR-519a-3p**	na	>0.05	na	na	na	>0.05	na	na	Up	Up				
**miR-519d-3p**	na	>0.05	na	na	na	>0.05	na	na	Up	Up		Up		
**miR-519e-3p**	na	>0.05	na	na	na	>0.05	na	na	Up					
**miR-520a-3p**	na	>0.05	na	na	na	>0.05	na	na					Up	
**miR-520g-3p**	na	>0.05	na	na	na	>0.05	na	na		Up				
**miR-520h**	na	>0.05	na	na	na	>0.05	na	na	Up	Up				
**miR-521**	na	>0.05	na	na	na	>0.05	na	na	Up	Up				
**miR-525-3p**	na	>0.05	na	na	na	>0.05	na	na	Up					
**miR-542-3p**	na	>0.05	na	na	na	>0.05	na	na		Up				
**miR-574-5p**	na	>0.05	na	na	na	>0.05	na	na			Up			
**miR-650**	na	>0.05	na	na	na	>0.05	na	na					Up	
**miR-668-3p**	na	>0.05	na	na	na	>0.05	na	na					Down	
**miR-758-3p**	na	>0.05	na	na	na	>0.05	na	na						Up
**miR-93-5p**	na	>0.05	na	na	na	>0.05	na	na				Up/Down		
**miR-299-5p**	na	>0.05	na	na	na	>0.05	na	na	Up					

Table S8. Number of differentially altered miRNAs that target each gene implicated in the Wnt signaling pathway. Data is stratified by cohort and the database that was used for miRNA-target integration. T: Targetscan7.2 (predicted). M: miRTarBase7.0 (experimentally-validated).

Gene Targets	prior PE vs. NT pregnancy (Cohort 1)		prior PE vs. NT pregnancy (Cohort 2)		ACS vs non-ACS (Cohort 3)		current PE vs NT pregnancy (cohort 4; literature)	
	T	M	T	M	T	M	T	M
NFAT5	2	4	7	4	39	17	27	12
CCND2	1	4	3	3	36	35	26	23
SMAD2	3	3	3	2	32	20	20	15
CSNK2A1	3	1	3	3	26	22	16	15
NFATC3	3	1	5	1	19	4	13	1
CCND1	1	3	1	4	16	37	15	26
GSK3B	2	2	2	3	20	23	15	14
PPP3R1	1	1	3	3	23	9	18	8
MAPK8	1	2	2	1	28	13	21	8
PPP3CB	3	1	1	1	11	5	6	3
ROCK2	1	2	2	1	20	7	16	7
TCF7L2	2	1	2	1	16	12	7	8
APC	1	1	2	1	25	9	15	7
PPP2CA	1	1	2	1	17	8	9	5
SFRP1	1	2	1	1	5	12	3	4
TBL1XR1	1	1	2	1	29	19	22	6
PPP2R5C	1	1	1	1	11	9	9	9
FZD4	0	0	3	1	34	8	22	7
TP53	0	0	2	2	12	30	4	17
SMAD4	0	0	1	3	5	21	3	24
FRAT2	0	0	2	2	7	12	8	4
FZD8	0	0	3	1	17	3	6	3
CTNNBIP1	0	0	2	1	19	6	17	6
DVL3	0	0	2	1	12	12	7	8
PPP3CA	0	0	2	1	20	1	13	3
WNT1	0	0	1	2	14	6	9	2
CAMK2G	0	0	2	1	15	3	8	2
PPP2R1B	0	0	1	2	8	7	5	7
TCF7	0	0	2	1	6	4	4	2
EP300	0	0	1	1	10	15	4	7
FOSL1	0	0	1	1	11	7	11	3
RHOA	0	0	1	1	5	16	0	0
NLK	3	2	0	0	33	11	20	6
LRP6	3	2	0	0	35	9	17	7
PRKCA	2	2	0	0	12	5	2	3
CSNK1A1	2	1	0	0	22	11	15	9
FZD5	1	2	0	0	23	10	12	11
PPP2R5E	1	2	0	0	23	8	15	8
ROCK1	2	1	0	0	25	6	16	4
VANGL2	2	1	0	0	17	5	8	3
WNT5A	1	2	0	0	10	8	2	5
CREBBP	1	2	0	0	9	6	4	2
FZD2	1	2	0	0	6	7	4	2
DKK2	2	1	0	0	8	3	3	3
LEF1	1	1	0	0	5	2	5	1
NFATC2	4	1	0	0	27	3	0	0
AXIN1	1	1	0	0	3	3	0	0

Table S8. Continued

Gene Targets	prior PE vs. NT pregnancy (Cohort 1)		prior PE vs. NT pregnancy (Cohort 2)		ACS vs non-ACS (Cohort 3)		current PE vs NT pregnancy (cohort 4; literature)	
	T	M	T	M	T	M	T	M
FZD6	0	0	0	0	10	26	10	19
VANGL1	0	0	0	0	20	8	17	3
PRICKLE2	0	0	0	0	16	7	12	10
PRKACB	0	0	0	0	17	3	16	7
WNT2B	0	0	0	0	22	5	14	2
RAC1	0	0	0	0	11	19	2	7
SMAD3	0	0	0	0	8	16	6	8
DAAM1	0	0	0	0	18	2	15	2
AXIN2	0	0	0	0	9	13	8	6
NKD1	0	0	0	0	17	7	8	4
BTRC	0	0	0	0	12	9	9	4
CCND3	0	0	0	0	12	8	8	6
WNT9B	0	0	0	0	17	5	8	1
MAP3K7	0	0	0	0	7	11	4	8
TBL1X	0	0	0	0	15	3	10	2
MAPK10	0	0	0	0	10	5	11	2
CTNNB1	0	0	0	0	2	15	1	9
WNT4	0	0	0	0	10	4	8	4
FBXW11	0	0	0	0	13	2	9	1
PRICKLE1	0	0	0	0	10	6	6	3
WNT3A	0	0	0	0	11	4	7	3
SENP2	0	0	0	0	11	6	6	1
CAMK2D	0	0	0	0	10	1	10	1
SIAH1	0	0	0	0	11	2	7	2
PRKX	0	0	0	0	6	8	4	3
WNT7B	0	0	0	0	5	6	6	4
DAAM2	0	0	0	0	6	3	8	3
WNT10B	0	0	0	0	10	2	7	1
PPARD	0	0	0	0	5	6	6	2
WNT9A	0	0	0	0	10	1	7	1
CTBP2	0	0	0	0	10	3	4	1
PPP2R1A	0	0	0	0	6	6	4	1
WNT10A	0	0	0	0	4	4	5	4
WNT7A	0	0	0	0	7	4	4	2
FZD7	0	0	0	0	2	8	1	5
SOX17	0	0	0	0	7	4	3	2
WIF1	0	0	0	0	8	1	4	3
WNT3	0	0	0	0	7	3	4	2
PRKCB	0	0	0	0	3	8	1	3
PRKACA	0	0	0	0	4	4	4	1
CHD8	0	0	0	0	6	4	1	1
WNT16	0	0	0	0	4	5	2	1
DKK1	0	0	0	0	2	3	3	3
PLCB4	0	0	0	0	8	1	1	1
PPP2R5A	0	0	0	0	4	1	5	1
SFRP4	0	0	0	0	3	2	4	2
FZD1	0	0	0	0	1	3	1	3
DVL2	0	0	0	0	4	1	1	1
FZD10	0	0	0	0	3	1	2	1
PPP2CB	0	0	0	0	2	3	1	1
RUVBL1	0	0	0	0	2	2	1	1
WNT2	0	0	0	0	1	1	2	2

Table S8. Continued

Gene Targets	prior PE vs. NT pregnancy (Cohort 1)		prior PE vs. NT pregnancy (Cohort 2)		ACS vs non-ACS (Cohort 3)		current PE vs NT pregnancy (cohort 4; literature)	
	T	M	T	M	T	M	T	M
FZD3	0	0	0	0	32	2	0	0
SKP1	0	0	0	0	11	6	0	0
CAMK2A	0	0	0	0	12	3	0	0
PLCB1	0	0	0	0	12	3	0	0
PSEN1	0	0	0	0	10	1	0	0
JUN	0	0	0	0	2	8	0	0
APC2	0	0	0	0	7	1	0	0
TCF7L1	0	0	0	0	4	4	0	0
CTBP1	0	0	0	0	1	5	0	0
PPP2R5D	0	0	0	0	3	3	0	0
CACYBP	0	0	0	0	4	1	0	0
RBX1	0	0	0	0	2	3	0	0
FRAT1	0	0	0	0	1	3	0	0
NFATC1	0	0	0	0	1	3	0	0
PLCB3	0	0	0	0	1	1	0	0
PORCN	0	0	0	0	1	1	0	0
SFRP2	0	0	0	0	1	1	0	0
CXXC4	0	0	0	0	0	0	4	1
DVL1	0	0	0	0	0	0	1	3
LRP5	0	0	0	0	0	0	2	2
PPP3R2	0	0	0	0	0	0	1	1

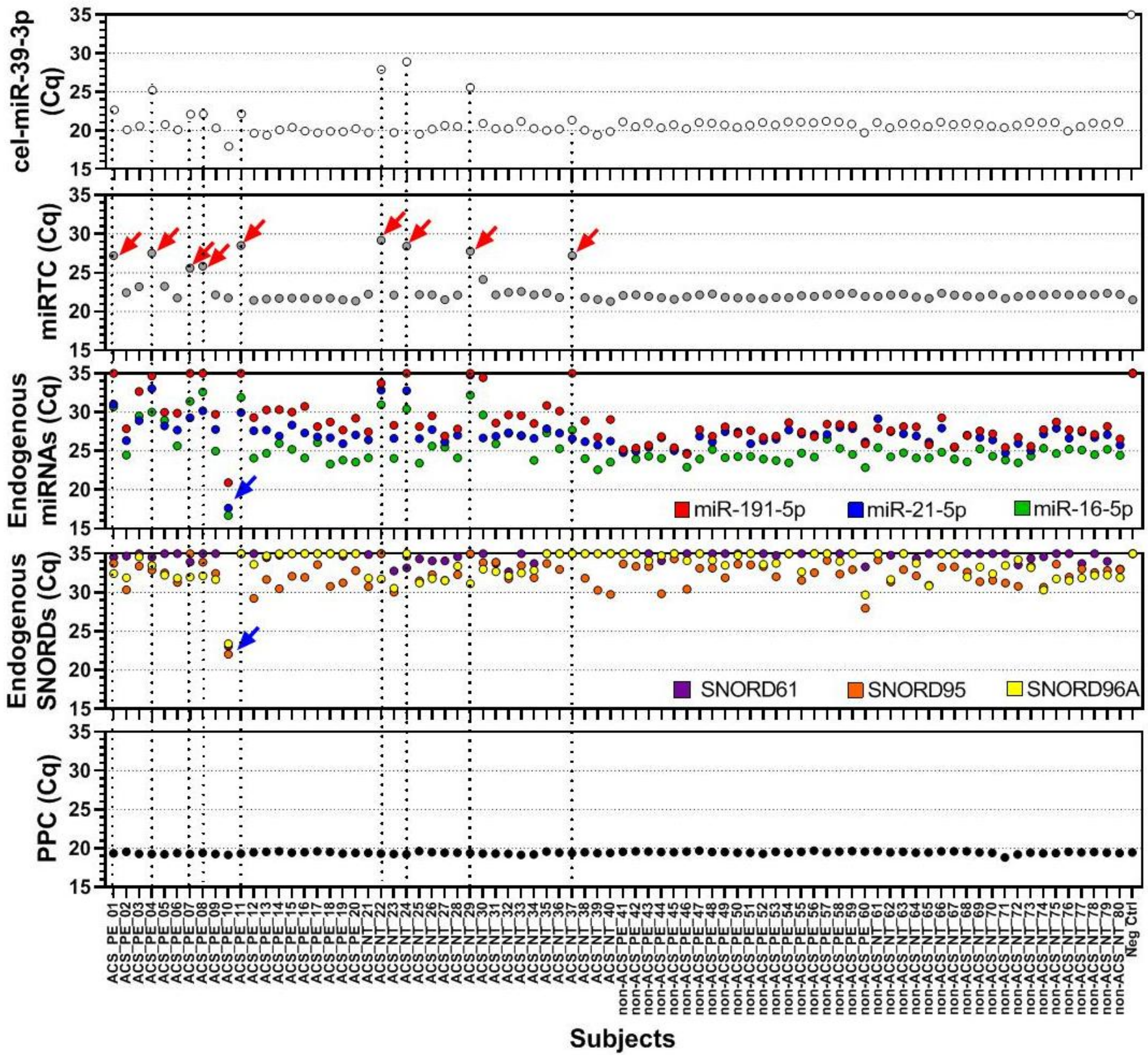

Figure S1. Quality control assessment of total RNA extracted from plasma using the miScript miRNA QC PCR array. The PCR array contains primer assays for several external spike-in controls introduced in fixed quantities at different stages in the RNA extraction and downstream RT-qPCR reactions. Cel-miR-39-3p (a miRNA mimic with no mammalian homolog) is added at the beginning of RNA extraction just after chemical denaturation of nucleases to assess variations in RNA extraction efficiency. miRTC (a synthetic RNA molecule) is incorporated into the reverse transcription reaction to monitor relative reaction efficiencies. PPC is a positive PCR control (synthetic DNA molecule) used to monitor the relative efficiency of downstream PCR reactions. In addition, primer assays for several endogenous miRNAs (i.e., miR-16, miR-21 and miR-191) are included as positive controls since these miRNAs are ubiquitously expressed across many different biologic specimens including body fluids, and several endogenous small nuclear/nucleolar RNAs (i.e., SNORD61, SNORD95, and SNORD96A) are included as negative controls (or markers of cellular contamination) since these are typically expressed abundantly in cells, but relatively poorly in body fluids. Each panel shows the PCR quantification cycles (Cq) assessed for different analytes across all 80 subjects. Each subject is denoted by the cohort (i.e., ACS or non-ACS), prior preeclampsia (PE) or normotensive pregnancy (NT) exposure, and a unique numerical identifier. Red arrows denote subject samples that were flagged for inhibition of the reverse transcription control (miRTC). In most cases these flagged subjects also showed a concomitant decrease in the levels (i.e., higher Cq values) of the external spike-in cel-miR-39-3p and several endogenous miRNAs. Blue arrow denotes a subject sample that showed a marked increase (i.e., lower Cq values) in the levels of both endogenous miRNAs and SNORDs, indicative of potential cellular contamination. Neg Ctrl denotes a mock RNA extraction performed with water instead of plasma. Overall, the majority of samples showed relatively consistent levels in both external and endogenous controls suggesting that the quality and quantity of extracted RNA was generally comparable between samples.

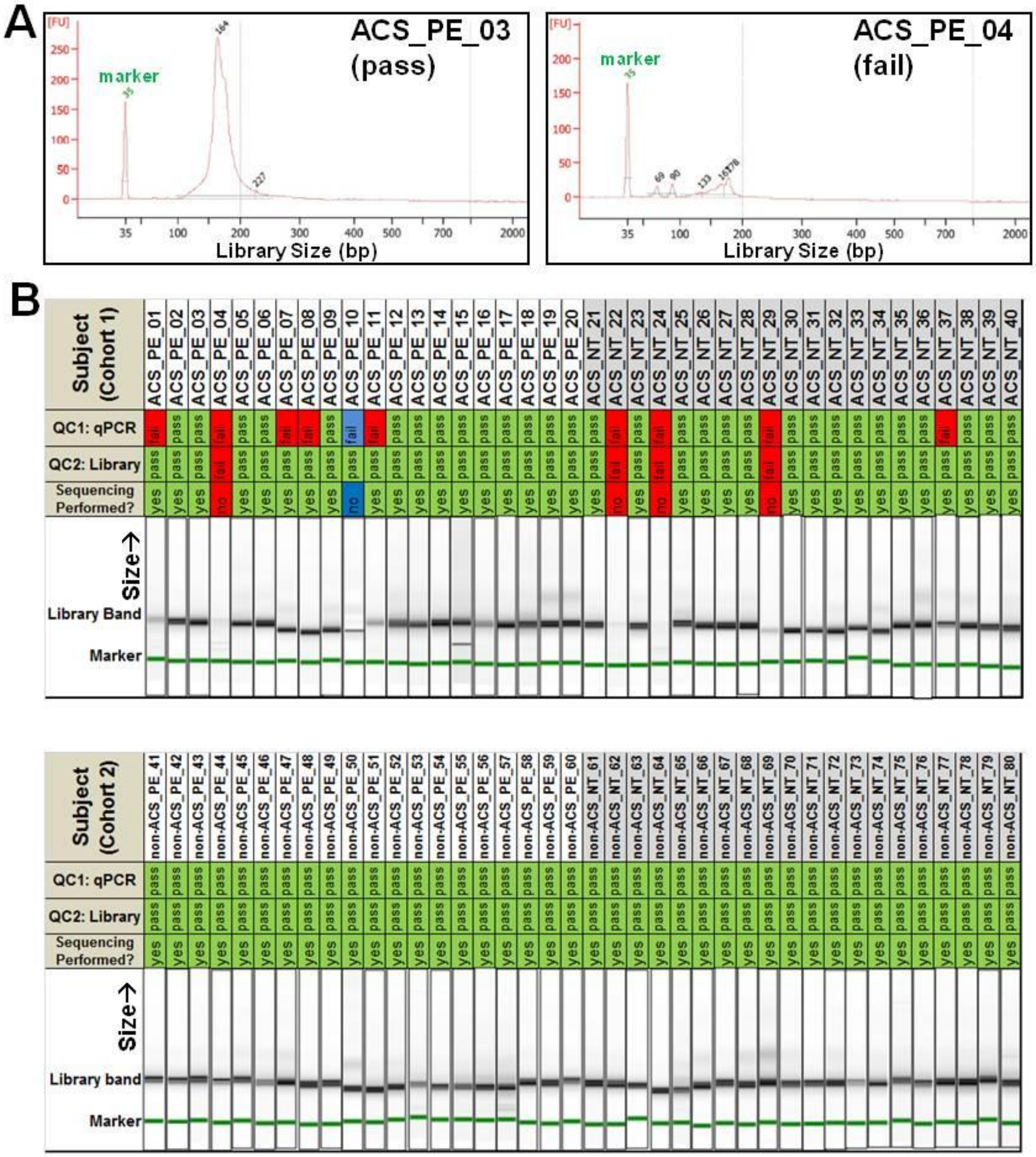

Figure S2. Quality control assessment of sequencing libraries. Library qualification was performed on an Agilent Bioanalyzer using High Sensitivity DNA Chips (A) Electropherogram showing DNA library levels (in arbitrary fluorescence units, FU) versus size in base-pairs (bp). Left panel is an example of a library (generated for subject ACS_PE_03) that showed good yield and was considered to pass this quality control step. Right panel is an example of a library (generated for subject ACS_PE_04) that showed poor yield and quality (consistent with prior PCR QC assays), and therefore was not sequenced. (B) Summary of quality control assays for all 80 subjects. Each subject is denoted by the cohort (i.e., ACS or non-ACS), prior preeclampsia (PE) or normotensive pregnancy (NT) exposure, and a unique numerical identifier. Pass or fail outcomes are indicated for quality control assays conducted with either the plasma-extracted total RNA (i.e., for QC1 using RT-qPCR assays) or subsequently derived next-generation DNA sequencing libraries (i.e., QC2). Four subjects were not sequenced after failing both the PCR and library QC steps. An additional subject (ACS_PE_10; denoted in blue) was not sequenced because it showed evidence of cellular contamination via qPCR assay (i.e., marked elevation in levels of endogenous miRNAs and SNORDs). Virtual gel-like images are presented for each subject to show the quality of the sequencing libraries. Each gel-like image was generated from the corresponding Bioanalyzer electropherogram and manually compiled for presentation. Of note, the extracted RNA from subjects 01,07,08,11 and 37 was flagged for quality issues in the qPCR QC assays, but still generated libraries with sufficient yield/quality to conduct sequencing.

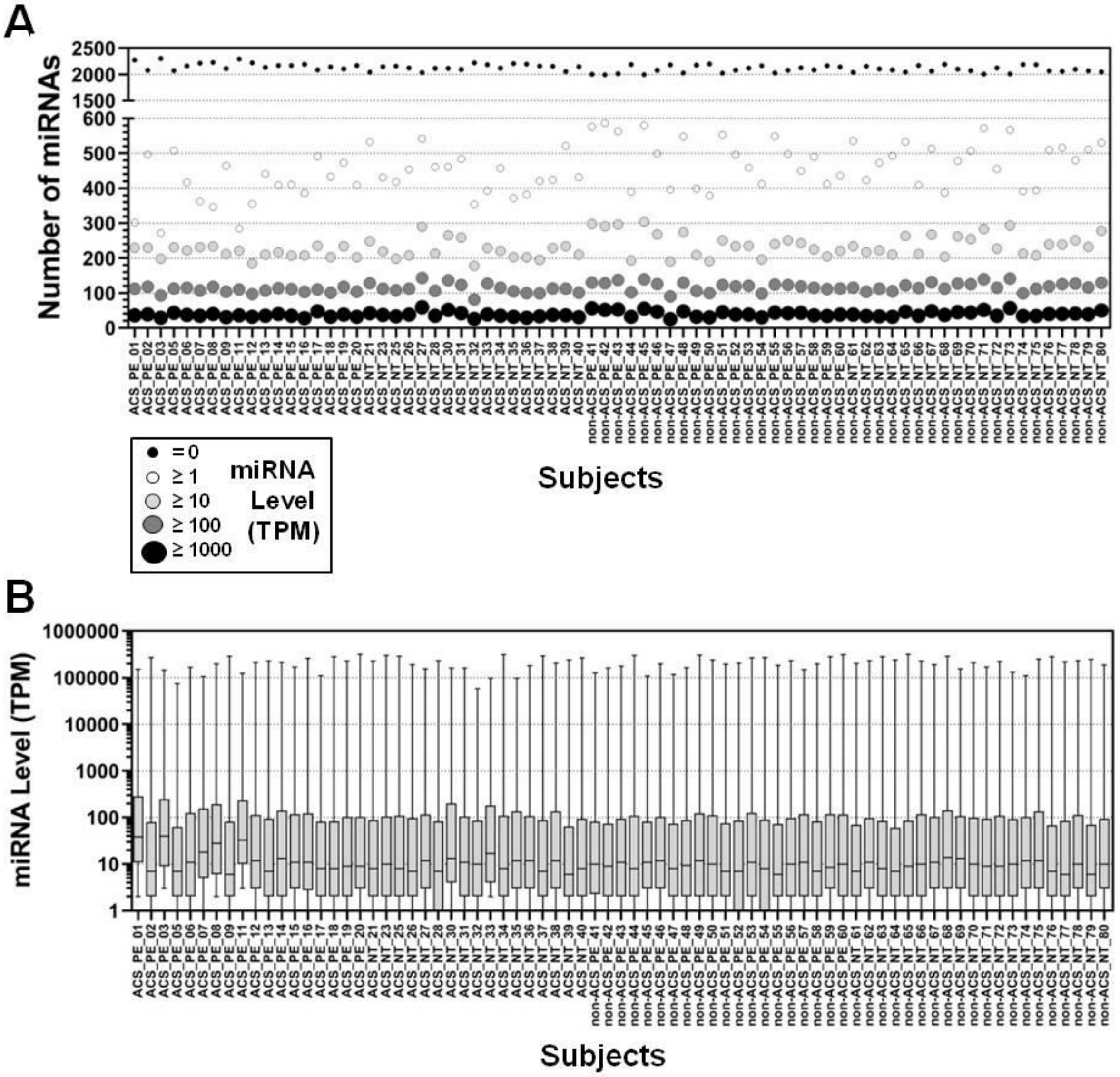

Figure S3. Distribution of plasma miRNA levels per subject identified by RNA-sequencing. (A) Number of miRNAs identified per subject per miRNA level. MiRNA levels are expressed as Tags Per Million mapped reads (TPM), which normalizes for differences in miRNA length and sequencing depth. **(B)** Distribution of miRNA levels per subject. Boxplots show median and interquartile range, and whiskers denote min-max range. MiRNAs with 0 TPM were excluded from the graph. Each subject is denoted by the cohort (i.e., ACS or non-ACS), prior preeclampsia (PE) or normotensive pregnancy (NT) exposure, and a unique numerical identifier.

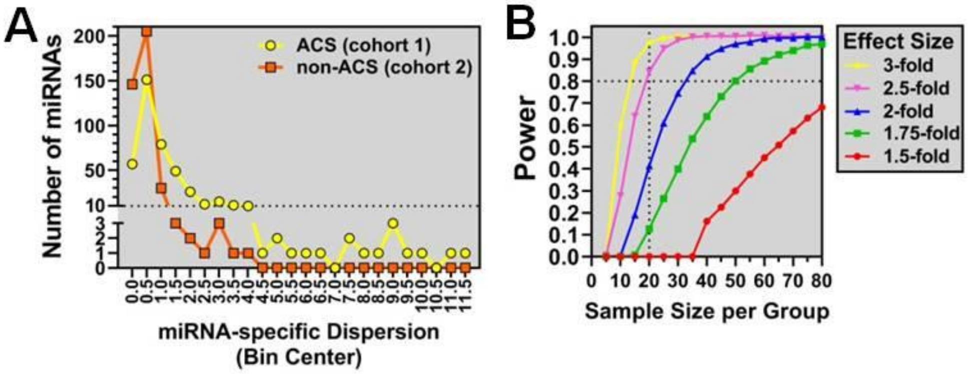

Figure S4. Dispersion of miRNA levels and related power curves. (A) Histogram showing number of miRNAs as a function of their level of dispersion across subjects (bin width = 0.5). miRNA-specific dispersion values were calculated from TMM-normalized counts for 427 miRs in ACS cohort 1 (n=17-18 subjects/exposure group) and 392 miRs in non-ACS cohort 2 (n=20 subjects/exposure group) using the R program RNASeqSampleSize. **(B)** Power curves showing the relationship between statistical power and sample size per group for varying effect sizes (i.e., magnitude of fold-change in miRNA level). Statistical power was estimated by RNASeqSampleSize for a hypothetical scenario assuming 400 assessed miRs, 25 differentially altered miRNAs at FDR<0.05, an average read count of 30 and dispersion level of 0.5.

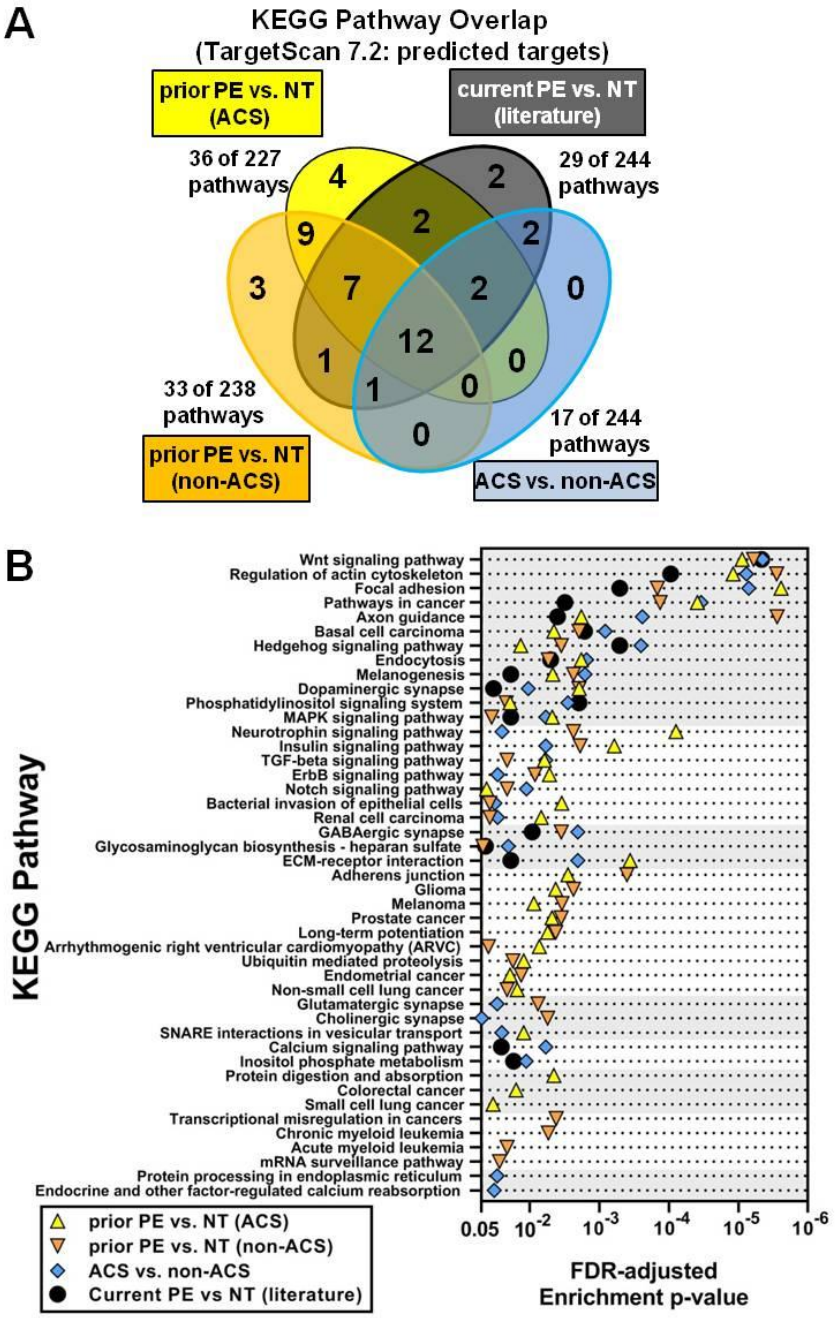

Figure S5. Integration of predicted gene targets and pathway enrichment analysis. Gene target integration was performed with 30 miRs ($p<0.05$), 20 miRs ($p<0.05$), 259 miRs ($FDR<0.05$) and 104 miRs ($p<0.05$) for cohort 1 (prior PE vs. NT exposure; ACS subjects), cohort 2 (prior PE vs. NT exposure; non-ACS subjects), cohort 3 (ACS vs. non-ACS exposure) and cohort 4 (current PE vs. NT exposure, curated from prior literature). **(A)** Venn diagram shows the number of KEGG pathways significantly enriched ($FDR<0.05$) with the predicted gene targets of the altered miRNA candidates (via Targetscan 7.2), and the level of overlap between different exposure groups. (**B)** Identity of specific KEGG pathways from panel A and associated false discovery rate-adjusted enrichment p-values for each exposure group.

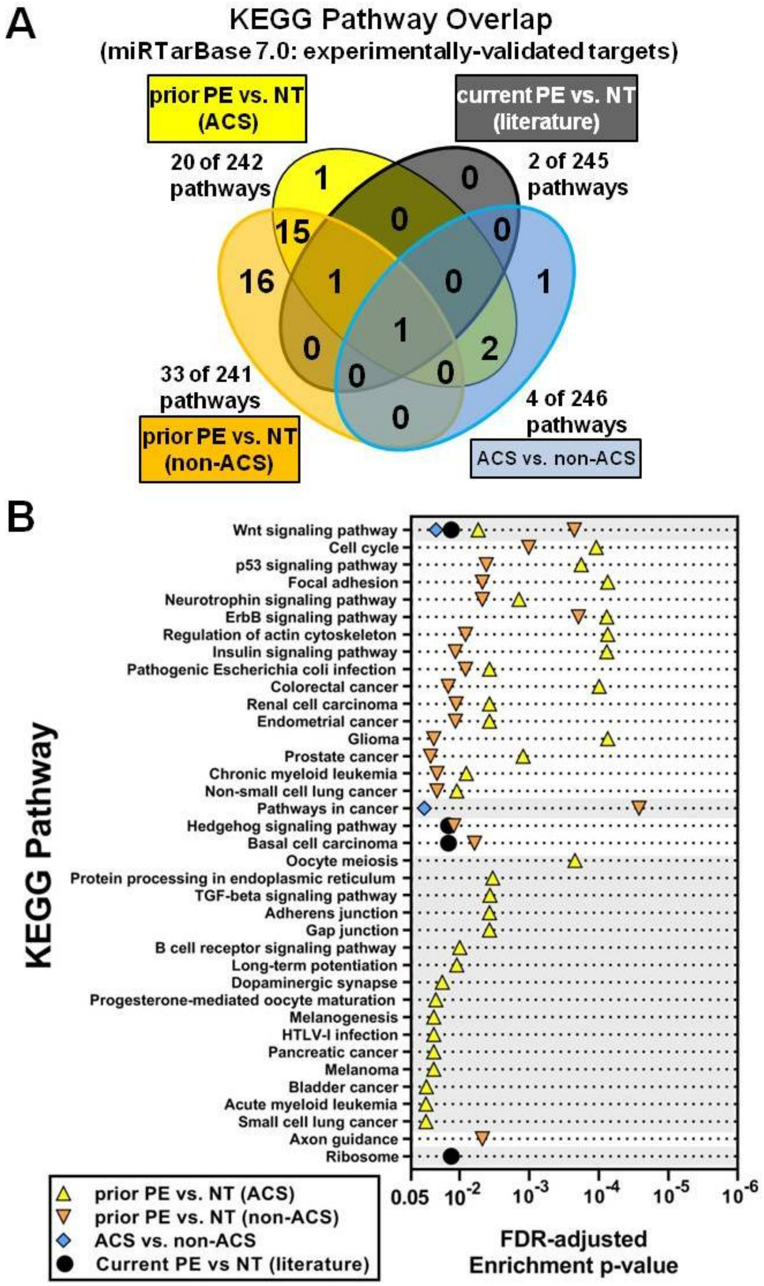

Figure S6. Integration of experimentally-validated gene targets and pathway enrichment analysis. Gene target integration was performed with 30 miRs ($p<0.05$), 20 miRs ($p<0.05$), 259 miRs (FDR<0.05) and 104 miRs ($p<0.05$) for cohort 1 (prior PE vs. NT exposure; ACS subjects), cohort 2 (prior PE vs. NT exposure; non-ACS subjects), cohort 3 (ACS vs. non-ACS exposure) and cohort 4 (current PE vs. NT exposure, curated from prior literature). (**A**) Venn diagram shows the number of KEGG biological pathways significantly enriched (FDR<0.05) with experimentally-validated targets of the altered miRNA candidates (via miRTarBase 7.0 database), and the overlap between different exposure groups. **(B)** Identity of specific KEGG pathways from panel A, and associated false discovery rate-adjusted enrichment p-values for each exposure group.

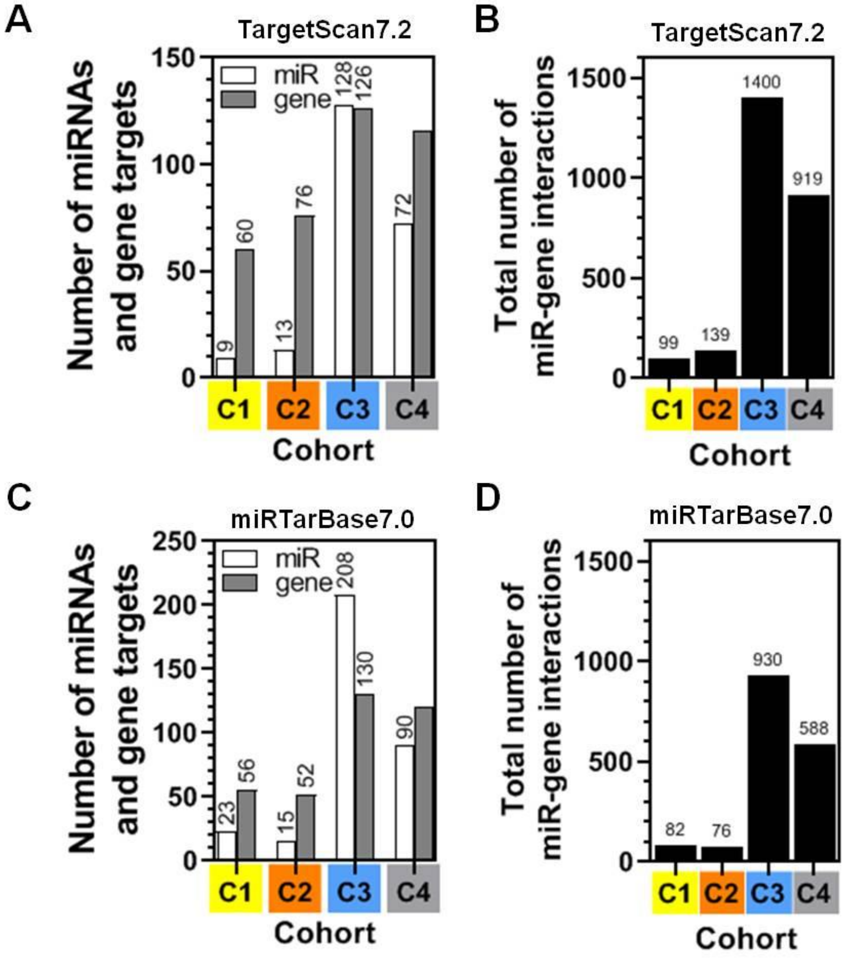

Figure S7. Characterization of miRNA-gene target interactions related to Wnt signaling in subjects with prior PE, current ACS or current PE. (A) Number of miRNAs and predicted gene targets identified in each cohort by Targetscan 7.2. (B) Total number of predicted miRNA-gene target interactions identified in each cohort by Targetscan7.2. (C) Number of miRNAs and experimentally-validated gene targets identified in each cohort by miRTarBase7.0. (D) Total number of experimentally-validated miRNA-gene target interactions identified in each cohort by miRTarBase. Cohort 1 (C1: prior PE vs. NT exposure; ACS subjects), cohort 2 (C2: prior PE vs. NT exposure; non-ACS subjects), cohort 3 (C3: ACS vs. non-ACS exposure) and cohort 4 (C4: current PE vs. NT exposure, curated from prior literature).

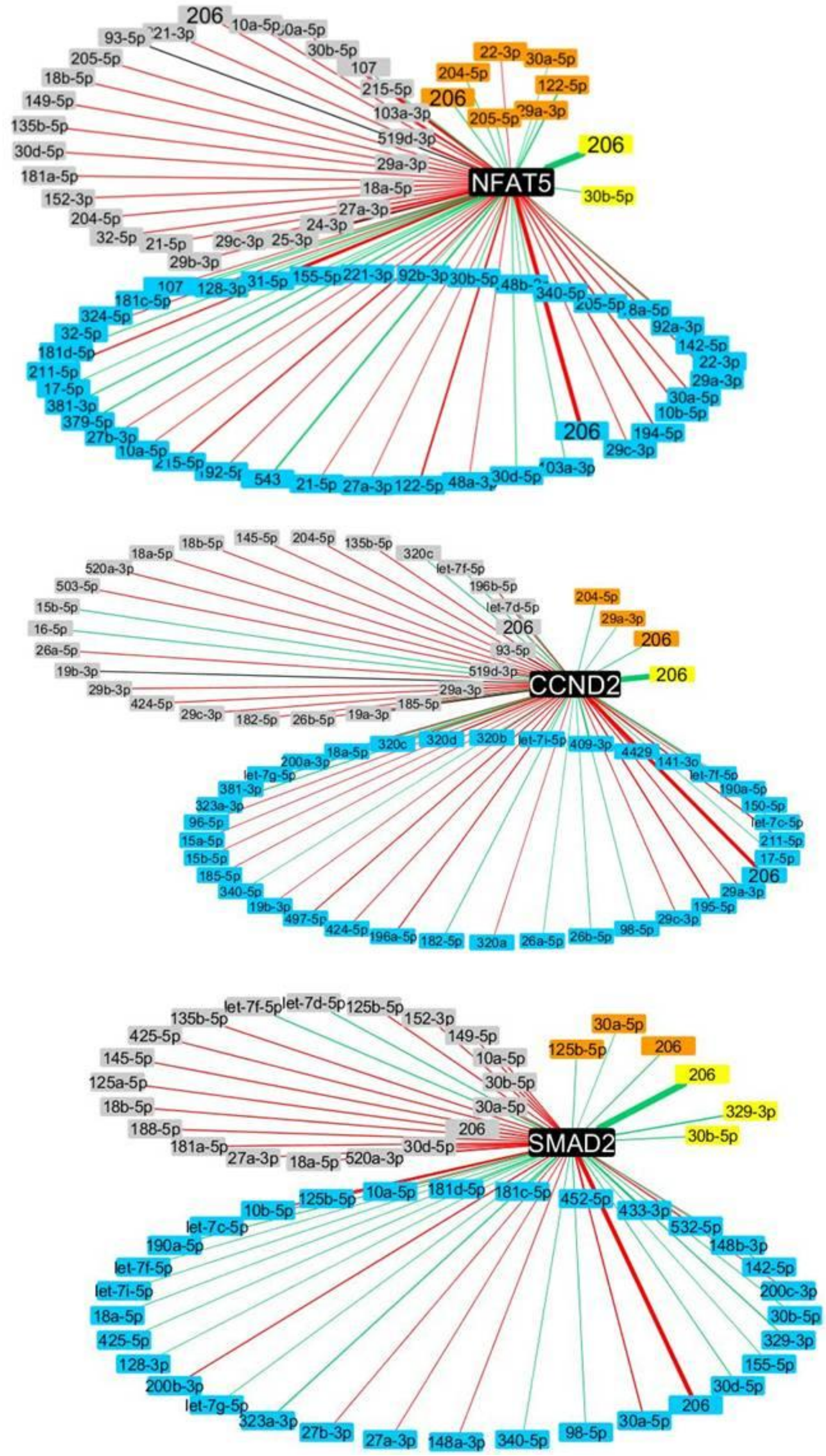

Figure S8. Predicted miRNA-target gene interactions in Wnt signaling associated with PE and ACS. MiRNA interactions with three of the most highly networked genes (NFAT5, CCND2 and SMAD2) identified in Targetscan 7.2. Nodes represent differentially altered miRNAs color-coded according to cohort (cohort 1, yellow; cohort 2, orange; cohort 3, blue; cohort 4, grey) and their common predicted target genes (black nodes). Red and green lines denote increased and decreased miRNA plasma levels, respectively. Line thickness is scale to the magnitude of fold change in miRNA level.

Supplementary References

1. Pilote L, Karp I. GENESIS-PRAXY (GENdEr and Sex determInantS of cardiovascular disease: From bench to beyond-Premature Acute Coronary SYndrome). Am Heart J 2012; 163 (5):741-746 e742.
2. Smith BH, Campbell A, Linksted P, Fitzpatrick B, Jackson C, Kerr SM, et al. Cohort Profile: Generation Scotland: Scottish Family Health Study (GS:SFHS). The study, its participants and their potential for genetic research on health and illness. Int J Epidemiol 2013; 42 (3):689-700.
3. Carty DM, Siwy J, Brennand JE, Zurbig P, Mullen W, Franke J, et al. Urinary proteomics for prediction of preeclampsia. Hypertension 2011; 57 (3):561-569.
4. Sheikh AM, Small HY, Currie G, Delles C. Systematic Review of Micro-RNA Expression in Pre-Eclampsia Identifies a Number of Common Pathways Associated with the Disease. PLoS One 2016; 11 (8):e0160808.
5. Agarwal V, Bell GW, Nam JW, Bartel DP. Predicting effective microRNA target sites in mammalian mRNAs. Elife 2015; 4.
6. Chou CH, Shrestha S, Yang CD, Chang NW, Lin YL, Liao KW, et al. miRTarBase update 2018: a resource for experimentally validated microRNA-target interactions. Nucleic Acids Res 2018; 46 (D1):D296-D302.
7. Dayan N, Schlosser K, Stewart DJ, Delles C, Kaur A, Pilote L. Circulating MicroRNAs Implicate Multiple Atherogenic Abnormalities in the Long-Term Cardiovascular Sequelae of Preeclampsia. Am J Hypertens 2018; 31 (10):1093-1097.
8. Zhao S, Li CI, Guo Y, Sheng Q, Shyr Y. RnaSeqSampleSize: real data based sample size estimation for RNA sequencing. BMC Bioinformatics 2018; 19 (1):191.
9. Li H, Ge Q, Guo L, Lu Z. Maternal plasma miRNAs expression in preeclamptic pregnancies. Biomed Res Int 2013; 2013:970265.
10. Yang Q, Lu J, Wang S, Li H, Ge Q, Lu Z. Application of next-generation sequencing technology to profile the circulating microRNAs in the serum of preeclampsia versus normal pregnant women. Clin Chim Acta 2011; 412 (23-24):2167-2173.
11. Yang S, Li H, Ge Q, Guo L, Chen F. Deregulated microRNA species in the plasma and placenta of patients with preeclampsia. Mol Med Rep 2015; 12 (1):527-534.
12. Ura B, Feriotto G, Monasta L, Bilel S, Zweyer M, Celeghini C. Potential role of circulating microRNAs as early markers of preeclampsia. Taiwan J Obstet Gynecol 2014; 53 (2):232-234.
13. Wu L, Zhou H, Lin H, Qi J, Zhu C, Gao Z, et al. Circulating microRNAs are elevated in plasma from severe preeclamptic pregnancies. Reproduction 2012; 143 (3):389-397.
14. Akehurst C, Small HY, Sharafetdinova L, Forrest R, Beattie W, Brown CE, et al. Differential expression of microRNA-206 and its target genes in preeclampsia. J Hypertens 2015; 33 (10):2068-2074.
